# HDAC inhibition via suberoylanilide hydroxamic acid ameliorates doxorubicin-induced cardiotoxicity

**DOI:** 10.1038/s41467-026-77428-w

**Published:** 2026-09-04

**Authors:** Benay Eksi, Daniel Finke, Synje Michel, Jannek Brauer, Markus B. Heckmann, Mohsen Valadan, Leonard M. Schanze, Vighnesh Sunder, Kevin Steimel, Jürgen Burhenne, Timon Seeger, Hugo A. Katus, Norbert Frey, Johannes Backs, Lorenz H. Lehmann

**Affiliations:** 1 Department of Internal Medicine III: Cardiology, Angiology & Pulmonology, Heidelberg University Hospital, Heidelberg, Germany; 2 German Centre for Cardiovascular Research (DZHK), Partner site Heidelberg/Mannheim, Heidelberg, Germany; 3 Medical Faculty Heidelberg, Institute of Experimental Cardiology, Heidelberg University, Heidelberg, Germany; 4 Internal Medicine IX - Department of Clinical Pharmacology and Pharmacoepidemiology, Medical Faculty Heidelberg/Heidelberg University Hospital, Heidelberg University, Heidelberg, Germany; 5 Department of Internal Medicine VIII, Heidelberg University Hospital, Heidelberg, Germany; 6 Molecular Medicine Partnership Unit, European Molecular Biology Laboratory (EMBL), Heidelberg, Germany; 7 Heidelberg University, Heidelberg, Germany; 8 Helmholtz-Institute for Translational AngioCardioScience (HI-TAC) of the Max Delbrück Center for Molecular Medicine in the Helmholtz Association (MDC) at Heidelberg University, Heidelberg, Germany; 9 German Cancer Research Center (DKFZ), Heidelberg, Germany

**Keywords:** Mechanisms of disease, Target identification, Transcription, Transcriptional regulatory elements, Clinical pharmacology

## Abstract

Anthracycline-induced cardiotoxicity remains a major limitation of cancer therapy, and effective preventive strategies are lacking. Topoisomerase IIb has been implicated as a central driver of this toxicity, suggesting that epigenetic regulators may interfere with the pathological cardiac response. Here, we show that doxorubicin promotes topoisomerase IIb accumulation at cardiomyocyte-specific gene promoters (e.g., *Actc1, Myl2*, and *Myh7)* overlapping myocyte enhancer factor 2 binding sites and enhances myocyte enhancer factor 2 -dependent transcription. This response is attenuated by the pan-histone deacetylase inhibitor suberoylanilide hydroxamic acid. Suberoylanilide hydroxamic acid -mediated cardioprotection requires class IIa histone deacetylases, as genetic loss of HDAC4 abolishes its effect. Mechanistically, suberoylanilide hydroxamic acid induces acetylation of the chaperone 14-3-3, disrupting its interaction with HDAC4/5, promoting their nuclear accumulation, and repressing myocyte enhancer factor 2 - driven transcription. In vivo, suberoylanilide hydroxamic acid mitigates doxorubicin-induced cardiotoxicity. These findings identify histone deacetylase inhibition as a cardioprotective repurposing strategy and reveal a mechanistic link between epigenetic regulation and anthracycline-associated cardiotoxicity.

## Abstract (web summary)

The study identifies a new mechanism of anthracycline cardiotoxicity in vivo and shows that the HDAC-inhibitor SAHA protects the heart by disrupting 14-3-3–HDAC4 signaling, thereby suppressing Topo IIb-linked MEF2-driven transcription.

## Introduction

Despite decades of widespread clinical use, anthracyclines such as doxorubicin remain among the most frequently prescribed anti-cancer agents. However, the molecular mechanisms underlying doxorubicin-induced cardiotoxicity (Doxo-Tox) are still incompletely understood. Current evidence implicates induction of reactive oxygen species (ROS) with subsequent mitochondrial dysfunction and increased apoptosis. This process is probably driven by poised topoisomerase IIb (Topo IIb) and impairments in DNA damage response^1^. To date, the iron chelator dexrazoxan is the only FDA-approved cardioprotective agent so far^2^. Thus, a deeper understanding of the molecular mechanisms is needed to improve the safety of anthracycline-treated cancer patients.

Epigenetic modifiers, such as inhibitors of histone deacetylase activity (HDACi), have served as a novel class of anti-cancer drugs within the last years^3^. Beyond their antiproliferative potential^4^, increasing evidence is coming from preclinical models that HDACis may also exert cardioprotective effects in diverse models of cardiac stress^5,6^. Notably, the pan-HDAC inhibitor suberoylanilide hydroxamic acid (SAHA; vorinostat) has recently been explored as a potential adjunctive therapy to increase the antiproliferative potential of classical treatment strategies of malignant diseases. SAHA has been incorporated into combination therapies to treat non-Hodgkin lymphoma^7^ or multiple myeloma^8^ or sarcoma^9^. Elucidation of a potential cardioprotective mechanism of SAHA may therefore promote such co-treatment strategies to eventually mitigate cardiotoxic effects of anthracyclines.

HDACs are divided into several subclasses with distinct enzymatic properties and functions in the heart. Class I and II HDACs have both been implicated in the regulation of cardiac hypertrophy and pathological cardiac remodeling^10–12^. Class I HDACs, including HDAC1, HDAC2, HDAC3, and HDAC8, have high deacetylase activity and are therefore considered direct enzymatic targets of HDACi. Inhibition of class I HDACs has been linked to altered expression of antihypertrophic genes^12^. In contrast, class II HDACs have more diverse regulatory functions. Class IIa HDACs, including HDAC4, HDAC5, HDAC7, and HDAC9, have limited deacetylase activity and act primarily as signaling-responsive regulators by shuttling from the nucleus into the cytosol^13,14^. They counteract pathological cardiac remodeling by direct inhibition of transcription factors via complexes with other chromatin-modifying proteins. For instance, genetic deletion of the class IIa HDAC “HDAC5” leads to a higher sensitivity to prohypertrophic stimuli in a genetic model of cardiac hypertrophy^15^. The class IIb HDAC “HDAC6” has been implicated in mediating cardiac stress response upon Angiotensin II administration^16^. Because of their reduced deacetylase activity, class II HDACs are unlikely to represent direct enzymatic targets of HDACis^17,18^. They are regulated primarily through non-enzymatic mechanisms. Stimulus-dependent nucleo-cytosolic shuttling depends on their binding to the chaperone protein 14-3-3^19^. When retained in the nucleus, HDAC4 and 5 inhibit the transcription factor myocyte enhancer factor-2 (MEF2)^20^, that is essential for cardiomyocyte hypertrophy and pathological cardiac remodeling. The potential role of class II HDACs in mediating the antihypertrophic effects of HDACis remains incompletely understood.

Here, we identify a previously unrecognized mechanistic link between Topo IIb-dependent DNA damage and MEF2-driven transcriptional remodeling in cardiomyocytes. We show that doxorubicin induces Topo IIb enrichment at MEF2-DNA-binding sites, including promoters of cardiomyocyte-specific and remodeling-associated genes. We further demonstrate that SAHA suppresses MEF2 activity through a mechanism that requires HDAC4 and involves acetylation-dependent disruption of 14-3-3–class II HDAC interactions. Using pharmacological inhibition, genetic loss-of-function models, and in vivo cardiotoxicity assays, we establish that HDAC4 is essential for the cardioprotective effects of SAHA during anthracycline treatment. Together, our findings define the class II HDAC-14-3-3-MEF2 axis as a critical regulator of anthracycline-induced cardiac remodeling and reveal a mechanistic basis for HDAC inhibitor-based cardioprotective adjunctive therapy.

## Results

### Genomic topoisomerase IIb binding identifies cardiac doxorubicin target genes

**Fig. 1:**
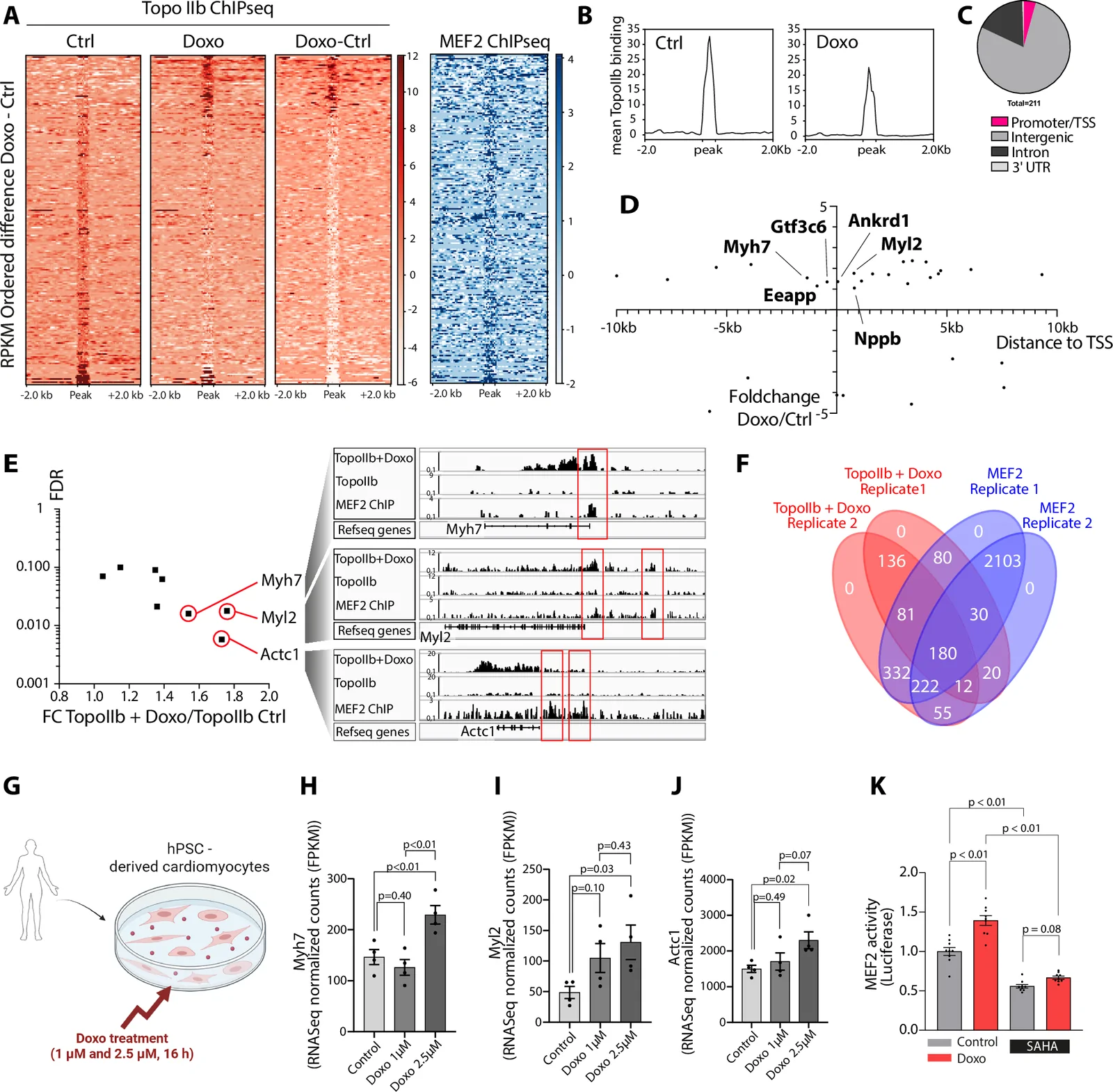
Doxorubicin promotes Topo IIb binding at MEF2-associated genomic regions and activates MEF2-dependent transcription. **A** Heatmaps of topoisomerase IIb (Topo IIb) ChIP-seq enrichment in control (Ctrl) and doxorubicin-treated (Doxo) NRVMs at differentially enriched peaks *(*Doxo vs. Ctrl, two averaged replicates/treatment, FDR < 0.1*)*. Rows correspond to genomic regions. These regions were plotted in the same order for Ctrl (first panel), doxorubicin-treated cells (second panel), and for the difference Ctrl subtracted from Doxo (Doxo-Ctrl) in the third panel. The rightmost heatmap shows MEF2-ChIP-seq enrichment over the same ordered set of regions. **B** Average Topo IIb ChIP-seq enrichment over all differentially enriched peaks in Ctrl and Doxo-treated NRVMs (±2 kb around the peak). **C** Distribution of Doxo-induced Topo IIb peaks to genomic locations. The pie chart shows the respective fraction of peaks located in promoters/transcriptional start sites (TSS), intergenic regions, introns, or 3′ UTRs. **D** Scatterplot highlighting the differential Topo IIb-bound regions after Doxo treatment with the highest fold change (FC) Doxo/Ctrl (*y*-axis). The *x*-axis indicates the genomic distance of the peak to the TSS (in kb). **E** Scatterplot of the most regulated Doxo-induced Topo IIb peaks according to FC and false discovery rate (FDR). Exemplary ChIP-seq tracks are shown for Topo IIb (Ctrl and Doxo) and MEF2 for promoter regions of *Myh7*, *Myl2* and *Actc1*. **F** Topo IIb was binding after Doxo treatment in NRVM on specific sites (409 induced binding sites in the two replicates). The venn diagram shows that a high fraction (180) overlapped with MEF2-binding sites (all replicates). **G** Experimental setting of treating human induced pluripotent stem cells (hiPSC) derived cardiomyocytes (Maillet et al.^21^). Illustration was created using BioRender. Re-analysis of published RNA-seq data in hiPSC-derived cardiomyocytes treated with doxorubicin (vehicle, 1 µM, 2.5 µM; 16 h treatment). Gene expression for *Myh7* (**H**), *Myl2* (**I**) and *Actc1* (**J**) reveals a significant increase after doxorubicin treatment (2.5 µM). Normalized values (FPKM) for *Myh7, Actc1* and *Myl2*; points are replicates (2 sets of duplicates, *n* = 4) and bars show mean ± SEM. Statistical significance is determined by one-way ANOVA followed by Bonferroni’s multiple comparisons test. Exact *p* values for (H) were: Control vs Doxo (1 µM), *p* = 0.4005; Control vs Doxo (2.5 µM), *p* = 0.0055; Doxo (1 µM) vs Doxo (2.5 µM), *p* = 0.0015. **K** Doxo-induced MEF2 activity is reversed by co-treatment with SAHA in hiPSC-derived CMs (*n* = 9 replicates per treatment). Data are presented as mean ± SEM. Statistical analysis was performed using one-way ANOVA followed by Bonferroni’s multiple comparisons test. Exact *p* values for the indicated comparisons were: Control vs Doxo, *p* < 0.0001; Control vs SAHA, *p* < 0.0001; Doxo vs Doxo+SAHA, *p* < 0.0001; SAHA vs Doxo+SAHA, *p* = 0.0829. Source data are provided as a Source data file.

Doxorubicin affects topoisomerase IIb (Topo IIb) activity. To identify downstream targets of doxorubicin, Topo IIb ChIP-seq was performed in neonatal rat ventricular cardiomyocytes (NRVM), that were treated with doxorubicin. We were able to identify genomic regions significantly gaining Topo IIb enrichment after doxorubicin treatment (*n* = 211 peaks, FDR < 0.1), indicative of a specific response to double-strand breaks. These regions comprise cardiomyocyte-specific promoters, e.g., of *Myh7*, *Myl2* and *Actc1* (Fig. 1A–E, Supplementary Fig. 1A–D) and share a high enrichment of the transcription factor myocyte enhancer factor 2 (MEF2), which is known to be a major mediator of pathological cardiac remodeling (Fig. 1A, E). From 1148 called peaks in Doxo-treated NRVMs (at least in one replicate), we found 1012 (88%) peaks overlapping with at least one MEF2-peak. A total of 180 peaks were recalled in all replicates of Topo IIb- and MEF2-ChIP-seq as common targets (Fig. 1F).

### Target genes are regulated in doxorubicin-treated hiPSC

To verify transcriptional dysregulation of the identified genes in human samples, we re-analyzed the publicly available RNA-seq dataset of hiPSC-derived cardiomyocytes exposed for 16 h to vehicle, 1 µM or 2.5 µM doxorubicin (2 sets of duplicate samples per condition, GEO ID GSE106297^21^) (Fig. 1G). We visualized *Myh7*, *Actc1* and *Myl2* by plotting normalized counts (FPKM) across all three conditions (Fig. 1H–J). All three genes showed treatment-associated changes relative to vehicle in a dose-dependent manner. *Myh7* was significantly upregulated at a dose of 2.5 µM compared to the control and 1 µM doxorubicin (*p* = 0.002), while 1 µM of doxorubicin did not lead to changes compared to the control (*p* = 0.401). Genes, such as *Actc1* and *Myl2*, showed similar effects, displaying significant changes at a dose of 2.5 µM compared to the control. To determine whether these transcriptional changes are associated with enhanced MEF2 activity, we performed a MEF2 luciferase reporter assay in hiPSCs treated with doxorubicin in the presence or absence of the HDAC inhibitor SAHA (Fig. 1K). Doxorubicin treatment significantly increased MEF2-dependent transcriptional activity compared with untreated controls (*p* < 0.01). Co-treatment with SAHA significantly attenuated the doxorubicin-induced increase in MEF2 activity (*p* < 0.01), reducing activity to levels that were no longer significantly different from untreated controls (*p* = 0.12). These findings support the hypothesis that anthracycline exposure induces MEF2 activation and suggest that *Myh7, Actc1*, and *Myl2* may serve as downstream indicators of anthracycline-dependent, Topo IIb-guided MEF2 activation. In addition, we examined the autophagy marker LC3B by immunoblotting following treatment with doxorubicin and/or SAHA. Doxorubicin treatment significantly increased LC3BI protein levels compared to the control (*p* < 0.01). Co-treatment with SAHA did not significantly alter the doxorubicin-induced increase in LC3BI expression (*p* = 0.57) (Supplementary Fig. 2).

### SAHA inhibits MEF2 activity in cardiomyocytes

**Fig. 2:**
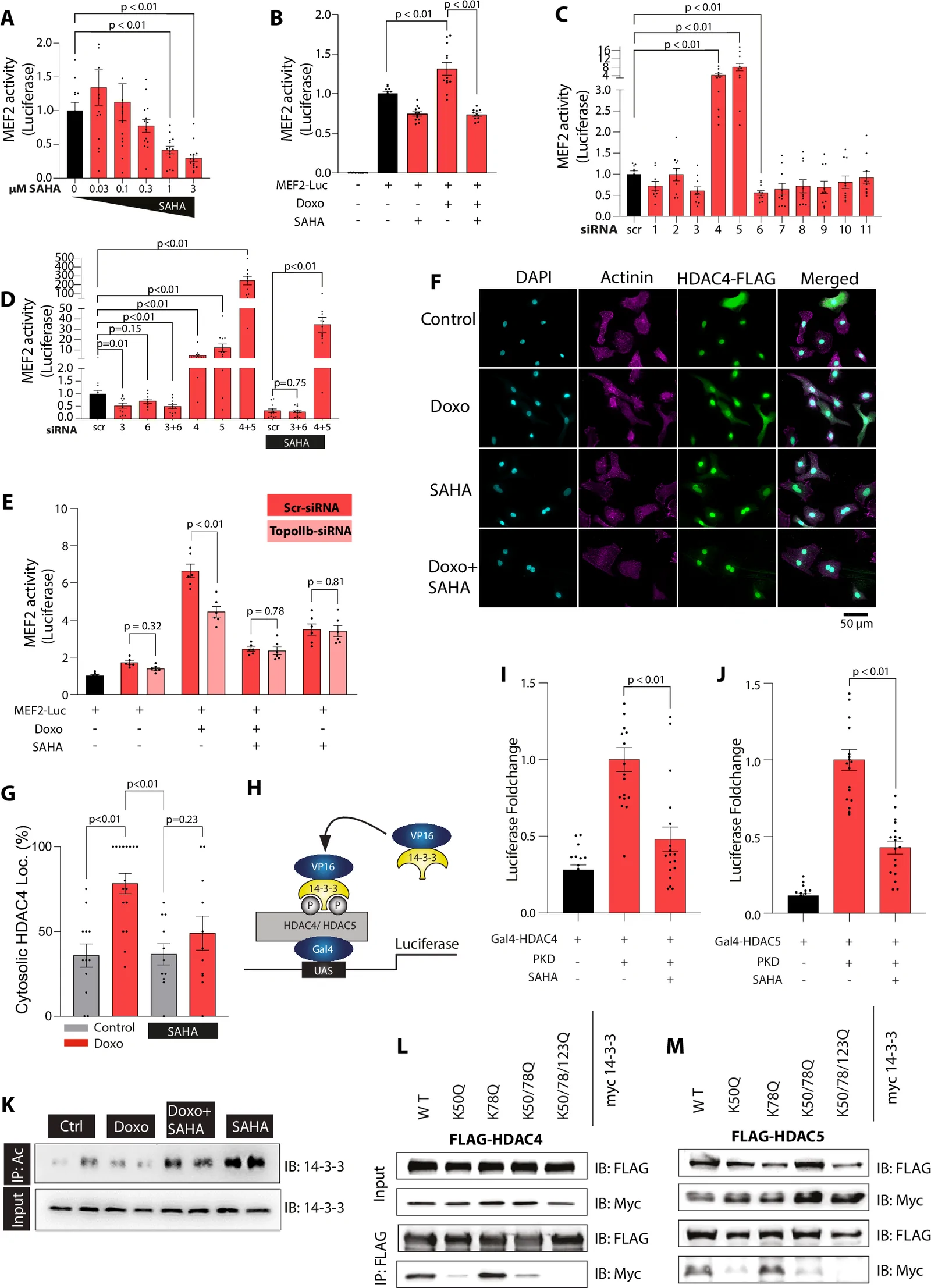
SAHA attenuates doxorubicin-induced MEF2 activation by promoting nuclear HDAC4 retention and disrupting HDAC4 - 14-3-3 binding. **A** Dose response of suberoylanilide hydroxamic acid (SAHA) on myocyte enhancer factor 2 (MEF2) activity measured via luciferase assays. *P* values were calculated via a two-tailed Mann–Whitney test. Bars show mean ± SEM of 14–17 measurements per group from three independent experiments. Exact *p* values for the indicated comparisons were: Ctrl vs 1 µM: *p* < 0.0001, Ctrl vs 3 µM: *p* < 0.0001. **B** Doxorubicin (300 nM) increases MEF2 activity, which is attenuated by co-treatment with SAHA (3 µM), one-way ANOVA followed by Bonferroni’s multiple comparisons test. Bars show values for luciferase signal (mean ± SEM) of 12 measurements per treatment group from three independent experiments. Exact *p* values for the indicated comparisons were: MEF2-Luc vs. MEF2-Luc + Doxo, *p* < 0.0001; MEF2-Luc + Doxo vs. MEF2-Luc + Doxo + SAHA, *p* < 0.0001. **C** siRNA-driven knockdown of HDAC1-11 in NRVMs as indicated. MEF2 activity is shown as luciferase signal. Nine to ten measurements/group from two independent experiments. Data are presented as mean ± SEM. Statistical significance is determined by a two-tailed Mann–Whitney test. Exact *p* values for the indicated comparisons were: Scr vs HDAC4: *p* < 0.0001, Scr vs HDAC5: *p* < 0.0001, Scr vs HDAC6: *p* = 0.0015. **D** Knockdown of HDACs as indicated and treatment with SAHA (3 µM). HDAC3/6 as well as HDAC4/5 were knocked down together, compared to the single knockdowns, respectively. Nine to twelve measurements/group from three independent experiments. Data are presented as mean ± SEM. Statistical significance is determined by a two-tailed Mann–Whitney test. Exact *p* values for the indicated comparisons were: Scr vs HDAC3: *p* = 0.10, Scr vs HDAC6: *p* = 0.1479, Scr vs HDAC3 + 6: *p* = 0.0014, Scr vs HDAC4: *p* = 0.0001, Scr vs HDAC5: *p* = 0.0018, Scr vs HDAC4 + 5: *p* < 0.0001, Scr SAHA vs HDAC3 + 6 SAHA: *p* = 0.7553, Scr vs HDAC4 + 5 SAHA: *p* < 0.0001. **E** Topo IIb knockdown attenuates Doxo-induced MEF2 activity in NRVMs. MEF2 activity measured by luciferase assay after Doxo± SAHA treatment (*n* = 6 replicates per treatment group). Data are presented as mean ± SEM. Statistical significance is determined by one-way ANOVA with Bonferroni posttest. Exact *p* values for the indicated comparisons were: Scr-siRNA vs. TopoIIb-siRNA, Control, *p* = 0.3221; Scr-siRNA vs. TopoIIb-siRNA, Doxo, *p* < 0.0001; Scr-siRNA vs. TopoIIb-siRNA, Doxo + SAHA, *p* = 0.7795; Scr-siRNA vs. TopoIIb-siRNA, SAHA, *p* = 0.8066. **F** Representative immunofluorescence images to detect cytosolic and nuclear HDAC4 localizations of NRVMs treated with doxorubicin (300 nM) ± SAHA (3 µM) stained with DAPI (cyan), α-actinin (magenta) and HDAC4 (green). **G** Quantification of the relative cytosolic HDAC4 localization in percent. Each data point represents the percentage of cells with cytosolic HDAC4 localization within one image (Control, *n* = 12; Doxo, *n* = 17; SAHA, *n* = 11; Doxo+SAHA, *n* = 10). Data are presented as mean ± SEM. Statistical significance is determined by one-way ANOVA followed by Bonferroni’s multiple comparisons test. Exact *p* values for the indicated comparisons were: Control vs. Doxo, *p* < 0.0001; Doxo vs. Doxo + SAHA, *p* = 0.0065; SAHA vs. Doxo + SAHA, *p* = 0.2306. **H** Scheme of the used luciferase reporter assay to evaluate the binding of class II HDACs and 14-3-3 after treatment with SAHA. VP16–14-3-3 was overexpressed together with HDAC4/5-Gal4 and an UAS-reporter. **I** Relative luciferase activity of HDAC4-14-3-3 binding stimulated via PKD co-expression and inhibited with SAHA (3 µM). 18 measurements from three independent experiments. Data are presented as mean ± SEM. Statistical significance is determined by Mann–Whitney *U*-test. The exact *p* value for the indicated comparison was PKD vs. PKD + SAHA, *p* < 0.0001. **J** Relative luciferase activity of HDAC5-14-3-3 binding stimulated via PKD co-expression and inhibited with SAHA (3 µM). 18 measurements from three independent experiments. Data are presented as mean ± SEM. Statistical significance is determined by Mann–Whitney *U*-test. The exact *p* value for the indicated comparison was PKD vs. PKD + SAHA, *p* < 0.0001. **K** Immunoprecipitation of endogenous pan-acetylation sites ± Doxo (300 nM) and/or SAHA (3 µM) pretreatment in NRVMs as indicated. Immunoblotting in pulldown and input for 14-3-3 is shown. IP immunoprecipitated antibody, IB immunoblotted antibody. **L** Immunoprecipitation of FLAG-tagged HDAC4 and **M** Immunoprecipitation of FLAG-tagged HDAC5 with co-expression of myc-tagged 14-3-3 chaperone with the indicated targeted mutations. IP: Immunoprecipitated antibody; IB: Immunoblotted antibody. Source data are provided as a Source data file.

Since the HDACi SAHA is known to protect from pathological cardiac remodeling associated with pressure overload, we assessed its potential for repurposing. Interestingly, in luciferase reporter assays in NRVMs, we found a concentration-dependent inhibition of MEF2 by SAHA treatment (Fig. 2A). The activity of MEF2, in turn, was increased by doxorubicin application and could be inhibited by SAHA (Fig. 2B). Furthermore, we confirmed this effect using the pan-HDAC inhibitor trichostatin A (TSA). Similar to SAHA, TSA (1 µM) effectively inhibited the doxorubicin-induced increase in MEF2-dependent transcriptional activity and restored MEF2 activity to levels comparable to those of untreated controls (Supplementary Fig. 3). Given the fact that SAHA is a pan-HDAC inhibitor interacting with the deacetylase domain, we evaluated MEF2 activity after single knockdown of HDAC1-11 using siRNA to identify the SAHA target HDAC and the strongest MEF2 inhibiting HDAC (Fig. 2C). Efficient knockdown of selected HDACs was confirmed by real-time PCR, using HDAC specific primer, respectively (Supplementary Fig. 4). Intriguingly, the effects on MEF2 diverge markedly between different HDACs. HDAC3 + 6 knockdown reduced MEF2 activity, whereas the knockdown of the class II HDACs HDAC4 + 5 was found to induce MEF2 activity. Combined knockdown of HDAC4 + 5 showed a synergistic effect with an exponential induction of MEF2 activity. Vice versa, HDAC3 + 6 did not show additional synergistic effects (Fig. 2D). Further, doxorubicin-induced MEF2 activity was attenuated after Topo IIb knockdown in NRVMs with or without SAHA (Fig. 2E). Additionally, immunostaining in NRVMs revealed that Topo IIb knockdown attenuated doxorubicin-induced formation of the double-strand break marker γH2AX. Co-treatment with SAHA reduced γH2AX formation in scr-siRNA-transfected cells, and this effect was significantly enhanced after Topo IIb knockdown (Supplementary Figs. 5 and 6). Notably, SAHA further reduced γH2AX in the absence of Topo IIb. Accordingly, in a short-term in vivo model, doxorubicin-induced γH2AX formation was attenuated by co-therapy with SAHA at the protein level. In addition, Caspase-3/7 activity was significantly increased after doxorubicin treatment and reduced to control levels by SAHA co-treatment in vivo. Consistently, TUNEL staining for the detection of apoptosis in mice treated short-term with doxorubicin and/or SAHA showed increased apoptosis after doxorubicin treatment, which was attenuated by SAHA co-therapy (Supplementary Fig. 7).

Knockdowns of HDAC4 + 5 were able to mitigate SAHA-driven MEF2 inhibition, indicating that SAHA-driven MEF2 repression is at least partially dependent on class II HDACs 4 + 5. In contrast, HDAC3 + 6 knockdowns did not additionally repress MEF2 in the presence of SAHA, indicating that HDAC3 + 6 are potential direct targets of SAHA. Immunohistology of FLAG-tagged HDAC4 showed nuclear export of HDAC4 after doxorubicin, which is attenuated by administration of SAHA (Fig. 2F, G). Nuclear localization of class II HDACs is known to mediate cardioprotective effects, including MEF2 inhibition.

### SAHA leads to 14-3-3 hyperacetylation and inhibits 14-3-3–class II HDAC binding

The binding between the chaperone 14-3-3 and class II HDACs is a crucial factor for the cytosolic export of class II HDACs^19^. Using the fusion proteins 14-3-3-VP16 and HDAC4/5-Gal4 in control of the Upstream Activation Sequence (UAS-Enhancer) in a mammalian two-hybrid assay, we validated SAHA-dependent disruption of the 14-3-3-HDAC binding (Fig. 2H–J). Since SAHA may modulate chaperone 14-3-3 activity and the binding to class II HDACs, we analyzed a SAHA-dependent modification of 14-3-3. Therefore, we studied whether endogenous 14-3-3 may be acetylated after treatment with SAHA as a non-histone target. By immunoprecipitation for pan-lysine residue acetylation and immunoblotting for endogenous 14-3-3, we found a profound increase in acetylation of 14-3-3 in NRVMs if pretreated with SAHA. In contrast, doxorubicin treatment alone did not induce detectable acetylation of endogenous 14-3-3. Co-treatment with doxorubicin and SAHA resulted in levels of 14-3-3 acetylation comparable to those observed with SAHA alone (Fig. 2K). In order to mimic acetylation of 14-3-3, we mutated the specific lysine residues (Supplementary Fig. 8) which were earlier suggested to be hyperacetylated upon SAHA treatment^22^. Doing so, we found a profound reduction of the binding between 14-3-3 and HDAC4 (Fig. 2L) or HDAC5 (Fig. 2M) in co-immunoprecipitation experiments. These data indicate that K50 is mainly responsible for the acetylation effects. In contrast, the lysine residue 78 alone could hardly show an effect on protein binding in cardiomyocytes.

### SAHA attenuates doxorubicin-induced cardiotoxicity

**Fig. 3:**
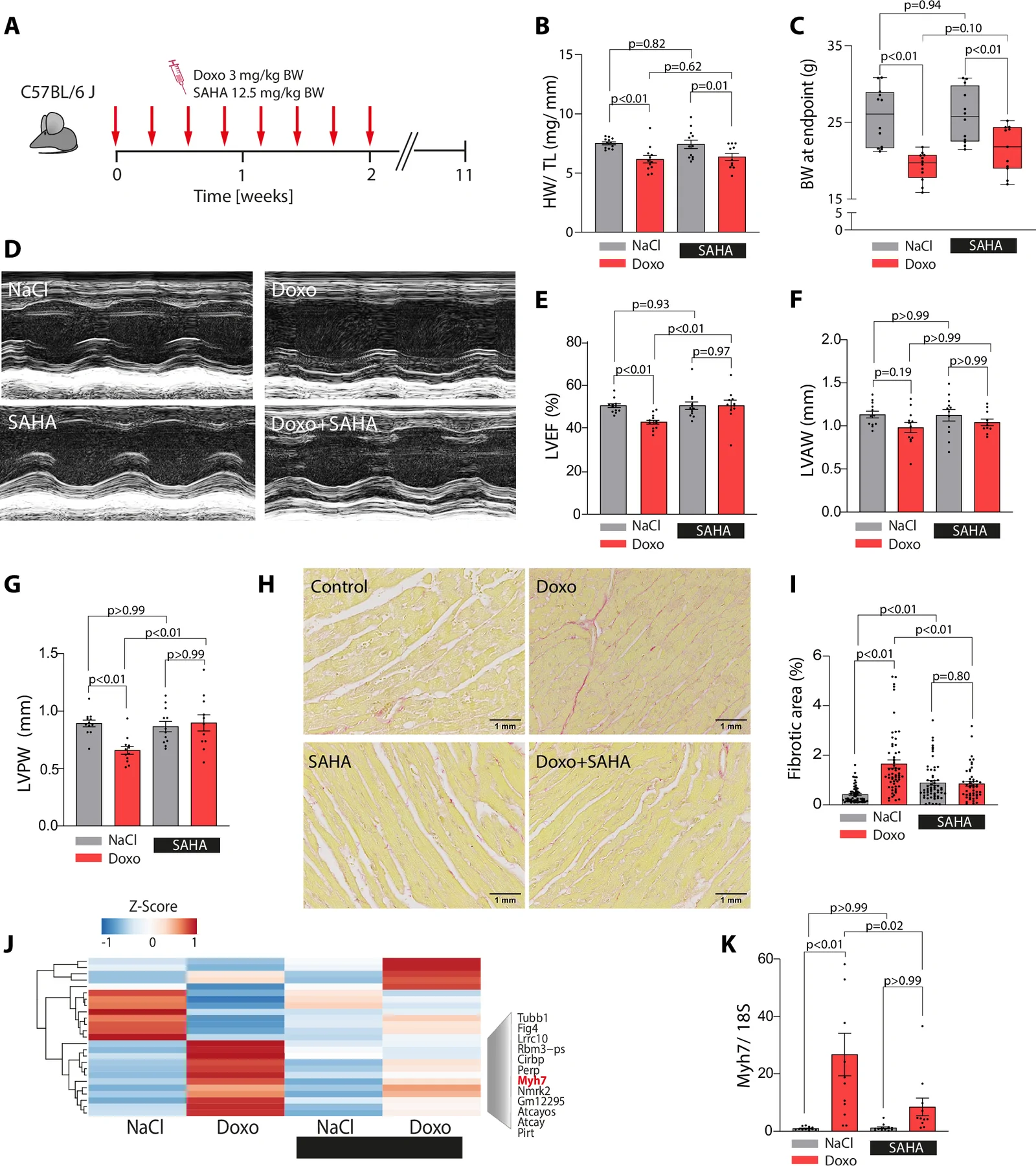
SAHA protects against chronic doxorubicin-induced cardiac dysfunction, fibrosis and transcriptional remodeling in vivo. **A** Graphical illustration of the in vivo model of doxorubicin (Doxo) and SAHA applications in 10-week-old female and male C57BL/6J mice as indicated. 12 mice (6 female and 6 male) were initially allocated to each treatment group. (NaCl, *n* = 12; Doxo, *n* = 12; SAHA, *n* = 12; Doxo + SAHA, *n* = 11). **B** Heart weight (HW) normalized to tibia length (TL). Data are presented as mean ± SEM. Statistical significance was determined by one-way ANOVA followed by Bonferroni’s multiple comparisons test. Exact *p* values for the indicated comparisons were: NaCl vs. Doxo, *p* = 0.0015; NaCl vs. SAHA, *p* = 0.8200; SAHA vs. Doxo + SAHA, *p* = 0.0131; Doxo vs. Doxo + SAHA, *p* = 0.6199. **C** Body weight (BW) at endpoint. Data are presented as box plots, where the center line indicates the median, the box represents the interquartile range (25th−75th percentiles), whiskers indicate the minimum and maximum values, and individual data points are shown. Statistical significance was determined by one-way ANOVA followed by Bonferroni’s multiple comparisons test. Exact *p* values for the indicated comparisons were: NaCl vs. Doxo, *p* < 0.0001; NaCl vs. SAHA, *p* = 0.9380; SAHA vs. Doxo + SAHA, *p* = 0.0019; Doxo vs. Doxo + SAHA, *p* = 0.1000. **D** Exemplary images of echocardiography (M-Mode, short axis, anesthesia with 2% isoflurane). **E** Left ventricular ejection fraction (LVEF) was reduced in Doxo-treated mice and not in SAHA co-treated animals. Data are presented as mean ± SEM. Statistical significance was determined by one-way ANOVA followed by Bonferroni’s multiple comparisons test. Exact *p* values for the indicated comparisons were: NaCl vs. Doxo, *p* = 0.0020; NaCl vs. SAHA, *p* = 0.9254; SAHA vs. Doxo + SAHA, *p* = 0.9708; Doxo vs. Doxo + SAHA, *p* = 0.0017. **F** Left ventricular anterior wall (LVAW) in mm and **G** left ventricular posterior wall (LVPW) in mm. Data are presented as mean ± SEM. Statistical significance was determined by one-way ANOVA followed by Bonferroni’s multiple comparisons test. Exact *p* values for the indicated comparisons in (**F**) were: NaCl vs. Doxo, *p* = 0.0030; NaCl vs. SAHA, *p* > 0.9999; Doxo vs. Doxo + SAHA, *p* = 0.0031; SAHA vs. Doxo + SAHA, *p* > 0.9999 and in (**G**) were: NaCl vs. Doxo, *p* = 0.0030; NaCl vs. SAHA, *p* > 0.9999; Doxo vs. Doxo + SAHA, *p* = 0.0031; SAHA vs. Doxo + SAHA, *p* > 0.9999. **H** Exemplary images of fibrosis with Picro-Sirius Red staining. **I** Quantification of fibrotic area (%). Data are presented as mean ± SEM. Individual data points represent a single region of interest (ROI). Five ROIs per animal were selected for quantification for NaCl, *n* = 12; Doxo, *n* = 12; SAHA, *n* = 12; Doxo + SAHA, *n* = 10 animals. Statistical significance was determined by one-way ANOVA followed by Bonferroni’s multiple comparisons test. Exact *p* values for the indicated comparisons were: NaCl vs. Doxo, *p* < 0.0001; NaCl vs. SAHA, *p* = 0.0024; Doxo vs. Doxo + SAHA, *p* < 0.0001; SAHA vs. Doxo + SAHA, *p* = 0.7944. **J** Differentially expressed genes (DEGs, FDR < 0.05) between Doxo and NaCl treatment, which are further normalized after SAHA treatment. Single gene names are shown. *Myh7* is marked in red. Genes with log2 fc >1 or < −1 are shown. The heatmap represents the Z-score of the mean value per group (*n* = 3 animals/group). **K** *Myh7* expression measured via qPCR, normalized to 18S expression. Data are presented as mean ± SEM. Statistical analysis was performed using one-way ANOVA followed by Bonferroni’s multiple comparisons test; exact *p* values are indicated above the comparisons. Exact *p* values for the indicated comparisons were: NaCl vs. Doxo, *p* = 0.0003; NaCl vs. SAHA, *p* > 0.9999; Doxo vs. Doxo + SAHA, *p* = 0.0185; SAHA vs. Doxo + SAHA, *p* > 0.9999. Source data are provided as a Source data file.

To assess the in vivo relevance of the cardioprotective effects of SAHA, C57BL/6J mice, aged 10 weeks, were co-treated with doxorubicin (total of 24 mg/kg in 2 weeks) and SAHA (100 mg/kg in 2 weeks). The mice were monitored for up to 10 weeks to investigate the long-term effects on pathological gene expression and cardiac function in anesthetized mice (Isoflurane 2%) using echocardiography (Fig. 3A). The treatments had no effect on the survival rate of the animals (Supplementary Fig. 9A). Doxorubicin-treated mice revealed a moderate decrease in heart weight normalized to tibia length (Fig. 3B) and a significant reduction in body weight (Fig. 3C, Supplementary Fig. 10A). Representative images of echocardiography are depicted in Fig. 3D. The left ventricular ejection fraction (LVEF) (Fig. 3E) and heart rate (Supplementary Fig. 10B) demonstrated a mild but consistent decline in doxorubicin-treated mice compared to the control group. The combination therapy of doxorubicin and SAHA preserved cardiac function in the treated mice, as indicated by less pronounced changes in LVEF relative to the doxorubicin-only group (Fig. 3E). Cardiac diameters (mainly LVEDD) showed slightly smaller values in the Doxo+SAHA-treated group compared to the Doxo group (Supplementary Fig. 10C, D), whereas wall thicknesses were decreased after doxorubicin application but normalized with SAHA co-treatment (Fig. 3F, G). In the histologies, cardiac fibrosis was increased in Doxo-treated mice but blunted by SAHA co-treatment (Fig. 3H, I). Transcriptional analysis of heart tissue 10 weeks after the last doxorubicin application revealed clustering of treatment groups (Supplementary Fig. 10E) with a distinct pattern of differentially enriched genes, which was not observed with SAHA co-treatment. Notably, *Myh7*, which we identified as a Topo IIb/MEF2 top target, was among the significantly upregulated genes after doxorubicin (Fig. 3J, K).

### The selective class II HDAC inhibitor TMP195 aggravates doxorubicin-induced cardiotoxicity

**Fig. 4:**
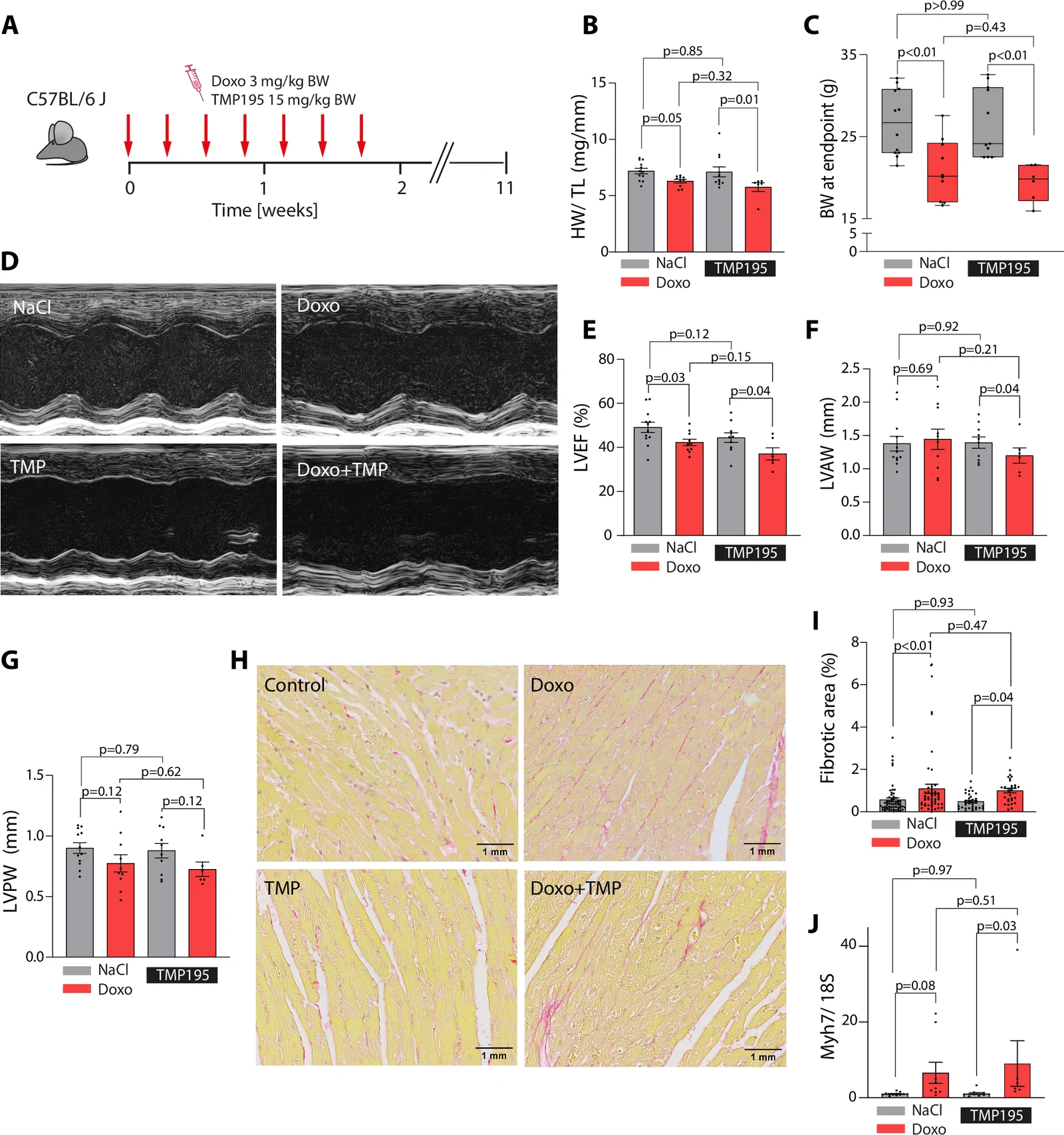
Class IIa HDAC inhibition with TMP195 does not protect against chronic doxorubicin-induced cardiotoxicity in vivo. **A** Graphical illustration of the in vivo model of Doxorubicin (Doxo) and TMP195 (class IIa HDAC inhibitor) applications in 10-week-old female and male C57BL/6J mice. 12 mice (6 female and 6 male) were initially allocated to each treatment group. **B** Heart weight (HW) of the animals that survived (NaCl (*n* = 12), Doxo (*n* = 10), TMP195 (*n* = 11), Doxo+TMP195 (*n* = 6)) normalized to tibia length (TL). Data are presented as mean ± SEM. Statistical significance was determined by one-way ANOVA followed by Bonferroni’s multiple comparisons test. **C** Body weight (BW) at endpoint of survived animals (NaCl (*n* = 12), Doxo (*n* = 10), TMP195 (*n* = 11), Doxo+TMP195 (*n* = 6). Data are presented as box plots, where the center line indicates the median, the box represents the interquartile range (25th−75th percentiles), whiskers indicate the minimum and maximum values, and individual data points are shown. Statistical significance was determined by one-way ANOVA followed by Bonferroni’s multiple comparisons test. Exact *p* values for the indicated comparisons were: NaCl vs. Doxo, *p* = 0.0011; NaCl vs. TMP195, *p* = 0.9914; TMP195 vs. Doxo + TMP195, *p* = 0.0005; Doxo vs. Doxo + TMP195, *p* = 0.4341. **D** Representative images of echocardiography (M-Mode, short axis, anesthesia with 2% isoflurane). **E** Left ventricular ejection fraction (LVEF) shows a reduction in Doxo and Doxo+TMP195 groups. Data are presented as mean ± SEM for NaCl (*n* = 12), Doxo (*n* = 10), TMP195 (*n* = 10), Doxo+TMP195 (*n* = 6). Statistical analysis was performed using one-way ANOVA followed by Bonferroni’s multiple comparisons test; exact *p* values are indicated above the comparisons. **F** Left ventricular anterior wall (LVAW) in mm **G** Left ventricular posterior wall (LVPW) in mm. Data are presented as mean ± SEM for NaCl (*n* = 12), Doxo (*n* = 10), TMP195 (*n* = 10), Doxo+TMP195 (*n* = 6). Statistical analysis was performed using one-way ANOVA followed by Bonferroni’s multiple comparisons test; exact *p* values are indicated above the comparisons. **H** Exemplary images of fibrosis with Picro-Sirius Red staining. **I** Quantification of fibrotic area reveals an increase in Doxo- and Doxo+TMP195-treated mice. Data are presented as mean ± SEM. Individual data points represent a single region of interest (ROI). Five ROIs per animal were selected for quantification. Statistical significance was determined by one-way ANOVA followed by Bonferroni’s multiple comparisons test. Exact *p* values for the indicated comparisons were: NaCl vs. Doxo, *p* = 0.0012; NaCl vs. TMP195, *p* = 0.9274; TMP195 vs. Doxo + TMP195, *p* = 0.0442; Doxo vs. Doxo + TMP195, *p* = 0.465. **J** *Myh7* expression measured via qPCR, normalized to 18S expression. Data are presented as mean ± SEM. Statistical analysis was performed using one-way ANOVA followed by Bonferroni’s multiple comparisons test; exact *p* values are indicated above the comparisons. Source data are provided as a Source data file.

To further evaluate the cardioprotective potential of class II HDACs, SAHA was replaced with the selective class II histone deacetylase (HDAC) inhibitor TMP195 (15 mg/kg), which specifically inhibits HDAC4, HDAC5, HDAC7 and HDAC9. The experimental protocol was slightly modified in duration and treatment dosages (Fig. 4A), since animals treated with Doxo + TMP195 showed higher mortality within the first 2 weeks of TMP195 administration than the TMP195-only group (Supplementary Fig. 9B). Data from the short-term model indicate that TMP195 interferes with the hepatic handling of doxorubicin, leading to increased exposure to doxorubicin and its toxic metabolite doxorubicinol. This pharmacokinetic interaction most likely explains the increased toxicity and mortality observed in Doxo+TMP195-treated animals (Supplementary Fig. 9C–F). The decrease in heart weight normalized to tibia length after doxorubicin treatment was slightly deteriorated with TMP195 co-treatment (Fig. 4B), with a decrease in body weight in the doxorubicin-treated groups (Fig. 4C, Supplementary Fig. 11A). LVEF was significantly reduced by doxorubicin and declined further in the Doxo + TMP195 group (Fig. 4D, E), while heart rate was unchanged (Supplementary Fig. 11B). LVEDD and wall thickness were also decreased in the Doxo + TMP195 groups relative to NaCl controls (Supplementary Fig. 11C, D, Fig. 4F, G). TMP195 did not alleviate doxorubicin-induced cardiac fibrosis (Fig. 4H, I), and TMP195 alone had no significant effects on fibrosis or cardiac function compared with the NaCl group. Additionally, *Myh7* expression, used as a readout of MEF2 activation, was significantly increased in both the Doxo and Doxo + TMP195 groups (Fig. 4J).

### Protective effects of SAHA depend on the presence of HDAC4

**Fig. 5:**
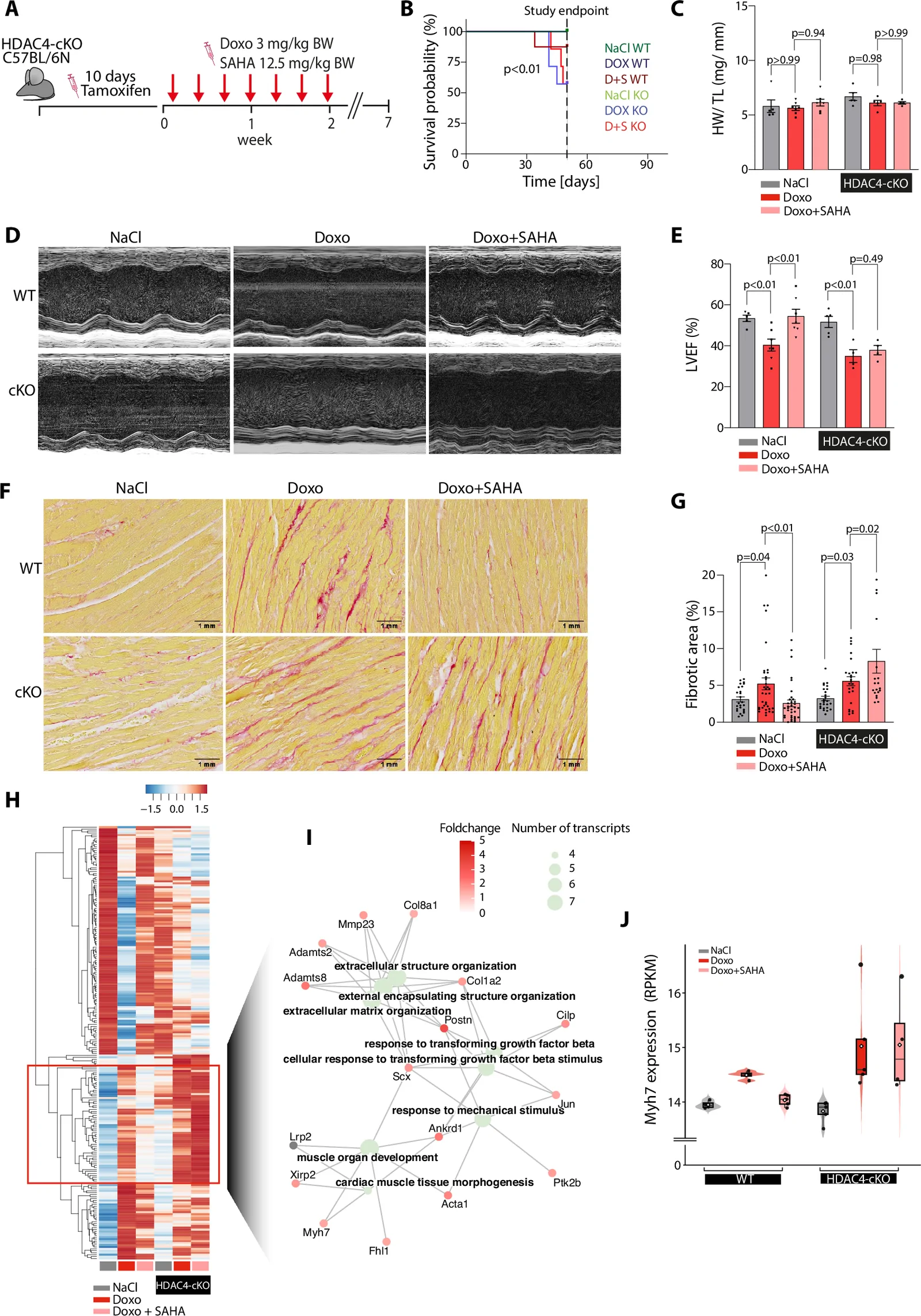
Cardiomyocyte-specific HDAC4-deletion attenuates SAHA-mediated protection against doxorubicin-induced cardiotoxicity. **A** Graphical illustration of the in vivo experimental design showing 10–13-week-old female and male wild-type (WT) littermate controls and HDAC4-cKO mice on a C57BL/6N genetic background. WT littermate controls were treated with NaCl (*n* = 5; 2 females, 3 males), doxorubicin (Doxo; *n* = 7; 2 females, 5 males), or Doxo in combination with SAHA (*n* = 8; 5 females, 3 males), and HDAC4-cKO mice were treated with NaCl (*n* = 5; 3 females, 2 males), Doxo (*n* = 7; 3 females, 4 males), or Doxo+ SAHA (*n* = 7; 4 females, 3 males). **B** Kaplan–Meier survival analysis shows a significant difference in survival probability between the treatment groups (log-rank test for trend, *p* = 0.0097). **C** Heart weight (HW) of the animals that survived normalized to tibia length (TL); WT NaCl (*n* = 5); WT Doxo (*n* = 7); WT Doxo+SAHA (*n* = 7); HDAC4-cKO NaCl (*n* = 5); HDAC4-cKO Doxo (*n* = 5); HDAC4-cKO Doxo+SAHA (*n* = 4); in WT squares: fl^−^/fl^−^ cre+, triangles: fl^+^/fl^+^ cre−. Data are presented as mean ± SEM. Statistical significance was determined by one-way ANOVA followed by Bonferroni’s multiple comparisons test. **D** Representative images of echocardiography (M-Mode, short axis, anesthesia with 2% isoflurane). **E** Left ventricular ejection fraction (LVEF) shows no cardioprotective effect of SAHA in HDAC4-cKO animals. Data are presented as mean ± SEM. Statistical significance was determined by one-way ANOVA followed by Bonferroni’s multiple comparisons test. Exact *p* values for the indicated comparisons were: WT NaCl vs. WT Doxo, *p* = 0.0088; WT Doxo vs. WT Doxo + SAHA, *p* = 0.0007; HDAC4-cKO NaCl vs. HDAC4-cKO Doxo, *p* = 0.0014; HDAC4-cKO Doxo vs. HDAC4-cKO Doxo + SAHA, *p* = 0.4850. **F** Exemplary images of fibrosis with Picro-Sirius Red staining. **G** Quantification of the fibrotic area. WT NaCl (*n* = 5); WT Doxo (*n* = 7); WT Doxo+SAHA (*n* = 7); HDAC4-cKO NaCl (*n* = 5); HDAC4-cKO Doxo (*n* = 5); HDAC4-cKO Doxo+SAHA (*n* = 4). Data are presented as mean ± SEM. Individual data points represent a single region of interest (ROI). Statistical significance was determined by one-way ANOVA followed by Bonferroni’s multiple comparisons test. Exact *p* values for the indicated comparisons were: WT NaCl vs. WT Doxo, *p* = 0.0372; WT Doxo vs. WT Doxo + SAHA, *p* = 0.0047; HDAC4-cKO NaCl vs. HDAC4-cKO Doxo, *p* = 0.0326; HDAC4-cKO Doxo vs. HDAC4-cKO Doxo + SAHA, *p* = 0.0199. **H** Heatmap of differentially expressed genes (DEGs) between NaCl (*n* = 4) and Doxo (*n* = 4) treatment in WT animals (FDR < 0.05). The gene cluster in the red box highlights genes induced by Doxo that are attenuated by SAHA in WT but not in HDAC4-cKO animals. **I** Gene Ontology (GO) network of the genes in the cluster that was identified in (H). Node color reflects the fold-change (Doxo vs. NaCl) and node size indicates the number of transcripts contributing to each GO term. **J** Violin plot shows relative *Myh7* expression (RPKM) for WT NaCl (*n* = 4), WT Doxo (*n* = 4), WT Doxo+SAHA (*n* = 5), HDAC4-cKO NaCl (*n* = 5), HDAC4-cKO Doxo (*n* = 5), HDAC4-cKO Doxo+SAHA (*n* = 4). Violin plots show the distribution of data. Box plots show the median (center), the 25th and 75th percentiles (box), and whiskers extending to the most extreme values within 1.5× the interquartile range. The white diamond indicates the mean. Individual data points represent biological replicates. Source data are provided as a Source data file.

Based on the divergent effects observed with pharmacological class II HDAC inhibition, we next tested whether the cardioprotective effects of SAHA depend on the presence of HDAC4 using a genetic approach. We used a conditional cardiomyocyte-specific knockout mouse model for HDAC4 (HDAC4-cKO)^23^. Efficient deletion of HDAC4 was confirmed by western blotting (Supplementary Fig. 12). In the following, the animals were treated with doxorubicin (21 mg/kg in 2 weeks) and SAHA (87.5 mg/kg in 2 weeks; Fig. 5A). HDAC4-cKO mice treated with doxorubicin exhibited a significant reduction in survival which could not be rescued by co-treatment with SAHA (Fig. 5B). Due to the increased mortality in this genotype, the observation period was shortened from 9 weeks to 5 weeks. During the period, HDAC4-cKO mice showed pronounced weight loss (Supplementary Fig. 13A) whereas no relevant changes in heart weight were observed (Fig. 5C, Supplementary Fig. 13B**)**. In contrast to WT animals, SAHA failed to improve LVEF in doxorubicin-treated HDAC4-cKO mice after 5 weeks (Fig. 5D, E). Heart rate tended to be lower in treated HDAC4-cKO animals (Supplementary Fig. 13C). Moreover, LVEDD and LVESD were significantly higher in HDAC4-cKO following SAHA and doxorubicin co-treatment, even compared to doxorubicin treatment alone (Supplementary Fig. 13D, E). Wall thicknesses were not altered significantly (Supplementary Fig. 13F, G). Notably, doxorubicin-induced fibrosis was further exacerbated in HDAC4-cKO upon SAHA co-treatment (Fig. 5F, G). To assess the transcriptional consequences underlying these phenotypes, we performed RNA-seq analysis. PCAs are shown in Supplementary Fig. 13H–J. Two outliers were excluded from the analysis based on principal component analysis (PCA), as they showed clear separation from the remaining biological replicates. Differential expression analysis revealed a distinct gene expression signature (Supplementary Fig. 13K) that was induced after doxorubicin and normalized by SAHA in control littermates, but remained highly activated in HDAC4-cKO despite SAHA treatment (Fig. 5H). Transcriptional networks derived from GO-terms of this persistently activated gene cluster (marked with a red square) revealed strong enrichment of fibrosis-associated pathways, including “response to mechanical stimulus,” “extracellular matrix organization,” or “cellular response to transforming growth factor beta.” Consistent with our previous data, *Myh7* was found again within this gene cluster, next to e.g., collagens (*Col8a1, Col1a2*) and remodeling-associated transcripts, such as *Mmp23, Adamts2/8* or *Ankrd1* (Fig. 5I, Supplementary Fig. 13L). Looking specifically again at *Myh7*, the course of its regulation resembles the regulation of cardiac fibrosis and shows additional upregulation in HDAC4-cKO animals receiving combined doxorubicin and SAHA treatment (Fig. 5J).

## Discussion

Cardiotoxic side effects remain a major limitation to cancer therapies, creating an urgent need for less harmful drugs^24^ or effective cardioprotective co-therapies. Although oxidative stress and Topo IIb-dependent DNA damage have been implicated, the mechanisms by which these events are translated into sustained maladaptive gene programs that are driving cardiac remodeling remain poorly understood. In this study, we identify a previously unrecognized epigenetic-transcriptional axis that links doxorubicin-induced DNA damage to MEF2-dependent pathological remodeling in cardiomyocytes. In this regard, HDACis, as a relatively new class of anti-cancer drugs, have been shown to exert cardioprotective effects in several preclinical cardiac stress models^5,11,25–27^. However, the specific mechanism by which HDACis protect from pathological cardiac remodeling and cardiac dysfunction remains largely unresolved.

Our findings suggest that doxorubicin-poised Topo IIb DNA-binding preferentially affects MEF2-regulated genomic regions, indicating that anthracyclines do not merely cause random genotoxic stress but instead target transcriptionally relevant regions, that are critical for the cardiomyocyte stress response. This suggests a direct link between doxorubicin-associated activation of maladaptive gene programs (e.g., hypertrophy, fibrosis) and MEF2-dependent transcription. We thereby identify MEF2 as a central integrator of DNA damage. We detected Topo IIb binding at the *Myh7* promoter in vitro, and the transcriptional regulation of *Myh7* observed in our in vivo models is consistent with dysregulated MEF2 activity. Nevertheless, additional transcriptional regulation by other doxorubicin-induced events cannot be excluded. Notably, SAHA co-treatment attenuated doxorubicin-induced γH2AX activation even in the absence of Topo IIb. These findings are consistent with the possibility that the protective effects of SAHA are not solely mediated by inhibition of Topo IIb-dependent DNA damage, but may additionally involve modulation of the HDAC4-MEF2 axis. In this model, nuclear retention of HDAC4 and early inhibition of MEF2 activity may blunt doxorubicin-induced DNA damage signaling and apoptosis, as observed in our in vivo experiments, potentially by limiting ROS-mediated and/or direct DNA damage responses. However, the specific contributions of HDAC4 and MEF2 to this Topo IIb-independent protective mechanism remain to be investigated. As a critical step to regulate nuclear retention of class II HDACs and maintain MEF2 repression, we identified the acetylation of 14-3-3. The lack of 14-3-3 hyperacetylation after doxorubicin treatment alone argues against doxorubicin-induced activation of upstream acetyltransferases as the primary driver of this modification. However, SAHA-induced 14-3-3 hyperacetylation supports a role for non-histone protein acetylation as an important and previously underappreciated mechanism in cardiac stress signaling.

The functional importance of this pathway is underscored by the complete loss of cardioprotective effects of SAHA in cardiomyocyte-specific HDAC4-deficient mice. In this model, SAHA fails to suppress pathological remodeling and instead exacerbates fibrotic response, demonstrating that HDAC4 is an indispensable mediator of SAHA-dependent cardioprotection. Absence of cardioprotective effects in the class IIa-specific inhibitory drug indicates an unresolved non-canonical and potentially cytosolic function of HDAC4 or a required nuclear localization for cardioprotection. The increased TMP195-induced mortality was not accompanied by a further decrease in cardiac function but by elevated liver and plasma levels of doxorubicin and its metabolite doxorubicinol. These data point to a yet unknown drug-drug interaction between TMP195 and doxorubicin and explain the increase in mortality induced by TMP195 in doxorubicin-challenged mice.

The inhibition of HDACs by pan-inhibitors has been shown earlier to have cardioprotective effects in other settings^5,6^. Here, we extend these findings by demonstrating a cardioprotective effect of pan-HDACi in a model of anthracycline-induced cardiotoxicity in vivo. Given its established antiproliferative activity, SAHA represents an attractive candidate for repurposing as a cardioprotective co-therapy in oncological treatment strategies. Importantly, MEF2 activity was inhibited in NRVMs at a SAHA concentration of 3 µM, levels comparable to serum concentrations observed in patients following oral SAHA administration^9,28^. Although the in vitro concentrations of 1–2.5 µM doxorubicin are within the range of clinically observed peak plasma concentrations^29^, the lack of a strictly dose-dependent increase in *Myh7* expression may reflect non-linear transcriptional regulation and/or the timing of sample collection.

However, we cannot exclude the possibility that other targets, inhibited by SAHA^22^, may also contribute to the cardioprotective effects in cardiomyocytes. HDAC6 was shown to be maladaptive in the heart and mediate muscle wasting in response to Angiotensin II in a mouse model. A selective inhibitor of HDAC6, tubastatin A, showed similar protective effects compared to HDAC6 knockout animals^16^. These results support our finding of MEF2 inhibition in HDAC6 knockdown cells. One possible explanation is that HDAC6 contributes to 14-3-3 deacetylation, while HDAC6 inhibition promotes 14-3-3 acetylation. Following our proposed model, this may enhance the cardioprotective effects of HDAC4 and HDAC5 by promoting their nuclear retention. The overlapping effects of SAHA and TSA may be explained by their shared inhibition of class I and II HDACs. However, the HDAC6-selective inhibitor tubastatin A also suppressed MEF2 activity, suggesting that HDAC6 may contribute to 14-3-3 deacetylation in this setting. This interpretation is further supported by siRNA-mediated HDAC6 knockdown in NRVMs, which similarly reduced MEF2 activity. Cardiac remodeling, including fibrosis, was only mild in doxorubicin-treated animals, which suggests a cell-death-independent mechanism of cardiac dysfunction.

Considering the impact of pan-HDACi or selective inhibitors on the heart, the opposing or synergistic roles of several HDACs and their interactions among each other have to be considered in the treatment of heart failure. Our findings suggest that the positive effect of SAHA is likely related to its mode of action to inhibit the deacetylase subunit, which is most relevant in class I HDACs and inhibits class II HDACs less in its protein binding ability^18^. In regard to therapies of malignant diseases, including different HDACis, the cardiac influence of the inhibited HDACs should be considered. A graphical summary is given in Supplementary Fig. 14.

Clinically, these findings have important implications. Importantly, SAHA was administered concurrently with doxorubicin to model its intended use as a cardioprotective co-therapy during anthracycline treatment. Therefore, our study was designed to prevent the initiation of maladaptive remodeling rather than reverse established cardiotoxicity. Accordingly, our findings provide a mechanistic rationale for the use of pan-HDAC inhibitors as cardioprotective co-therapies during anthracycline treatment, while cautioning against selective inhibition of class II HDACs^30^. The pan-HDACi SAHA is already in use for T-cell lymphoma treatment and has been evaluated in other entities, such as sarcoma^9^. The co-therapy with anthracyclines could allow for improving cancer therapy^31,32^, by reducing dose-limiting cardiotoxicity and thereby potentially improving tolerability of anthracycline-based chemotherapy.

Our study identifies HDAC4-14-3-3 acetylation as a critical node in chemotherapy-induced cardiac remodeling and provides a promising therapeutic approach for the development of more specific cardioprotective strategies.

## Methods

### Animal experiments

All animal experiments were approved by the Regierungspräsidium Karlsruhe, Baden-Württemberg, Germany (protocol no. G-161/19) and were conducted in accordance with applicable German animal welfare regulations. Wild-type C57BL/6J mice (*Mus musculus*) were purchased from Janvier Labs. Cardiomyocyte-specific HDAC4-deficient mice (HDAC4-cKO) on a C57BL/6N genetic background were generated by crossing mice carrying a floxed *Hdac4* allele with α-MHC-MerCreMer mice as previously described^23^ and were bred at the central animal facility of Heidelberg University (IBF). Mice were housed under specific pathogen-free (SPF) conditions at 22 ± 2 °C and 50–60% relative humidity under a 12 h light/12 h dark cycle, with food and water available ad libitum. Long-term experiments were performed in mixed cohorts of female and male mice, whereas short-term experiments were performed in male mice only. Animals were randomly allocated to experimental treatment groups, while balancing sex across treatment groups in experiments including both female and male mice. For HDAC4-cKO experiments, age was additionally balanced across experimental groups.

### Long-term animal models

Ten-week-old female and male C57BL/6J mice (Mus musculus) were injected 8 times within 2 weeks with doxorubicin at a concentration of 3 mg/kg BW ± 12.5 mg/kg BW SAHA per injection. Initially, 12 mice, comprising 6 female and 6 male mice, were allocated to each treatment group. All drugs were administered via intraperitoneal (i.p.) injections. Cardiac function was assessed via echocardiography under isoflurane anesthesia (2% v/v). Ten weeks after the last injection, the animals were sacrificed, and downstream analysis was performed. The selective class IIa histone deacetylase inhibitor TMP195 was administered in 10-week-old female and male WT animals (C57BL/6J, Mus musculus) in a dose of 15 mg/kg BW per injection (i.p.), with a total of 7 injections (Doxo, 3 mg/kg BW/per injection) within 2 weeks. Initially, 12 mice, comprising 6 female and 6 male mice, were allocated to each treatment group. Echocardiography under anesthesia was performed at three time points: at baseline before starting the treatment, right after the treatment phase, and at the end of the experiment (9 weeks after the last treatment). Cardiomyocyte-specific HDAC4-cKO mice and corresponding control littermates on a C57BL/6N genetic background (Mus musculus) were 10–13 weeks old at the start of treatment. WT littermates carried either a floxed allele and no MerCreMer (cre−) or no floxed allele but MerCreMer (cre+). Recombination was induced by Tamoxifen application. Tamoxifen was given i.p. over a 10-day period, comprising 5 days of consecutive administration, a 1-day pause, and 4 additional days of treatment. Each animal received a dose of 1 mg/day (10 µg/µl). The cre status of the control littermates is marked in the respective figures. Ten- to thirteen-week-old animals (female and male) received Doxo in a concentration of 3 mg/kg BW ± 12.5 mg/kg BW SAHA per injection, with a total of 7 i.p. injections within 2 weeks. Echocardiography under anesthesia (2% isoflurane) was performed at baseline, right after the treatment phase, and at the end of the experiment. The HDAC4-cKO animals were sacrificed 4 weeks after the last injection.

Animals were euthanized by cervical dislocation, and organs were harvested and weight was assessed using a precision balance (Sartorius). Tibias were separated from the surrounding tissue by H_2_O_2_ digestion.

### Short-term animal model

In the short-term treatment model, 10-week-old male C57BL/6J mice (Mus musculus) were treated with doxorubicin (Doxo) alone or in combination with suberoylanilide hydroxamic acid (SAHA). The numbers of animals used in each experimental group and analysis are indicated in the corresponding figure legends. Doxo (3 mg/kg BW) and SAHA (12.5 mg/kg BW) were administered intraperitoneally (i.p.) on days 1 and 3. Animals were sacrificed on day 4, and hearts were harvested for subsequent analyses. For the analysis of doxorubicin and its metabolite doxorubicinol, a separate short-term experiment was performed in 10-week-old male C57BL/6J mice (Mus musculus). Mice received doxorubicin (3 mg/kg BW, i.p.) alone or in combination with TMP195 (15 mg/kg BW, i.p.) on days 1 and 3. Animals were sacrificed 1, 3, or 6 h after the last injection, and plasma and liver tissue were collected for subsequent UPLC-MS/MS analysis. At the 1- and 3-h time points, *n* = 3 mice per treatment group were analyzed, whereas *n* = 4 mice per treatment group were analyzed at the 6-h time point.

### Genotyping PCR

Genomic DNA for genotyping was isolated from ear punches using lysis buffer (NaOH, 0.5 M EDTA, incubated for 20 min at 95 °C) and neutralization buffer (Tris-HCl, shortly mixed and centrifuged). REDextract PCR Ready Mix (Sigma-Aldrich) was used for the PCR setup. The following primers were used:

**Table:** 

**Primer**	**for**	**1st rev**
HDAC4	ATCTGCCCACCAGAGTATGTG	CTTGTTGAGAACAAACTCCTGCAGCT
		**2nd rev**
		GATTGACCGTAATGGGATAGGTTACG
**Primer**	**1st for**	**1st rev**
MCM-JAX	ATA CCG GAG ATCATG CAAGC	AGG TGG ACCTGA TCATGG AG
**Primer**	**2nd for**	**2nd rev**
MCM-JAX	CTAGGC CAC AGAAIT GAA AGA TCT	GTAGGT GGA AAT TCT AGC ATC ATC C

### Transthoracic echocardiography

Echocardiography was performed using a Visual Sonics Vevo 2100 with an MX550D transducer on anesthetized animals (2% isoflurane). Prior to the echocardiography, the mice were shaved (asid med shaving creme). Measurements of LVEF were done in M-Mode images from the parasternal short axis at the papillary muscle level using Vevo Lab software (version 5.7.1). At least four consecutive beats were used for the measurements.

### Histology

Hearts were fixed in 10% paraformaldehyde and dehydrated in ethanol. Afterwards, they were embedded in paraffin. Sections were stained with Sirius Red. Staining was performed on 1-μm slices as explained before^33^.

In brief, cuts were deparaffinized, rehydrated, and incubated with hematoxylin (5 min, Morphisto) and eosin (2 min, Leica), or Sirius Red (Morphisto), respectively.

Images were taken with the Zeiss Axio Slide Scanner Axio Scan.Z1 (Carl Zeiss, Germany) in brightfield mode with a 4.4 µm/pixel sampling (tile size 1600 × 1200 pixels; field of view 7.04 × 5.28 mm) and 24-bit RGB color. Images were acquired using 1 × 1 binning. No additional image processing was applied prior to quantification, apart from insertion of scale bars and global adjustments of brightness and contrast for figure presentation.

### Drug quantification

Doxorubicin and its metabolite doxorubicinol were quantified by UPLC-MS/MS in the range of 1–1000 ng/mL in plasma and 10–10,000 ng/g in tissue. The respective lower limits of quantification were 1 ng/mL (plasma) and 10 ng/g (tissue). The UPLC-MS/MS system consisted of an ACQUITY Binary Solvent Manager and an ACQUITY Sample Manager coupled to a Xevo TQ-S triple quadrupole mass spectrometer (Waters GmbH, Eschborn, Germany). Data acquisition and quantitative analysis were performed using MassLynx software (version 4.2).

In brief, after addition of the internal standard (idarubicin), the tissue samples were homogenized in water and acetonitrile was added for protein precipitation. After addition of the internal standard, the plasma samples were directly protein precipitated by acetonitrile. Subsequently, the samples were centrifuged and the clear supernatant dried to dryness by nitrogen. The samples were reconstituted by UPLC eluent and directly injected into the UPLC-MS/MS system. Gradient elution on a reversed-phase RP-18 column was performed, and the eluate was directly transferred into the electrospray ion source in positive ionization mode. Sensitive mass transitions for doxorubicin (*m*/*z* 544.1 → 397.1), doxorubicinol (*m*/*z* 546.2 → 399.2), and idarubicin (*m*/*z* 498.1 → 291.1) were selected. The system was calibrated in the respective calibration ranges for plasma and tissue, and the assay fulfilled the recommendations of the FDA guideline on bioanalytical method validation.

### Cell culture and transfection assays

COS-7 cells (ATCC, CRL-1651) were maintained in DMEM with FBS (10%), L-glutamine (2 mM), and penicillin-streptomycin. Transfection of COS-7 cells was performed with Lipofectamine 2000 (Thermo Fisher) according to the manufacturer’s instructions.

### Western blot analysis

Equal amounts of protein lysates (30 μg) were mixed with loading buffer (Laemmli) and denatured at 95 °C for 5 min. Protein samples were loaded and separated by SDS-PAGE according to molecular weight. Following SDS-PAGE, proteins were transferred to a PVDF membrane (Millipore) using wet transfer at 250 mA for 2–3 h. The membrane was blocked with 5% non-fat dry milk in TBST for 1 h. The utilized primary antibodies were anti-HDAC4 (Abcam, ab12172, rabbit pAb), anti-H3 (Abcam, ab70550, rabbit pAb), anti-Phospho-H2A.X (Cell Signaling, 2577S, rabbit pAb), and anti-GAPDH (14C10, Cell Signaling, 2118S, rabbit mAb). The primary antibody, diluted 1:1000 in 2.5% non-fat dry milk in TBST, was incubated overnight at 4 °C. After washing with TBST, the membrane was incubated with secondary anti-mouse IgG, HRP-linked antibody (Cell Signaling Technology, #7076) or mouse anti-rabbit IgG-HRP antibody (Santa Cruz Biotechnology, sc-2357) for 1 h at room temperature diluted 1:2500 in 2.5% non-fat dry milk in TBST for 1 h at room temperature. For primary antibody anti-LC3B (Cell Signaling, 2775S, rabbit pAb, 1:1000), the membrane was blocked with 5% BSA in TBST for 1 h and primary and secondary antibody incubation was performed in 5% BSA in TBST. The detection antibodies were labeled with horseradish peroxidase (HRP) and quantified by subsequent ECL detection.

### Caspase-3/7 activity assay

Caspase-3/7 activity was determined in mouse heart tissue from the short-term mouse model using the Caspase-Glo^®^ 3/7 Assay (Promega, Madison, WI, USA) according to the manufacturer’s instructions. Briefly, heart tissues were homogenized in GST lysis buffer and centrifuged to remove cellular debris. The resulting supernatants were collected, and protein concentrations were determined. Equal amounts of total protein (1 mg) from each sample were used and transferred to white opaque 96-well plates, and an equal volume of Caspase-Glo® 3/7 reagent was added to each well. The reaction mixtures were incubated for 1 h at room temperature in the dark. Luminescence was measured using a multimode microplate reader (VICTOR Nivo™, PerkinElmer, Waltham, MA, USA). Caspase-3/7 activity was expressed as fold change relative to the mean value of the NaCl control group, which was set to 1.

### TUNEL assay

Apoptotic cell death in mouse heart tissue was evaluated using the Click-iT™ Plus TUNEL Assay for In Situ Apoptosis Detection (Thermo Fisher Scientific) according to the manufacturer’s instructions. Briefly, paraffin-embedded mouse heart tissues were sectioned at a thickness of 5 μm, deparaffinized, rehydrated, and permeabilized prior to incubation with the terminal deoxynucleotidyl transferase (TdT) reaction mixture to label DNA strand breaks. The incorporated modified nucleotides were subsequently detected using the Click-iT™ reaction cocktail. Nuclei were stained with DAPI and sections were imaged using a Leica fluorescence microscope. TUNEL-positive nuclei were quantified in 10 randomly selected fields per mouse and data were expressed as the percentage of TUNEL-positive nuclei relative to the total number of nuclei counted. Analyses were performed using tissue sections obtained from three mice per experimental group. Brightness and contrast adjustments were applied for visualization purposes only and did not affect the quantitative analysis.

### Adenovirus production

Adenovirus harboring myosin enhancer factor 2 (MEF2) luciferase was purchased from Seven Hills Bioreagents. Adenovirus harboring HDAC4/HDAC5 constructs were described previously^34^. After generation, the adenoviruses were amplified, purified with the Adeno-X Purification Kit (BD), and their infectious units per µl were determined with the Adeno-X Rapid Titer Kit (BD).

### Culture of neonatal rat ventricular cardiomyocytes (NRVMs)

NRVMs were isolated from 1- to 2-day-old Sprague-Dawley rats as previously described^34^. After isolation, NRVMs were cultivated in complete medium with 10% FBS, 2 mM L-glutamine, and 1% penicillin-streptomycin. NRVMs were infected with ad-HDAC4 virus 24 h after plating. Cells were grown for an additional 24 h in fresh complete medium. The medium was then replaced with serum-free medium 24 h prior to adding test drugs as indicated in the experiments. Cells were then fixed for immunocytochemical staining. For MEF2 luciferase reporter assays, adenoviruses harboring MEF2 luciferase were applied to cultured cardiomyocytes or hiPSCs for 24 h before treating with the indicated chemicals. For overexpression of HDAC4, the HDAC4-FLAG adenovirus was added after cultivating NRVMs or hiPSCs for 24 h. After washing, the cells were treated with the indicated chemicals for 4 h.

### Human-derived iPSCs

The human induced pluripotent stem cell (hiPSC) line SCVI15, derived from a male donor, was generated from peripheral blood mononuclear cells through Sendai virus-mediated episomal reprogramming by the Stanford Cardiovascular Institute Biobank. Differentiation of hiPSCs into cardiomyocytes (hiPSC-CMs) was performed using previously established protocols^35^ with cells between passages 25 and 40. Beating hiPSC-CMs were maintained in RPMI 1640 medium (Thermo Fisher Scientific) supplemented with B27 (Thermo Fisher Scientific). All experiments were conducted using cells between days 35 and 40 following the initiation of differentiation. For experimental assays, hiPSC-CMs were dissociated using TrypLE Select ×10 (Thermo Fisher Scientific) and seeded onto Matrigel-coated six-well plates in RPMI 1640/B27 medium supplemented with 10% KnockOut Serum Replacement (Thermo Fisher Scientific). After 24 h, the medium was replaced with standard RPMI 1640/B27 medium.

### Luciferase assay

Treated NRVMs (Doxo 300 nM, 4 h and SAHA 3 µM, 6 h) were collected in 150 µl lysis buffer (Promega) and transferred to a 96-well plate. Luciferase was added to the samples in a 1:1 dilution. Luciferase signal reads were measured as end-points by the Microplate Reader (*Perkin Elmer*).

### Chromatin Immunoprecipitation

NRVMs (10 million cells) were treated with doxorubicin (1 µM) for 3 h and crosslinked with formaldehyde (1%, diluted in PBS) for 10 min. The reaction was quenched by administration of glycine to a final concentration of 250 mM. Chromatin was isolated and sheared using a Bioruptor (Diagenode) using 3 × 10 cycles (High Power, 30 s power − 30 s pause) as described earlier^36^.

The immunoprecipitation was done with the iDeal ChIP-seq Kit for Histones (Diagenode) using IP-Star (Diagenode) following the manufacturer’s guidelines. Topoisomerase IIb antibody (Santa Cruz, A-12, sc-365071, 3.5 µg) was used for the chromatin pulldown (3.0 µg chromatin).

### ChIP-seq library preparation and next-generation sequencing

ChIP-seq libraries have been prepared using the NEBNext® Ultra™ II DNA Library Prep Kit following the manufacturer’s instructions (New England Biolabs). Bioanalyzer (Agilent Genomics) analysis confirmed the libraries’ quality. Four samples have been sequenced on one lane as 100 bp single-end reads using Illumina HiSeq 2000 (Illumina, San Diego, USA).

### Transcriptional analysis

#### RNA extraction and quantitative PCR

Total RNA was isolated from cells or homogenized cardiac tissue using Qiazol (Qiagen). Samples were incubated with chloroform at room temperature and centrifuged for 15 min at 14,000 rpm. The aqueous phase was mixed with isopropanol and glycogen (Thermo Fisher) and incubated at −20 °C for at least 1 h. The solution was centrifuged for 30 min in order to precipitate the total RNA. Pellets were washed twice with 70%v/v ethanol. RNA concentration was measured by photometry (Nanodrop, Thermo Fisher) and purity was verified (OD_260_/OD_280_ > 1.8). Complementary DNA (cDNA) synthesis of 1 µg of RNA was carried out using a cDNA synthesis kit (Biozym).

Sybr Green reagents (Thermo Fisher) were used for quantitative PCR (ViiA 7 Real-Time PCR System, Applied Biosystems) using the standard curve settings. The following primers were used for the detection of gene expressions:

**Table:** 

Primer	species	For (5′−3′)	Rev (5′−3′)
*18S*	mouse	GCC GCT AGA GGT GAA ATT CTT	CGT CTT CGA ACC TCC GAC T
*Myh7*	mouse	AGA TCC GAA AGC AAC TGG AG	GGA TCT TGC CCT CCT CGT
*HDAC1*	rat	AGC ATC CGG CTT CTG TTA CG	TCA TGA CCC GGT CTG TGG TA
*HDAC2*	rat	GCC AGG GTC ATC CCA TGA A	TCT TCA GCA GTG GCC TTA TG
*HDAC3*	rat	AGT CAG CCC CAC CAA TAT GC	GCC AGA GGC CTC AAA CTT CT
*HDAC4*	rat	TCT TGG GAA TGT ACG ACG CC	AAC ATC CAG GGG TCG CTT TT
*HDAC5*	rat	CTG ACA GCC TTC AGG ACA GTA	CCC AGT GGA GAC AGA TGT CCT T
*HDAC6*	rat	CCA TGC CAT CAA AGA GCA GC	ACG GAA TCA TAG GTG CCT GC
*HDAC7*	rat	ACA GTG TCT GAG CCG AAC CT	TAC TGG GGG AGG AAT CTC CAA
*HDAC8*	rat	GAT CGA AGC ATA TGC CCT GC	CCC TTC TTG GCT GAC CTT CT
*HDAC9*	rat	TCA TTC AAG AAG CGA GTG TTT GA	TCG TGA ATC AGA ATG CGC TG
*HDAC10*	rat	GCC AGG GCA TCC AGT ATA TCT T	TGT CTG CAT CAG ACT CTG GGA
*HDAC11*	rat	AGC ACC TGC CTG ATG TTG TG	CCG GAA AAC CAC TTC ATC CC
*Topo IIb*	rat	ACC GAA AGG TAA AGG CCG AG	TCT TCG GTT TCT TGC TGG CT

#### Bulk-RNA-seq

Total RNA was isolated from cardiac tissue using Qiazol (Qiagen). mRNA-library preparation and RNA-seq was performed by BGI Genomics (Hong Kong) on a DNBSEQ™ platform (20 million reads, 100 bp, paired end). Three animals/group were analyzed in WT Doxo and SAHA treatments (Fig. 3). Five animals/group were analyzed in RNA-seq of HDAC4-cKOs and control littermates (Fig. 5).

#### Immunofluorescence

NRVMs were fixed in 4% paraformaldehyde (Sigma-Aldrich) in PBS, permeabilized with 0.3% Triton X-100 (Sigma-Aldrich) for 10 min at room temperature, and blocked for 60 min with 5% goat serum (PAA) in PBS. Primary antibodies for α-actinin (Sigma) and FLAG were applied in PBS containing 5% goat serum or BSA and 0.05% Triton X-100 for 1 h. Cells were treated with the secondary antibody Alexa Fluor 594-coupled goat anti-mouse (ab150116, pAB, 1:400) and Alexa Fluor 488 coupled anti-rabbit-antibody (Thermo Fisher, A-11008, pAB, 1:400) in PBS containing 5% goat serum and 0.05% Triton X-100 for 1 h. Nuclei were stained with DAPI (Invitrogen, 1:5000). For quantification, blinded >10 fields of view were recorded using a fluorescence microscope (Axio Observer Z1) and the percentage of cells containing HDAC4 in the cytosol for each taken picture was counted. For phospho-H2A.X (γH2AX) staining, cells were fixed, permeabilized, and blocked as described above. Primary antibodies against α-actinin (Clone EA-53, Sigma-Aldrich, A7811, mouse mAb, 1:200) and anti-Phospho-Histone H2A.X (Ser139) (Cell Signaling Technology, 2577S, rabbit pAb, 1:200) were applied in PBS containing 5% goat serum and 0.05% Triton X-100 for 1 h. Subsequent incubation with secondary antibodies and DAPI staining were performed as described above. Images were acquired using a fluorescence microscope (Axio Observer Z1), and a blinded analysis of >10 randomly selected fields of view was performed. Quantification of γH2AX staining was carried out using ImageJ software. Briefly, nuclei were identified and segmented based on DAPI staining, and the integrated density (IntDen) of the γH2AX signal was measured for each nucleus, including background subtraction. The resulting values were plotted as fold change compared to the control Scr-siRNA group, which was set to 1.

#### Mammalian 2-hybrid assay

A mammalian expression vector encoding the GAL4 DNA-binding domain fused to the amino-terminus of human HDAC4 (amino acids 2–740) was generated in the pM expression vector (Clontech). COS-7 cells (ATCC, CRL-1651) were transiently transfected with vectors for GAL4-HDAC4, VP16–14-3-3 and a luciferase reporter gene under the control of 5 copies of a GAL4 DNA-binding site (5 × UAS-luciferase). The expression vectors were transfected in the absence or presence of a construct encoding protein kinase D (PKD). Transfection efficiency was controlled by co-transfection of Renilla luciferase. 24 h after transfection, cells were harvested, and luciferase levels were determined as described above.

#### Co-immunoprecipitation and immunoblotting

COS-7 cells (ATCC, CRL-1651) were harvested 24 h after transfection in Tris (50 mM, pH 7.4), NaCl (150–900 mM), EDTA (1 mM), and Triton X-100 (1%) or in RIPA buffer supplemented with protease inhibitors (Complete; Roche Diagnostics) and PMSF (1 mM). Cells were further disrupted by passage through a 25-gauge needle, and cell debris was removed by centrifugation.

FLAG-tagged proteins were immunoprecipitated using anti-FLAG M2 Affinity Gel (Sigma-Aldrich) and thoroughly washed with lysis buffer. NRVMs were treated with Doxo (300 nM) and SAHA (3 µM) for 1 h for immunoprecipitation of acetylated proteins. Pan-acetyl antibody-bound beads (Immune Chem, ICP0388) were used using the manufacturer’s guidelines with minor adjustments. Briefly, 40 µl of the anti-acetyl lysine agarose was washed three times in 1 ml TBST, twice in 1 ml of 0.1 M NaH_2_PO_4_/1 M NaCl, followed by one wash in 1 ml TBST. 1 mg of protein was incubated with the washed pan-acetyl lysine beads overnight at 4 °C. After incubation, the protein-bead samples were washed four times with TBST.

Bound proteins were resolved by SDS-PAGE, transferred to PVDF membranes, and immunoblotted using an anti-FLAG M2 antibody (Sigma-Aldrich, clone M2, F3165, 1:1000) and an anti-Myc antibody (Cell Signaling Technology, clone 71D10, 2278S, 1:1000) in overexpression experiments. A 14-3-3 antibody (Abcam, ab125032, 1:1000) was used for immunoblotting after pan-acetyl lysine pulldown. Proteins were visualized with a chemiluminescence system (Santa Cruz Biotechnology Inc.).

#### siRNA treatment of NRVMs

Reverse transfection of siRNA duplexes into NRVMs using Lipofectamine 2000 transfection reagent (Thermo Fisher Scientific, Waltham, MA) has been described before^37^. NRVMs (1.5 × 10^6^ cells) were briefly suspended in medium containing 10% FCS, incubated with 100 nM siRNA and transfection reagent, followed by plating in 12-well plates overnight. Medium was changed the next day. The transfection was repeated twice. The sequence of siRNA targeting rat HDAC1-11 and Topo IIb is shown in the table. For Topo IIb knockdown, two different siRNAs targeting Topo IIb (Invitrogen) were used together and are referred to as Topo IIb-siRNA.

**Table:** 

Sense primer	Antisense primer	Locus ID	siRNA ID	Target
UGACAUUGACAUUCACCAUtt	AUGGUGAAUGUCAAUGUCAat	84577	s150967	HDAC1
GGAUCCGGAUGACUCAUAAtt	UUAUGAGUCAUCCGGAUCCta	297893	s136732	HDAC2
CAAUAAGAUCUGUGAUAUUtt	AAUAUCACAGAUCUUAUUGtt	84578	s136737	HDAC3
GGUCAAGGCUUAAGCAGAAtt	UUCUGCUUAAGCCUUGACCgt	363287	s221506	HDAC4
GGACAGUAGUGAUGCCCAUtt	AUGGGCAUCACUACUGUCCtg	84580	s136738	HDAC5
GCACCUAUGAUUCCGUUUAtt	UAAACGGAAUCAUAGGUGCct	84581	s136742	HDAC6
GUGUCUGAGCCGAACCUGAtt	UCAGGUUCGGCUCAGACACtg	84582	s136744	HDAC7
GCAUAAACAAAUGAGGAUAtt	UAUCCUCAUUUGUUUAUGCag	363481	s170821	HDAC8
GCGCAUUCUGAUUCACGAAtt	UUCGUGAAUCAGAAUGCGCtg	687001	s189862	HDAC9
GGGCAUCCAGUAUAUCUUUtt	AAAGAUAUACUGGAUGCCCtg	362981	s170149	HDAC10
CGGGCAUUGUGAAGAGGGAtt	UCCCUCUUCACAAUGCCCGct	297453	s150713	HDAC11
CCUAAUACAAAGAAAGUGAtt	UCACUUUCUUUGUAUUAGGtt	361100	s166822	Topo IIb
CGACAAUAAUGAUUUAGAAtt	UUCUAAAUCAUUAUUGUCGtc	361100	s166823	Topo IIb

A non-targeting sequence (cat#12935300, Thermo Fisher) was used as a control siRNA.

#### Statistical analyses

For all statistical analyses, the software GraphPad Prism (version 10.4.2) was used. Comparison between two groups was performed using the Mann–Whitney *U*-test or Student *t*-test, while for comparison of more than two groups, one-way ANOVA with Bonferroni correction was used to assess statistical differences. A *p-value of* less than 0.05 was considered statistically significant. The number of replicates per group and the statistical test used are stated in the figure legends or the corresponding text. In animal experiments, sample sizes were calculated via assumed effect sizes from previous experiments using G-power (version 3.1.9.6). We chose a significance level of *α* = 0.05 (*α*-error) and a power of 0.8 (1-β-error). In cell culture experiments, sample sizes were estimated based on previous experiments using comparable experimental designs and were chosen to provide sufficient biological replication.

#### Analysis of histology

Analysis of histological images for fibrosis measurements was performed using ImageJ (version 1.54). Images were converted to 8-bit black and white images. The same intensity threshold to assess fibrosis was used in all analyzed images. One heart section per mouse was stained, and five regions of interest (ROI) per heart section were selected randomly. For each ROI, the area of fibrosis was measured (same cutoff for all) and normalized to the whole area of the ROI (in %). The number of replicates analyzed per group are shown in the figure legend. Each data point in the figures represents a single ROI. For statistical comparison, one-way ANOVA with Bonferroni correction was performed using GraphPad Prism version 10.4.2.

#### Analysis of ChIP-seq

FASTQ data were annotated to the rat genome (rn5) using bowtie2 (version 2.3.4.1). Only uniquely mappable reads have been subjected to further analysis. Peak calling was performed with MACS2 (version 2.1.1) with a selected *p* value of 0.0001 and normalization to the respective input file. Differentially enriched peaks were calculated with DESeq2 (version 1.24.0). An FDR < 0.1 was used as a cutoff.

Heatmap and profiles of genomic enrichment were illustrated in Deeptools (version 3.2.1). Exemplary sequencing tracks were visualized in the IGV browser (version 2.13.1) as reads per kilobase per million (RPKM) and 50-bp binning. Files were created from BAM files via the bamCoverage tool. The input file was subtracted from ChIP (bigwigCompare), and files were averaged (bigwigAverage). Principal component analysis (PCA) was performed with multiBamSummary and plotPCA (version 3.5.4), with a binning size of 10,000 bp and a distance between bins of 0 (Supplementary Fig. 1A). Annotated peak numbers and fraction of reads in peaks (FRiP) scores are listed in Supplementary Table 1. Scatterplots and pie charts were created in GraphPad Prism (version 10.4.1). ChIP-seq peaks were annotated to genomic locations and features (e.g., transcription start site (TSS), intergenic, intragenic) with HOMER (v. 4.10) using the annotatePeaks.pl command (Supplementary Data 1).

#### Analysis of RNA-seq

FASTQ files of RNA-seq reads were aligned to the mouse genome version mm9 using bowtie2 (version 2.3.4.1). Counts per transcript were extracted with featureCounts (Rsubread). Raw gene counts were used for differential expression analysis with DESeq2 (version 1.40.2). Genes with an FDR < 0.05 were considered differentially expressed. Reads per kilobase per million (RPKM) values were used for visualization of gene expression where indicated (Supplementary Data 2 and 3).

PCA of normalized RNA-seq expression profiles was conducted prior to downstream analyses to assess treatment-related clustering and sample quality within the Deseq2 package. Two samples were excluded from downstream analysis because they showed clear separation from the corresponding biological replicate groups in PCA. Differentially expressed genes (DEGs) were calculated between two groups (WT Doxo vs NaCl) using the DESeq2 algorithm (Supplementary Data 4 and 5). The set of DEGs (FDR < 0.05) was visualized across all treatment groups in a heatmap using the Chi-squared (Chisq) distance on expression profiles for clustering in the heatmap3 package (version 1.42.1). Gene Ontology (GO) enrichment analysis was performed for the categories biological process (BP), molecular function (MF), and cellular component (CC) using the R package clusterProfiler (version 4.8.3). *P* values were adjusted for multiple testing via the Benjamini-Hochberg method. GO terms with a *p* value ≤ 0.01 and *q* value ≤ 0.05 were considered as significantly enriched. The pathways were visualized as dotplots and cnetplots using the DOSE package (version 3.26.2).

#### Analysis of external data

Data from hiPSC-cells were obtained from Gene Expression Omnibus (GEO ID GSE106297)^21^ and re-analyzed with normalized count matrices (Fragments Per Kilobase of transcript per Million mapped reads, FPKM). hiPSCs were differentiated into cardiomyocytes using the protocol from Lian et al.^38^: Wnt activation (CHIR99021) followed by Wnt inhibition (IWP2) in serum-free RPMI/B27 medium, yielding beating clusters after 7–14 days, which corresponds to a fetal-like cardiomyocyte state. Reads were aligned to the human genome (hg38) for gene annotation. Statistical analysis was performed by one-way ANOVA with Bonferroni correction using GraphPad Prism (version 10.4.2).

### Reporting summary

Further information on research design is available in the Nature Portfolio Reporting Summary linked to this article.

## Supplementary information

Supplementary Information

Description of Additional Supplementary Files

Supplementary Data 1

Supplementary Data 2

Supplementary Data 3

Supplementary Data 4

Supplementary Data 5

Reporting Summary

Transparent Peer Review file

## Source data

Source data

## Data Availability

Raw and annotated ChIP-seq Topo IIb data, including bigwig files underlying Fig. 1 and Supplementary Fig. 1B–D, have been submitted to the EMBL-EBI database (accession number: E-MTAB-16326, https://www.ebi.ac.uk/biostudies/arrayexpress/studies/E-MTAB-16326?key=22bfe984-52bf-4f24-8187-14320db2ab71). MEF2-ChIP-seq was published earlier [accession number: E-MTAB-6213]. The previously published RNA-sequencing dataset of hiPSC-derived cardiomyocytes re-analyzed treated with doxorubicin or vehicle is available in the Gene Expression Omnibus (GEO) under accession code GSE106297^21^. Bulk-RNA-seq data from hearts from doxorubicin-treated HDAC4 cKO animals and controls are deposited by the following accession number: E-MTAB-16361. Source data are provided with this paper.
