# Supplementary material for: HDAC inhibition via suberoylanilide hydroxamic acid ameliorates doxorubicin-induced cardiotoxicity
Source: Nat Commun. 2026 Sep 4;17:9505. doi: 10.1038/s41467-026-77428-w (PMC13545280; doi:10.1038/s41467-026-77428-w)
Supplement: Supplementary file 1 [file 41467_2026_77428_MOESM1_ESM.pdf]

Supplementary Information

HDAC inhibition via suberoylanilide hydroxamic acid ameliorates doxorubicin-induced cardiotoxicity

Benay Eksi[1,2*], Daniel Finke[1,2*], Synje Michel[2,3], Jannek Brauer[1,2], Markus B Heckmann[1,2], Mohsen Valadan[1,2], Leonard M. Schanze[1,2], Vighnesh Sunder[1,2], Kevin Steimel[4], Jürgen Burhenne[4], Timon Seeger[1,2], Hugo A. Katus[1,2], Norbert Frey[1,2], Johannes Backs[2,3,5,6,7,8], Lorenz H. Lehmann[1,2,9, §]

[1] Department of Internal Medicine III: Cardiology, Angiology & Pulmonology, Heidelberg University Hospital, Im Neuenheimer Feld 410, 69120 Heidelberg, Germany.

[2] German Centre for Cardiovascular Research (DZHK), Partner site Heidelberg/Mannheim, 69120 Heidelberg, Germany.

[3] Heidelberg University, Medical Faculty Heidelberg, Institute of Experimental Cardiology, 69120 Heidelberg, Germany.

[4] Heidelberg University, Medical Faculty Heidelberg/Heidelberg University Hospital, Internal Medicine IX - Department of Clinical Pharmacology and Pharmacoepidemiology, 69120 Heidelberg, Germany.

[5] Heidelberg University Hospital, Department of Internal Medicine VIII, 69120 Heidelberg, Germany.

[6] Molecular Medicine Partnership Unit, European Molecular Biology Laboratory (EMBL), 69117 Heidelberg, Germany.

[7] Heidelberg University, 69120 Heidelberg, Germany.

[8] Helmholtz-Institute for Translational AngioCardioScience (HI-TAC) of the Max Delbrück Center for Molecular Medicine in the Helmholtz Association (MDC) at Heidelberg University, 69117 Heidelberg, Germany.

[9] German Cancer Research Center (DKFZ), 69120 Heidelberg, Germany.

* Authors contributed equally

[§] To whom correspondence should be addressed:

Prof. Dr. med. Lorenz Lehmann, MD

University Hospital of Heidelberg, Department of Cardiology

Im Neuenheimer Feld 410

69120 Heidelberg

Email: Lorenz.Lehmann@med.uni-heidelberg.de

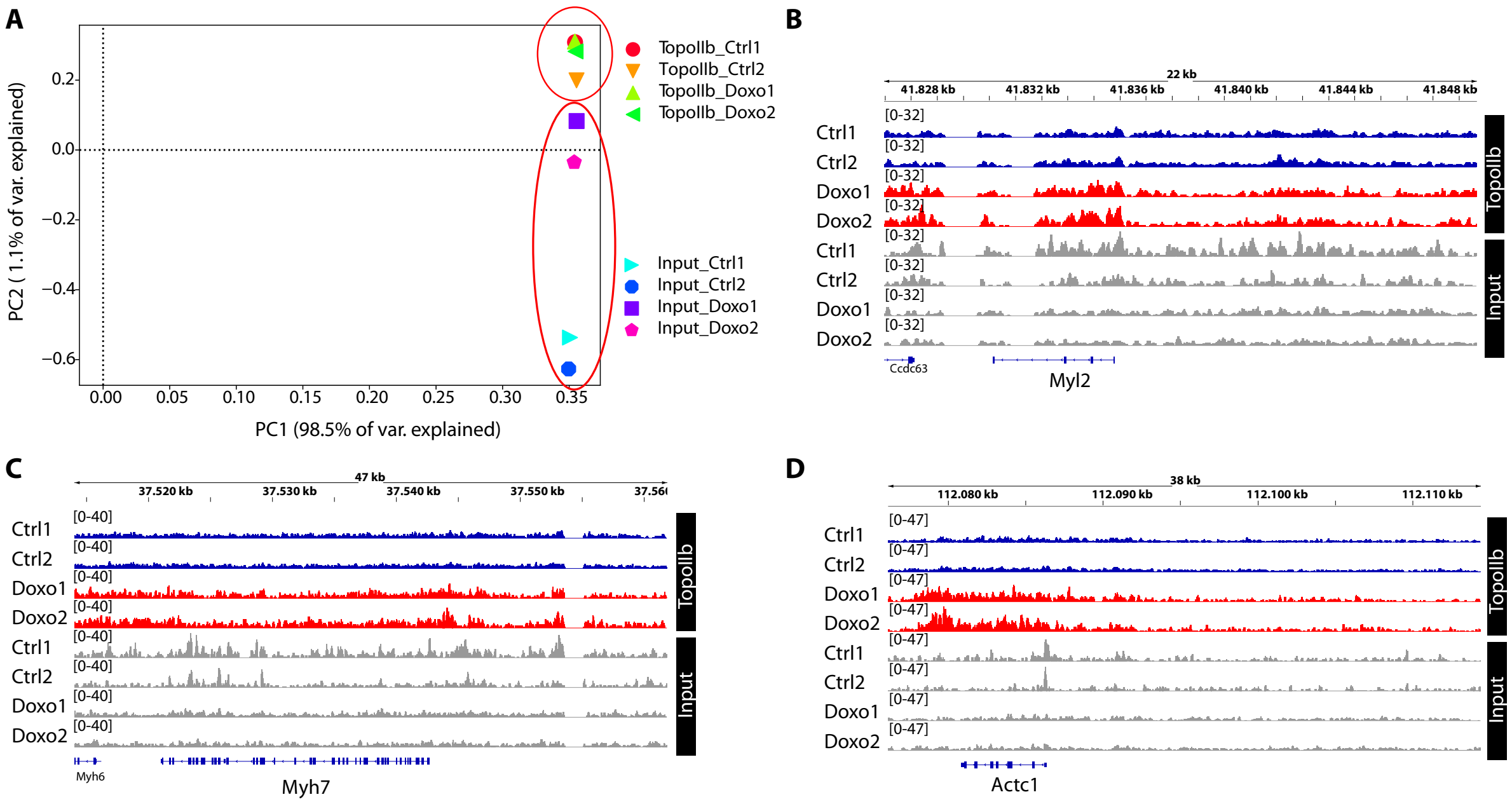

Supplementary Figure 1: (A) Principal Component Analysis (PCA) of individual chromatin immunoprecipitation sequencing (ChIP-seq) samples, including Input controls and Topoisomerase IIb (Topo IIb) pulldown.

(B-D) Single tracks of individual ChIP-seq samples and biological replicates at differentially regulated Topo IIb binding sites in the promoter regions of *Myl2, Myh7* and *Actc1*. The averaged tracks are shown in Figure 1.

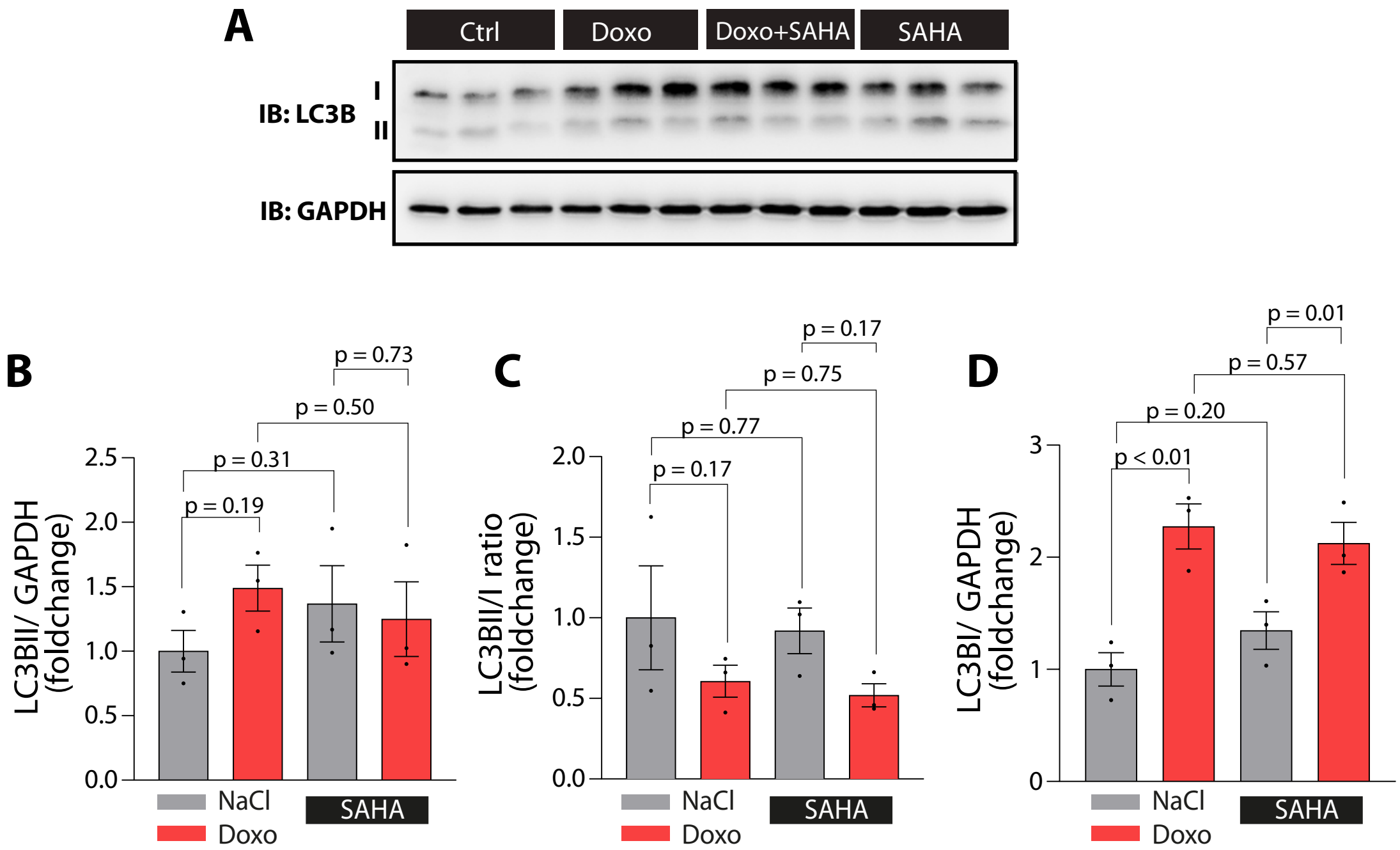

Supplementary Figure 2: (A) Immunoblots of microtubule-associated protein 1 light chain 3 beta (LC3B)-I and LC3B-II in human induced pluripotent stem cell-derived cardiomyocytes (hiPSC-CMs) treated with doxorubicin (Doxo), suberoylanilide hydroxamic acid (SAHA) or the combination. GAPDH was used as a loading control. Quantification of the WB for **(B)** LC3B II and **(C)** LC3B I each normalized to GAPDH and **(D)** quantification of the LC3BII/ I ratio. Data are presented as mean ± SEM from n = 3 per treatment group. Statistical analysis was performed using ANOVA followed by Bonferroni's multiple comparisons test; exact p-values are indicated above the comparisons. Exact p-values for the indicated comparisons in (D) were: NaCl vs. Doxo, p = 0.001; Doxo vs. Doxo + SAHA, p = 0.5667; SAHA vs. Doxo + SAHA, p = 0.0147 and NaCl vs. SAHA, p = 0.2044. Source data are provided as a Source Data file.

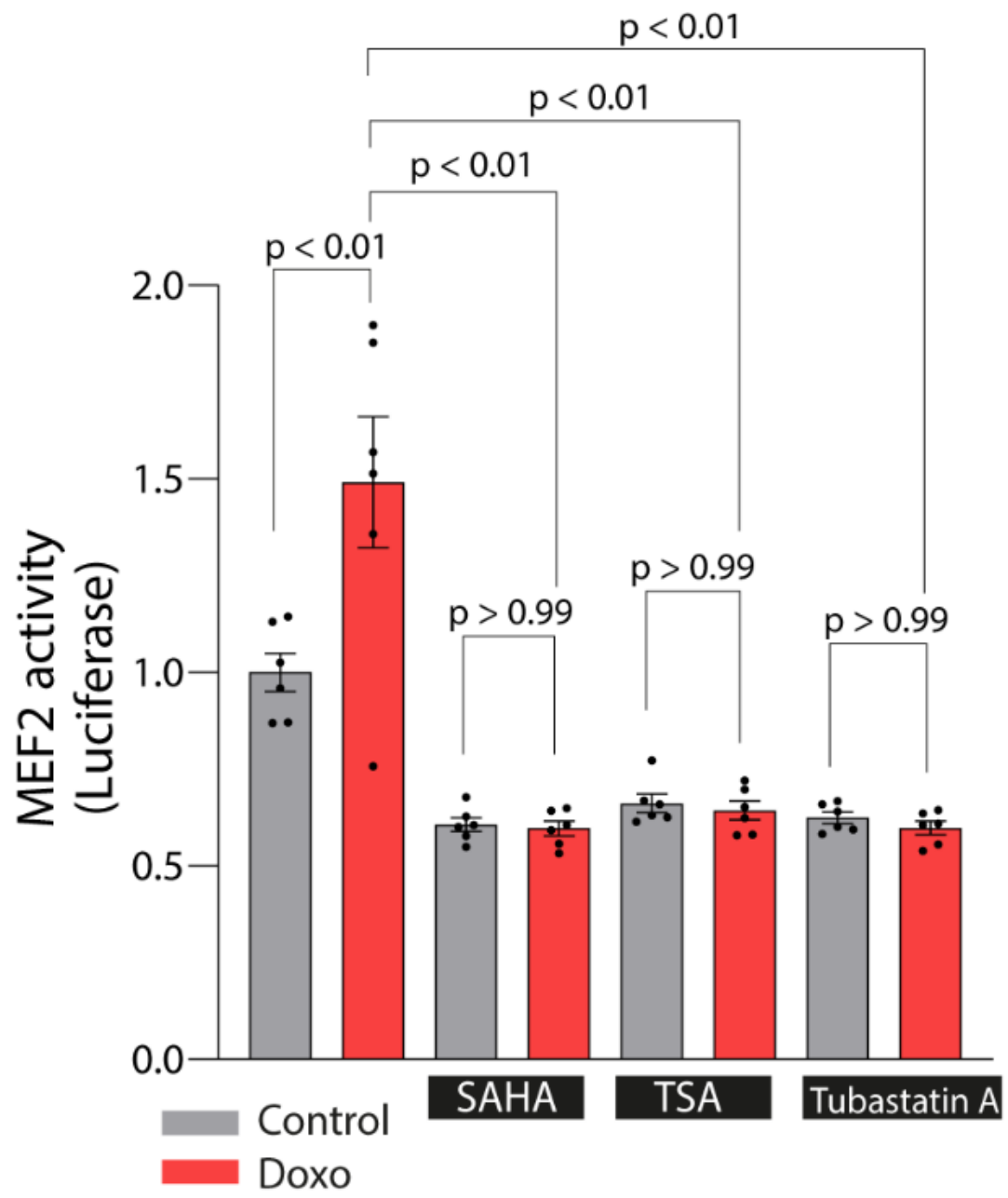

Supplementary Figure 3: Myocyte enhancer factor 2 (MEF2) luciferase reporter activity was measured in neonatal rat ventricular myocytes (NRVMs) treated with doxorubicin (Doxo) in the presence or absence of the pan-histone deacetylase inhibitors suberoylanilide hydroxamic acid (SAHA) and trichostatin A (TSA) or the selective HDAC6 inhibitor Tubastatin A. Data are presented as mean ± SEM from $n = 6$. Statistical analysis was performed using ANOVA followed by Bonferroni's multiple comparisons test. Exact p-values for the indicated comparisons were: Control vs. Doxo, $p < 0.0001$; Doxo vs. Doxo + SAHA, $p < 0.0001$; Doxo vs. Doxo + TSA, $p < 0.0001$; Doxo vs. Doxo + Tubastatin A, $p < 0.0001$; SAHA vs. Doxo + SAHA, $p > 0.9999$; TSA vs. Doxo + TSA, $p > 0.9999$; and Tubastatin A vs. Doxo + Tubastatin A, $p > 0.9999$. Source data are provided as a Source Data file.

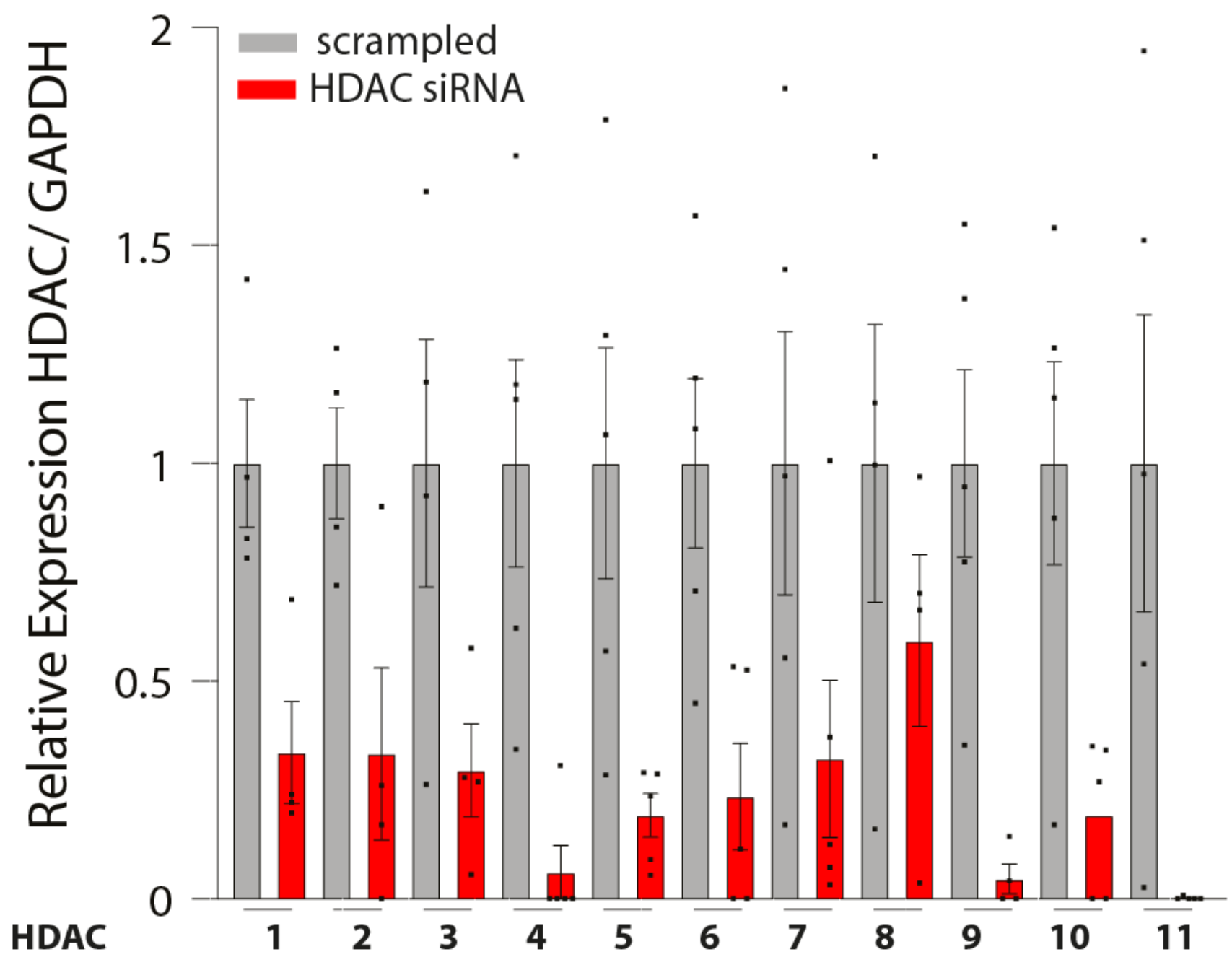

Supplementary Figure 4: Expression of HDAC1-11 on RNA level after siRNA-mediated knockdown in comparison to the respective scrambled control. Expression levels were normalized to GAPDH. Data are presented as mean ± SEM. Source data are provided as a Source Data file.

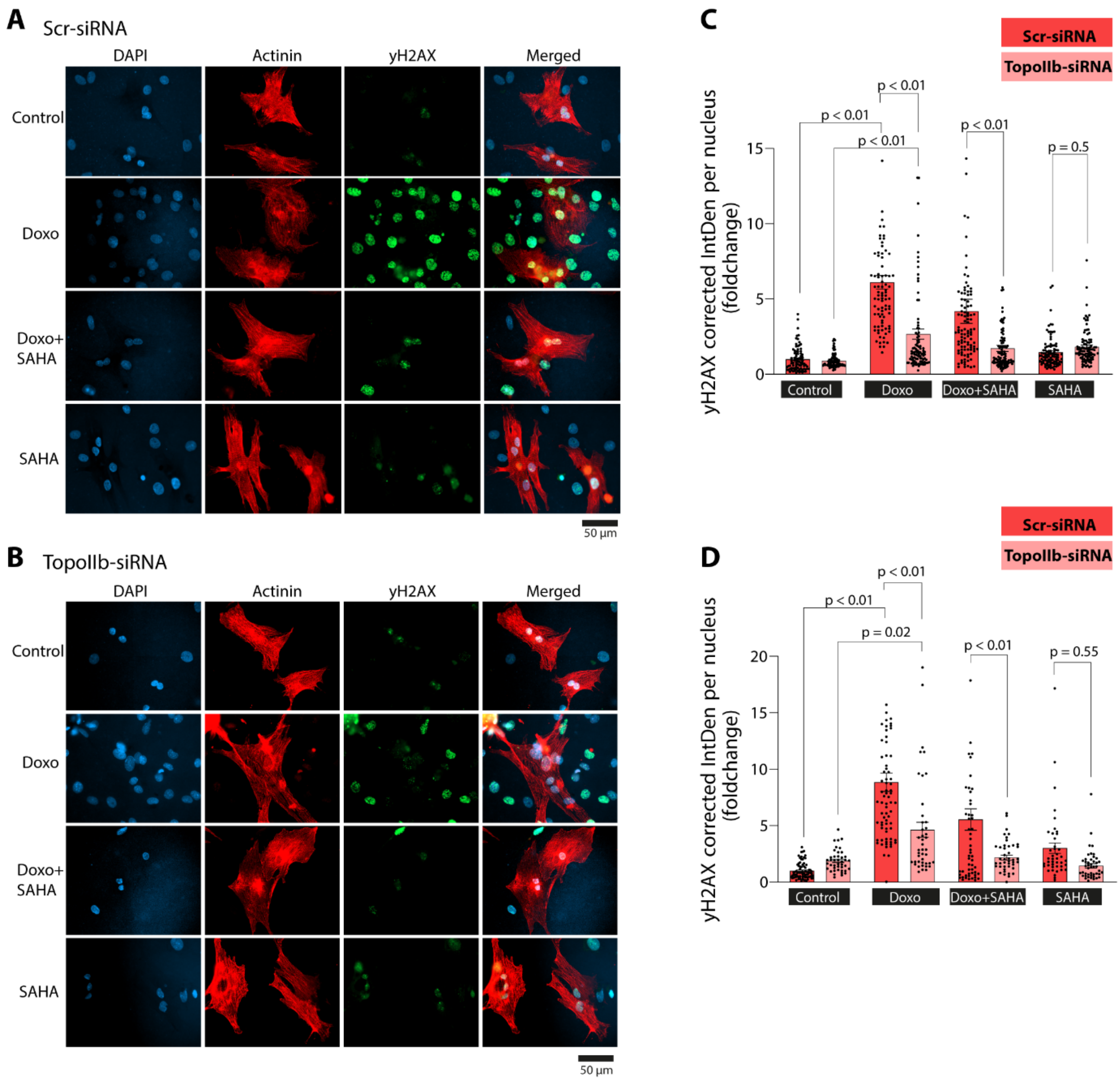

Supplementary Figure 5: Representative immunofluorescence images of phosphorylated histone H2AX (γH2AX) staining in neonatal rat ventricular myocytes (NRVMs) **(A)** transfected with scrambled (Scr-siRNA) or **(B)** Topoisomerase IIb (Topo IIb)-specific siRNA (TopoIIb-siRNA) and treated with doxorubicin (Doxo), suberoylanilide hydroxamic acid (SAHA) or the combination. Cardiomyocytes were identified by α-actinin staining and nuclei were counterstained with DAPI. Scale bar, 50 µm. Quantification of γH2AX fluorescence intensity per nucleus on **(C)** total nuclei and **(D)** in cardiomyocytes only. Each data point represents a single nucleus. A total of 10 randomly selected microscopic fields per experimental condition were analysed. Statistical analysis was performed using one-way ANOVA followed by Bonferroni's multiple comparisons test. For (C) exact p-values for the indicated comparisons were: Scr-siRNA Control vs. Scr-siRNA Doxo, $p < 0.0001$; TopoIIb-siRNA Control vs. TopoIIb-siRNA Doxo, $p = 0.0008$; Scr-siRNA Doxo vs. TopoIIb-siRNA Doxo, $p < 0.0001$; Scr-siRNA Doxo + SAHA vs. TopoIIb-siRNA Doxo + SAHA, $p < 0.0001$; and Scr-siRNA SAHA vs. TopoIIb-siRNA SAHA, $p = 0.4905$ and for (D) exact p-values for the indicated comparisons were: Scr-siRNA Doxo vs. TopoIIb-siRNA Doxo, $p < 0.0001$; Scr-siRNA Doxo + SAHA vs. TopoIIb-siRNA Doxo + SAHA, $p = 0.0006$; Scr-siRNA SAHA vs. TopoIIb-siRNA SAHA, $p = 0.5544$; Scr-siRNA Control vs. Scr-siRNA Doxo, $p < 0.0001$ and TopoIIb-siRNA Control vs. TopoIIb-siRNA Doxo, $p = 0.0246$. Source data are provided as a Source Data file.

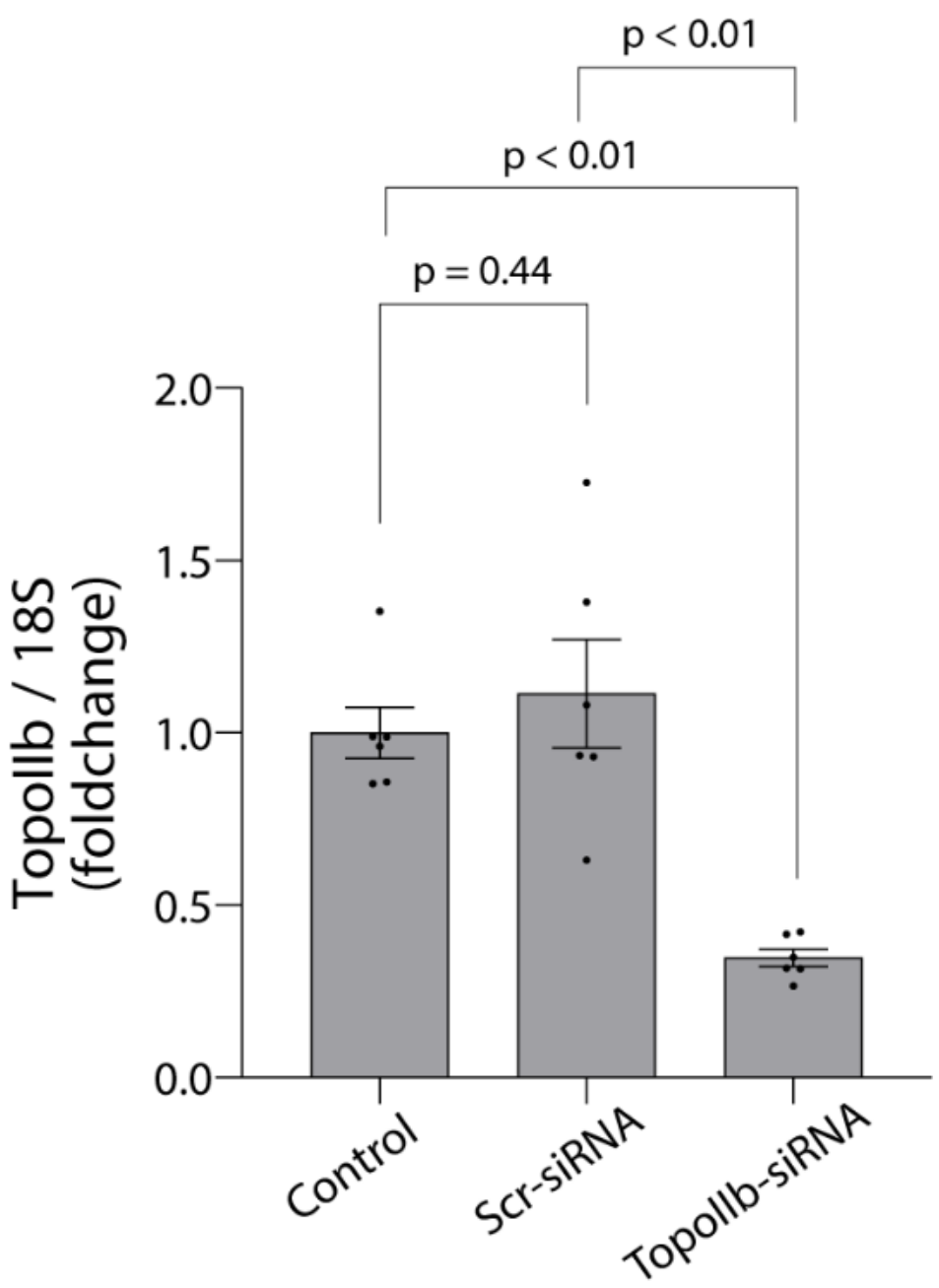

Supplementary Figure 6: Topoisomerase IIb (Topo IIb) expression levels following siRNA-mediated knockdown with scrambled *(*Scr-siRNA*)* or Topoisomerase IIb (Topo IIb)-specific siRNA *(*TopoIIb-siRNA*)* in neonatal rat ventricular myocytes (NRVMs) were normalized to 18S ribosomal RNA expression.

Data are presented as mean ± SEM from n = 6 replicates per treatment group. Statistical analysis was performed using one-way ANOVA followed by Bonferroni's multiple comparisons test; exact p-values are indicated above the comparisons. Exact p-values for the indicated comparisons were: Control vs. Scr-siRNA, $p = 0.4426$; Control vs. TopoIIb-siRNA, $p = 0.0004$; Scr-siRNA vs. TopoIIb-siRNA, $p < 0.0001$. Source data are provided as a Source Data file.

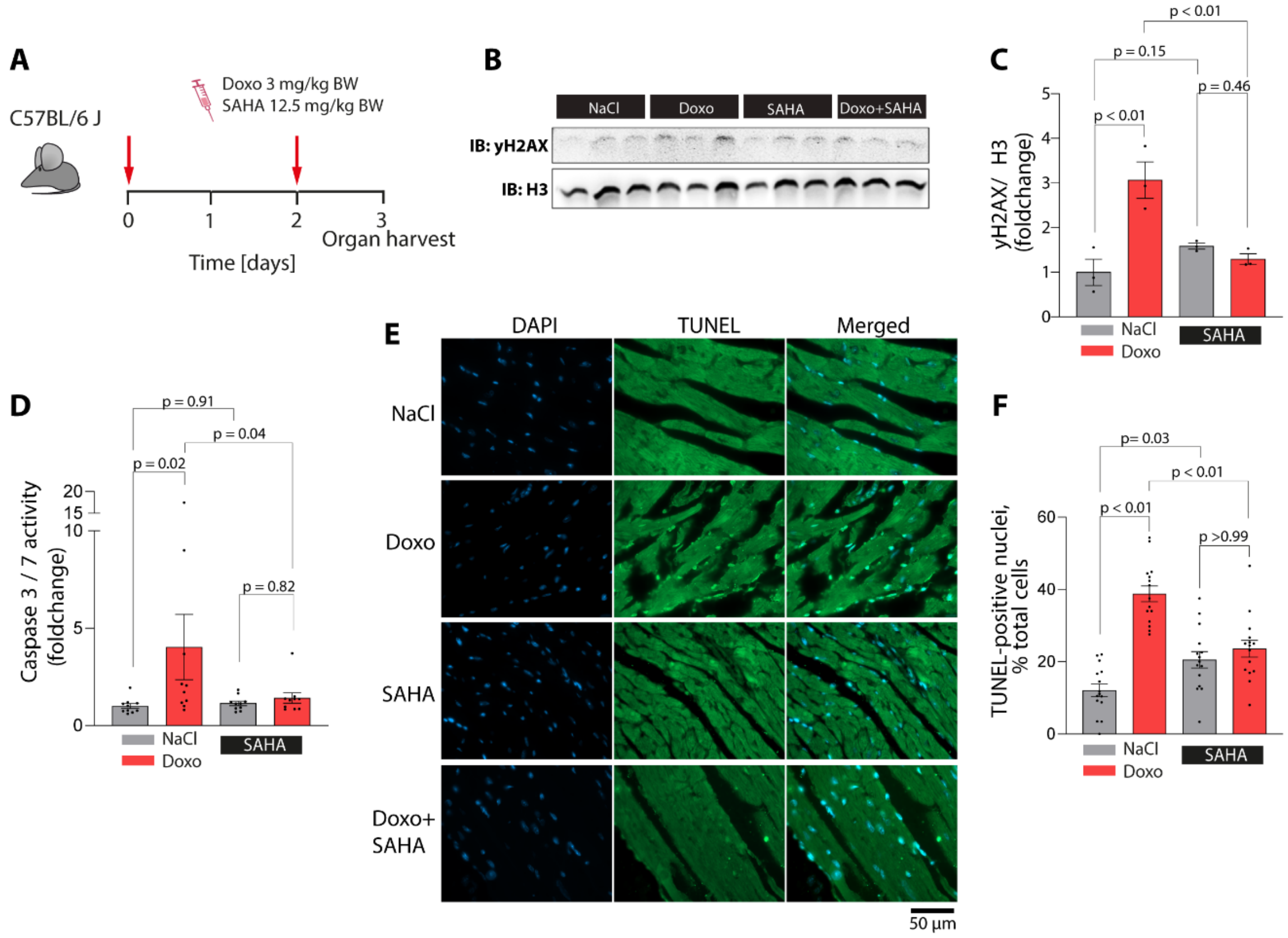

Supplementary Figure 7: (A) Experimental design of the in vivo short-term treatment protocol in C57BL/6J mice receiving doxorubicin (3 mg/kg BW) SAHA (12.5 mg/kg BW). **(B)** Representative immunoblot analysis of γH2AX expression in cardiac tissue from NaCl, Doxo ±SAHA-treated mice. Histone H3 served as loading control. **(C)** Quantification of γH2AX protein levels normalized to H3 (n=3 mice per treatment group. Data are presented as mean ± SEM. Statistical analysis was performed using one-way ANOVA followed by Bonferroni's multiple comparisons test. Exact p-values for the indicated comparisons were: NaCl vs. Doxo, p = 0.0005; NaCl vs. SAHA, p = 0.1499; Doxo vs. Doxo + SAHA, p = 0.0014; SAHA vs. Doxo + SAHA, p = 0.4569 **(D)** Caspase-3/7 activity in cardiac tissue lysates from NaCl, Doxo ±SAHA-treated mice (n= 10 mice per treatment group). Data are presented as mean ± SEM. Statistical analysis was performed using one-way ANOVA followed by Bonferroni's multiple comparisons test; exact p-values are indicated above the comparisons. **(E)** Representative images of TUNEL staining in cardiac sections obtained from NaCl, Doxo ±SAHA-treated mice. Nuclei were counterstained with DAPI. Scale bar: 50 µm. **(F)** Quantification of TUNEL staining represented as the percentage (%) of TUNEL-positive nuclei of counted total nuclei in n= 3 mice per group. For each mouse, 5 images were taken. Data are presented as mean ± SEM. Statistical analysis was performed using one-way ANOVA followed by Bonferroni's multiple comparisons test. Exact p-values for the indicated comparisons were: NaCl vs. Doxo, p < 0.0001; NaCl vs. SAHA, p = 0.0286; Doxo vs. Doxo + SAHA, p < 0.0001; SAHA vs. Doxo + SAHA, p > 0.9999. Source data are provided as a Source Data file.

```
14-3-3 beta         MTMDKSELVQKAKLAEQAERYDDMAAAMKAVTEQGHELSNEERNLLSVAY
14-3-3 gamma        -MVDREQLVQKARLAEQAERYDDMAAAMKNVTELNEPLSNEERNLLSVAY
14-3-3 zeta/delta   --MDKNELVQKAKLAEQAERYDDMAACMKSVTEQGAELSNEERNLLSVAY
14-3-3 epsilon      -MDDREDLVYQAKLAEQAERYDEMVESMKKVAGMDVELTVEERNLLSVAY
14-3-3 eta          -MGDREQLLQRARLAEQAERYDDMASAMKAVTELNEPLSNEDRNLLSVAY
14-3-3 theta        --MEKTELIQKAKLAEQAERYDDMATCMKAVTEQGAELSNEERNLLSVAY

14-3-3 beta         KNVVGARRSSWRVISSIEQKT--ERNEKKQQMGKEYREKIEAELQDICND
14-3-3 gamma         KNVVGARRSSWRVISSIEQKTSADGNEKKIEMVRAYREKIEKELEAVCQD
14-3-3 zeta/delta   KNVVGARRSSWRVVSSIEQKT--EGAEKKQQMAREYREKIETELRDICND
14-3-3 epsilon      KNVIGARRASWRIISSIEQKEENKGGEDKLKMIREYRQMVETELKLICCD
14-3-3 eta          KNVVGARRSSWRVISSIEQKTMADGNEKKLEKVKAYREKIEKELETVCND
14-3-3 theta        KNVVGGRRSAWRVISSIEQKT--DTSDKKLQLIKDYREKVESELRSICTT

14-3-3 beta         VLELLDKYLIPNAT--QPESKVFYLKMKGDYFRYLSEVASGDNKQTTVSN
14-3-3 gamma        VLSLLDNYLIKNCSETQYESKVFYLKMKGDYYRYLAEVATGEKRATVVES
14-3-3 zeta/delta   VLSLLEKFLIPNAS--QAESKVFYLKMKGDYYRYLAEVAAGDDKKGIVDQ
14-3-3 epsilon      ILDVLDKHLIPAAN--TGESKVFYYKMKGDYHRYLAEFATGNDRKEAAEN
14-3-3 eta          VLSLLDKFLIKNCNDFQYESKVFYLKMKGDYYRYLAEVASGEKKNSVVEA
14-3-3 theta        VLELLDKYLIANAT--NPESKVFYLKMKGDYFRYLAEVACGDDRKQTIDN

14-3-3 beta         SQQAYQEAFEISKKEMQPTHPIRLGLALNFSVFYYEILNSPEKACSLAKT
14-3-3 gamma        SEKAYSEAHEISKEHMQPTHPIRLGLALNYSVFYYEIQNAPEQACHLAKT
14-3-3 zeta/delta   SQQAYQEAFEISKKEMQPTHPIRLGLALNFSVFYYEILNSPEKACSLAKT
14-3-3 epsilon      SLVAYKAASDIAMTELPPTHPIRLGLALNFSVFYYEILNSPDRACRLAKA
14-3-3 eta          SEAAYKEAFEISKEQMQPTHPIRLGLALNFSVFYYEIQNAPEQACLLAKQ
14-3-3 theta        SQGAYQEAFDISKKEMQPTHPIRLGLALNFSVFYYEILNNPELACTLAKT

14-3-3 beta         AFDEAIAELDTLNEESYKDSTLIMQLLRDNLTLWTSENQGDEGDAGEGEN
14-3-3 gamma        AFDDAIAELDTLNEDSYKDSTLIMQLLRDNLTLWTSDQQDDDGGEGNN--
14-3-3 zeta/delta   AFDEAIAELDTLSEESYKDSTLIMQLLRDNLTLWTSDTQGDEAEAGEGGE
14-3-3 epsilon      AFDDAIAELDTLSEESYKDSTLIMQLLRDNLTLWTSDMQGDGEEQNKEAL
14-3-3 eta          AFDDAIAELDTLNEDSYKDSTLIMQLLRDNLTLWTSDQQDEEAGEGN---
14-3-3 theta        AFDEAIAELDTLNEDSYKDSTLIMQLLRDNLTLWTSDSAGEECDAAEGAE

14-3-3 beta         --------
14-3-3 gamma        --------
14-3-3 zeta/delta   N-------
14-3-3 epsilon      QDVEDENQ
14-3-3 eta          --------
14-3-3 theta        N-------
```

Supplementary Figure 8: Protein sequence alignment of 14-3-3 isoforms beta, gamma, zeta/delta, epsilon, eta and theta. In red squares the respective lysine residues K50, K78 and K123 are marked that were mutated to arginines.

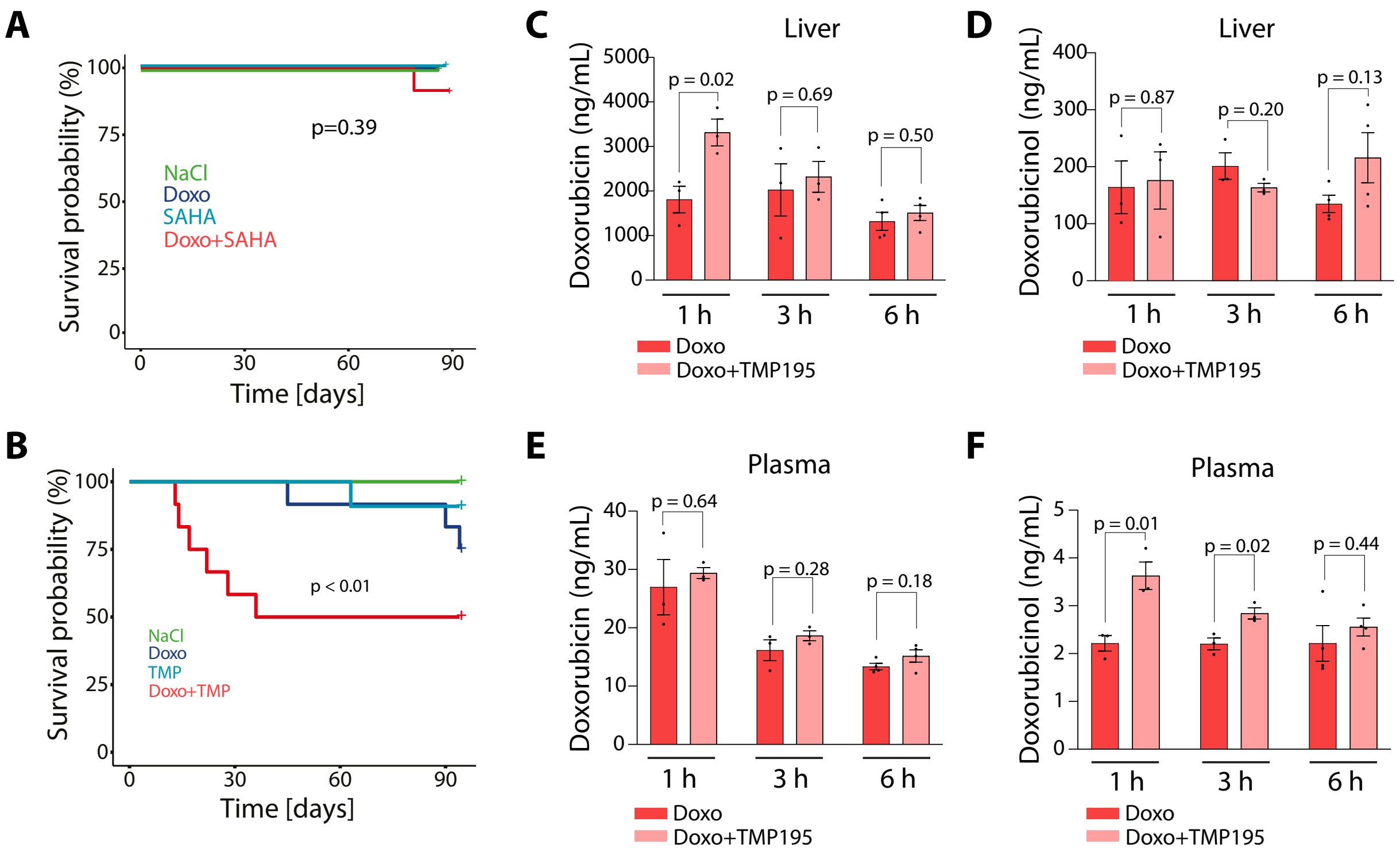

Supplementary Figure 9: Mortality analysis and effect of TMP195 on doxorubicin concentrations in liver and plasma. **(A)** Kaplan Meier curve to show survival probability (%) over time of indicated groups NaCl, Doxo, SAHA ± Doxo. P-value calculated via log-rank test. **(B)** Kaplan Meier survival analysis shows a significant decrease in survival probability in Doxo/TMP195 co-treatment (log-rank test). Doxorubicin and doxorubicinol concentrations were measured in liver tissue **(C, D)** and plasma **(E, F)** at 1, 3, and 6 h following treatment with doxorubicin (Doxo) alone or in combination with TMP195 (Doxo+TMP195). Data are presented as mean ± SEM, with individual biological replicates shown as dots. Statistical significance between groups at each time point was determined using an unpaired two-tailed Student's t-test; exact p-values are indicated above the comparisons. Source data are provided as a Source Data file.

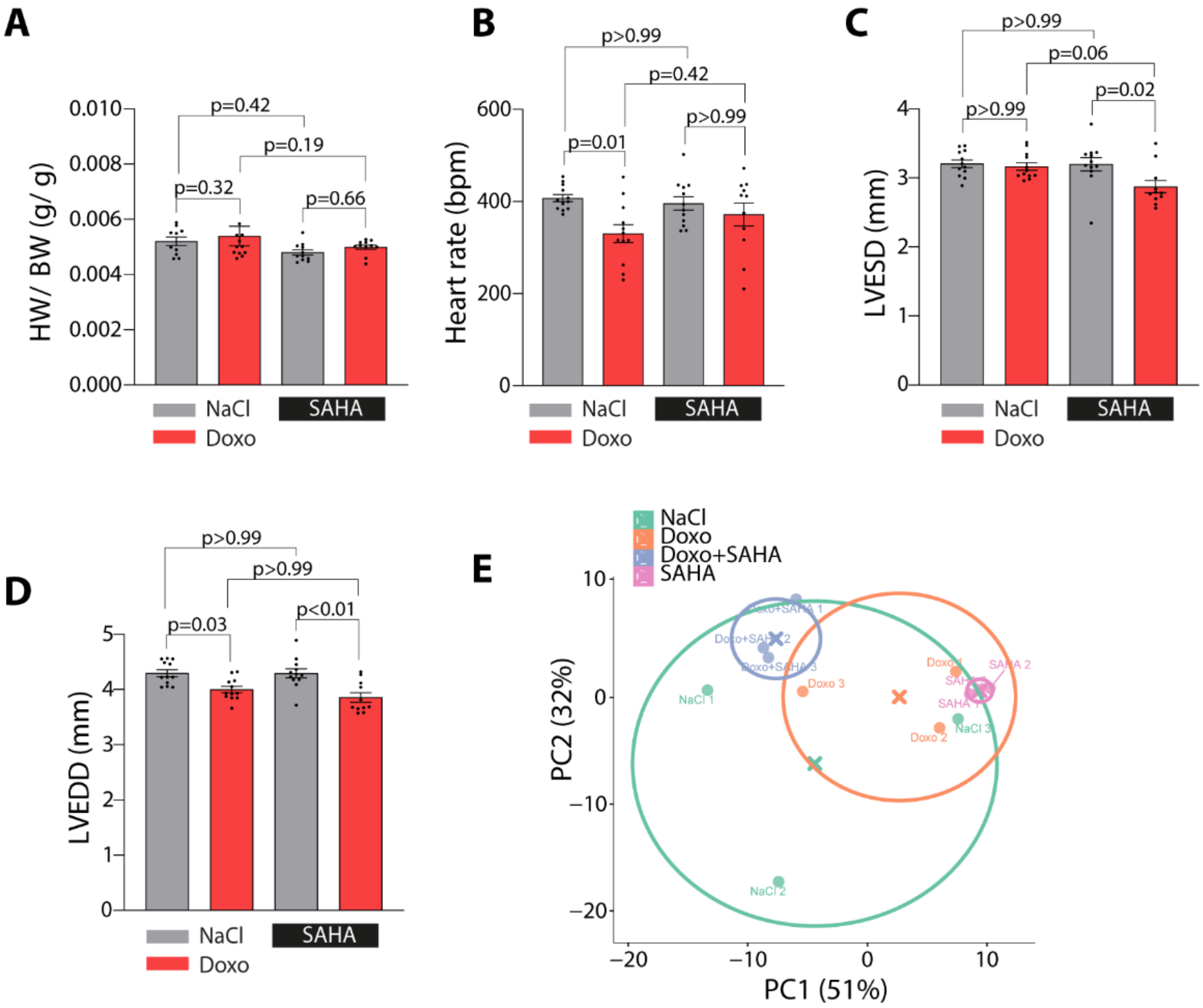

Supplementary Figure 10: **(A)** Heart weight to body weight ratio (HW/ BW) of C56BL/6J mice treated with either doxorubicin (Doxo), suberoylanilide hydroxamic acid (SAHA) or the combination. **(B)** Heart rate in beats per minute (bpm) **(C)** Left ventricular end systolic diameter (LVESD) in mm **(D)** Left ventricular end diastolic diameter (LVEDD) in mm. Data are presented as mean ± SEM from biologically independent animals (NaCl, n = 12; Doxo, n = 12; SAHA, n = 12; Doxo + SAHA, n = 11). Statistical analysis was performed using one-way ANOVA followed by Bonferroni's multiple comparisons test; exact p-values are indicated above the comparisons. Exact p-values for the indicated comparisons in (D) were: NaCl vs. Doxo, p = 0.0297; Doxo vs. Doxo + SAHA, p = 0.9998; SAHA vs. Doxo + SAHA, p = 0.0006 and NaCl vs. SAHA, p > 0.9999. (**E**) PCA of RNA-seq samples from these animals (n=3/ treatment group). Source data are provided as a Source Data file.

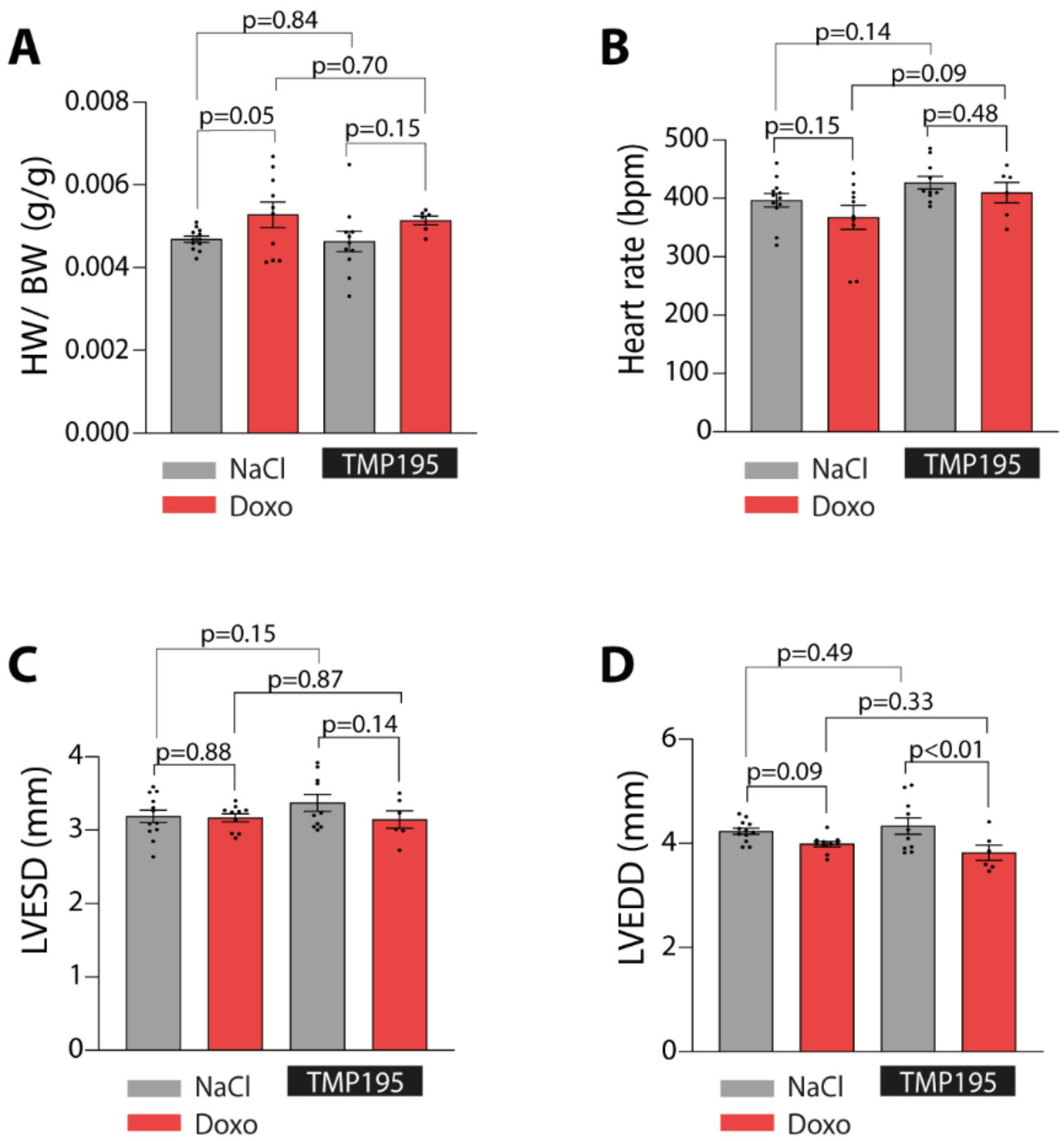

Supplementary Figure 11: **(A)** Heart weight to body weight ratio (HW/ BW) of C56BL/6J mice treated with either doxorubicin (Doxo), TMP195 or the combination. Data are presented as mean ± SEM. Statistical analysis was performed using one-way ANOVA followed by Bonferroni's multiple comparisons test; exact p-values are indicated above the comparisons. **(B)** Heart rate in beats per minute (bpm) **(C)** Left ventricular end systolic diameter (LVESD) in mm **(D)** Left ventricular end diastolic diameter (LVEDD) in mm. Data are presented as mean ± SEM from biologically independent animals (NaCl, n = 12; Doxo, n = 9; SAHA, n = 11; Doxo + SAHA, n = 6). Statistical analysis was performed using one-way ANOVA followed by Bonferroni's multiple comparisons test; exact p-values are indicated above the comparisons. Exact p-values for the indicated comparisons in (D) were: NaCl vs. Doxo, p = 0.0832; NaCl vs. TMP195, p = 0.4935; TMP195 vs. Doxo + TMP195, p = 0.0048; Doxo vs. Doxo + TMP195, p = 0.3345. Source data are provided as a Source Data file.

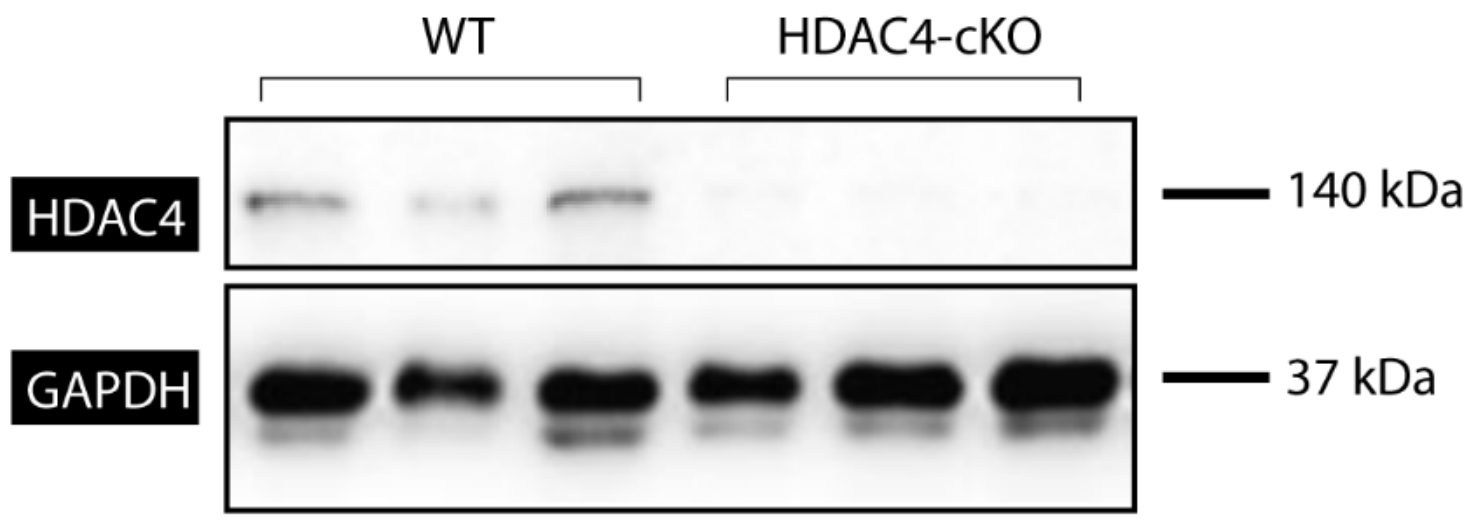

Supplementary Figure 12: Immunoblots demonstrating histone deacetylase 4 (HDAC4) protein expression in wild-type (WT) and cardiomyocyte-specific HDAC4 knockout (HDAC4-cKO) hearts. GAPDH served as a loading control. Molecular weight markers are indicated. Three representative animals per genotype are shown.

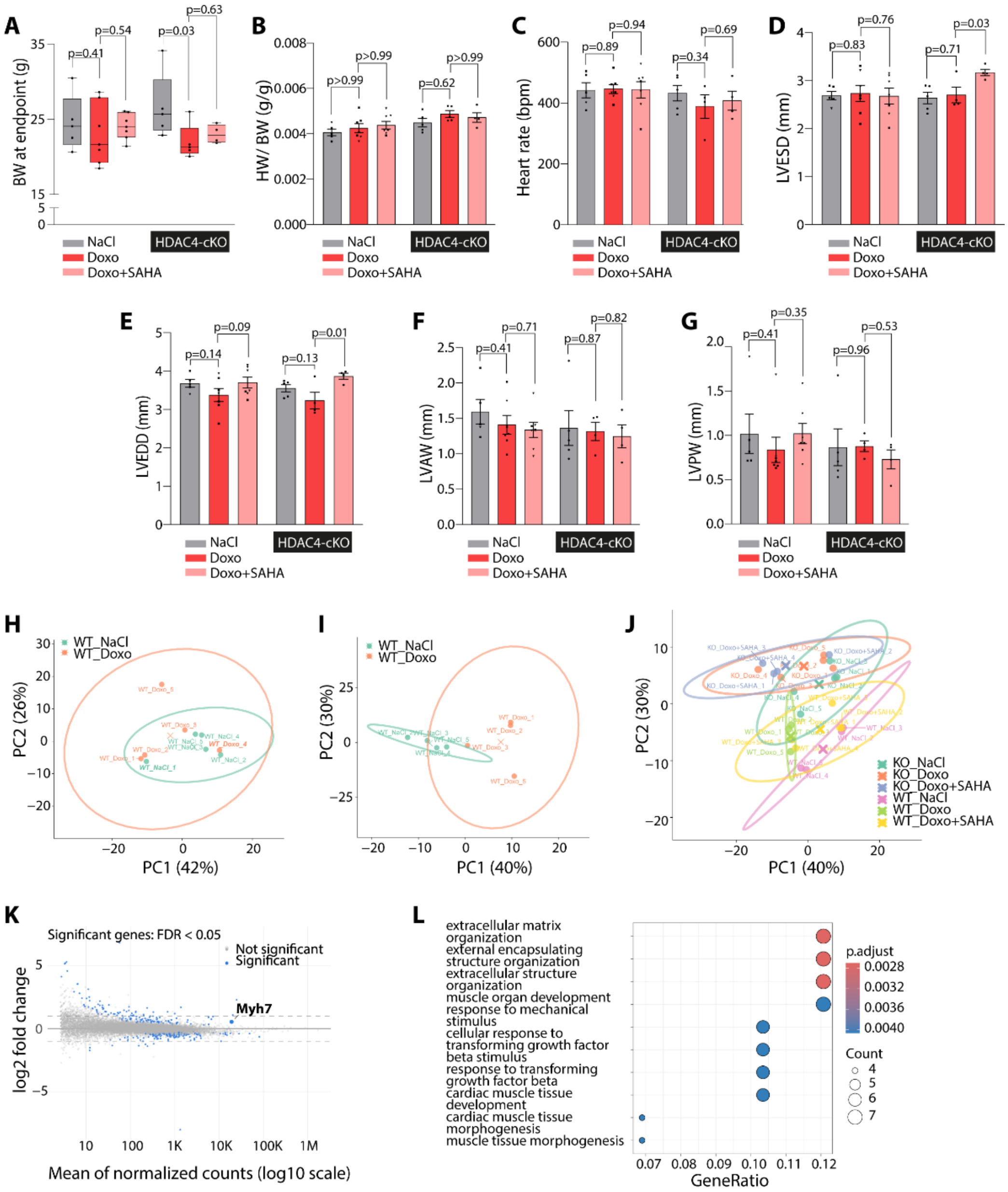

Supplementary Figure 13: **(A)** Body weights at the end of the experiment of wild-type (WT) and cardiomyocyte-specific HDAC4 knockout (HDAC4-cKO) mice treated with NaCl, doxorubicin (Doxo) or Doxo plus suberoylanilide hydroxamic acid (SAHA). **(B)** Heart weight/ body weight ratios in g/g **(C)** Heart rates in bpm. Data are presented as mean ± SEM from biologically independent animals (WT NaCl, n = 5; WT Doxo, n = 7; WT Doxo + SAHA, n = 7; HDAC4-cKO NaCl, n = 5; HDAC4-cKO Doxo, n = 5; HDAC4-cKO Doxo + SAHA, n = 4). Statistical analysis was performed using one-way ANOVA followed by Bonferroni's multiple comparisons test; exact p-values are indicated above the comparisons. **(D)** Left ventricular endsystolic diameter (LVESD) in mm **(E)** Left ventricular enddiastolic diameter (LVEDD) in mm **(F)** Left ventricular anterior wall (LVAW) in mm **(G)** Left ventricular posterior wall (LVPW) in mm. Data are presented as mean ± SEM from biologically independent animals (WT NaCl, n = 5; WT Doxo, n = 7; WT Doxo + SAHA, n = 7; HDAC4-cKO NaCl, n = 5; HDAC4-cKO Doxo, n = 4; HDAC4-cKO Doxo + SAHA, n = 4). In WT animals, squares indicate fl-/fl- cre+ whereas triangles indicate fl+/fl+ cre-. Statistical analysis was performed using one-way ANOVA followed by Bonferroni's multiple comparisons test; exact p-values are indicated above the comparisons. **(H)** PCA of WT NaCl and Doxo groups before exclusion of outliers **(I)** PCA of WT NaCl and Doxo groups after exclusion of outliers **(J)** PCA of all samples included in the analysis **(K)** DESeq2 MA Blot showing significantly regulated genes in blue, with *Myh7* highlighted **(L)** Top ten Gene Ontology terms enriched among genes significantly upregulated by Doxo and normalized by SAHA treatment in WT but not HDAC4-cKO mice. Source data are provided as a Source Data file.

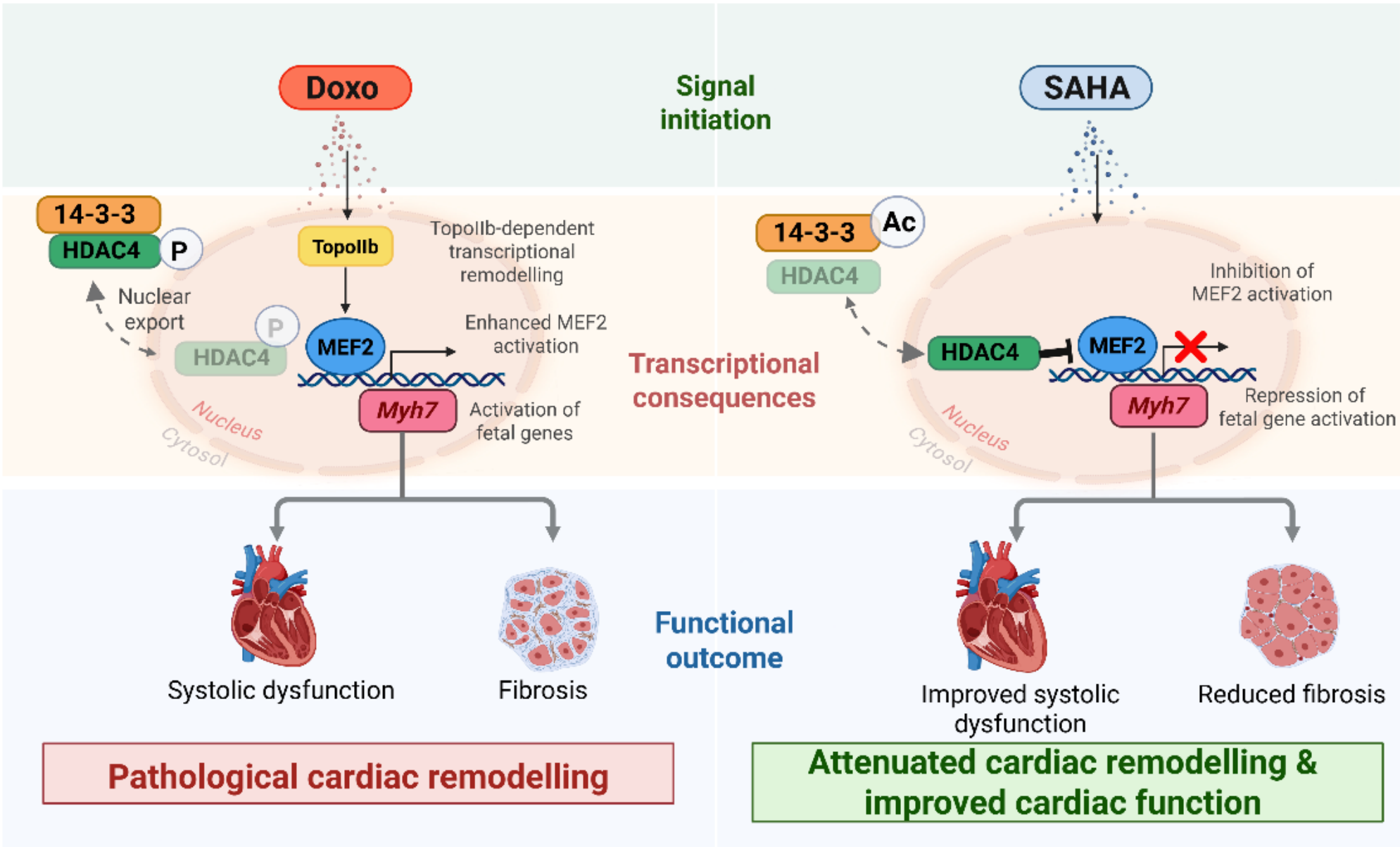

Supplementary Figure 14: Summary figure and proposed model of SAHA-mediated cardioprotection during doxorubicin-induced cardiac remodeling. Doxorubicin promotes Topo IIb-dependent transcriptional activation at MEF2-regulated cardiac genes, including *Myh7*, resulting in pathological transcriptional consequences that contribute to systolic dysfunction, fibrosis, and adverse cardiac remodeling. SAHA induces acetylation of 14-3-3, thereby disrupting 14-3-3 binding to class IIa HDACs and promoting nuclear retention of HDAC4/5. Nuclear HDAC4/5 repress MEF2-dependent transcription and attenuate doxorubicin-induced pathological gene programs. This mechanism limits fibrosis and preserves systolic function, resulting in attenuated cardiac remodeling and improved cardiac function. Ac, acetylation; Doxo, doxorubicin; HDAC, histone deacetylase; MEF2, myocyte enhancer factor 2; *Myh7*, myosin heavy chain 7; P, phosphorylation; SAHA, suberoylanilide hydroxamic acid; Topo IIb, topoisomerase IIb. The figure was created with BioRender.

Sample	Reads in peaks	Total reads	FRiP
Ctrl1 TopoIIb	768839	77471962	0,0099
Ctrl2 TopoIIb	668415	102389202	0,0065
Doxo1 TopoIIb	729805	31099212	0,0235
Doxo2 TopoIIb	389180	24472971	0,0159
Ctrl1 Input		24876067	
Ctrl2 Input		28297449	
Doxo1 Input		59160263	
Doxo2 Input		51053512	

Supplementary Table 1: List of total annotated reads (mm9), reads in called peaks (normalized to the respective input). Fraction of Reads in Peaks (FRiP) is shown for Topoisomerase (Topo IIb) ChIP samples.
