# Supplementary material for: HDAC inhibition via suberoylanilide hydroxamic acid ameliorates doxorubicin-induced cardiotoxicity
Source: Nat Commun. 2026 Sep 4;17:9505. doi: 10.1038/s41467-026-77428-w (PMC13545280; doi:10.1038/s41467-026-77428-w)
Supplement: Supplementary file 2 [file 41467_2026_77428_MOESM2_ESM.pdf]

The Description of Additional Supplementary Files

Supplementary Data 1: Annotation of ChIP-seq peaks to genomic locations and genomic features.

Supplementary Data 2: RPKM expression values from the wild-type doxorubicin/SAHA RNA-seq experiment.

Supplementary Data 3: RPKM expression values from the HDAC4-cKO doxorubicin/SAHA RNA-seq experiment.

Supplementary Data 4: DESeq2 differential expression analysis of the wild-type doxorubicin/SAHA RNA-seq experiment.

Supplementary Data 5: DESeq2 differential expression analysis of the HDAC4-cKO doxorubicin/SAHA RNA-seq experiment.
