# Supplementary material for: HDAC inhibition via suberoylanilide hydroxamic acid ameliorates doxorubicin-induced cardiotoxicity
Source: Nat Commun. 2026 Sep 4;17:9505. doi: 10.1038/s41467-026-77428-w (PMC13545280; doi:10.1038/s41467-026-77428-w)
Supplement: Supplementary file 3 [file 41467_2026_77428_MOESM8_ESM.pdf]

nature portfolio

Corresponding author(s): Prof. Dr. med. Lorenz Lehmann

Last updated by author(s): Aug 18, 2026

Reporting Summary

Nature Portfolio wishes to improve the reproducibility of the work that we publish. This form provides structure for consistency and transparency in reporting. For further information on Nature Portfolio policies, see our Editorial Policies and the Editorial Policy Checklist.

Statistics

For all statistical analyses, confirm that the following items are present in the figure legend, table legend, main text, or Methods section.

n/a	Confirmed	
☐	☒	The exact sample size (*n*) for each experimental group/condition, given as a discrete number and unit of measurement
☐	☒	A statement on whether measurements were taken from distinct samples or whether the same sample was measured repeatedly
☐	☒	The statistical test(s) used AND whether they are one- or two-sided<br>*Only common tests should be described solely by name; describe more complex techniques in the Methods section.*
☒	☐	A description of all covariates tested
☐	☒	A description of any assumptions or corrections, such as tests of normality and adjustment for multiple comparisons
☐	☒	A full description of the statistical parameters including central tendency (e.g. means) or other basic estimates (e.g. regression coefficient) AND variation (e.g. standard deviation) or associated estimates of uncertainty (e.g. confidence intervals)
☐	☒	For null hypothesis testing, the test statistic (e.g. *F*, *t*, *r*) with confidence intervals, effect sizes, degrees of freedom and *P* value noted<br>*Give P values as exact values whenever suitable.*
☒	☐	For Bayesian analysis, information on the choice of priors and Markov chain Monte Carlo settings
☒	☐	For hierarchical and complex designs, identification of the appropriate level for tests and full reporting of outcomes
☒	☐	Estimates of effect sizes (e.g. Cohen's *d*, Pearson's *r*), indicating how they were calculated

Our web collection on statistics for biologists contains articles on many of the points above.

Software and code

Policy information about availability of computer code

Data collection	Echocardiography: VisualSonics Vevo 2100 (MX550D); LVEF measured in Vevo Lab v5.7.1. Histology images: Zeiss Axio Scan.Z1 slide scanner (brightfield). Immunofluorescence images: Axio Observer Z1 fluorescence microscope. Luciferase: Perkin Elmer microplate reader. ChIP-seq: Illumina HiSeq 2000 (100 bp SE; library QC by Agilent Bioanalyzer); ChIP performed using Diagenode Bioruptor and IP-Star. Bulk RNA-seq: BGI on DNBSEQ™ platform (100 bp PE; ~20M reads/sample); RNA quantified on NanoDrop (ThermoFisher); qPCR: Applied Biosystems ViiA™ 7 Real-Time PCR System, Drug quantification: Drug quantification: ACQUITY UPLC system (Binary Solvent Manager and Sample Manager) coupled to a Xevo TQ-S triple quadrupole mass spectrometer (Waters); data acquisition and quantification using MassLynx v4.2.; data acquisition/quantification using [software, version]. Caspase-3/7 assay: VICTOR Nivo multimode microplate reader (PerkinElmer); TUNEL assay: Leica fluorescence microscope.
Data analysis	Statistics: GraphPad Prism v10.4.2 (and Prism v10.4.1 for some ChIP-seq plots). Histology/Immunofluorescence quantification: ImageJ v1.54. ChIP-seq: FASTQ aligned to rat genome rn5 using bowtie2 v2.3.4.1; peak calling MACS2 v2.1.1; differential peaks DESeq2 v1.24.0; heatmaps/profiles deepTools v3.2.1; visualization IGV v2.13.1; PCA multiBamSummary and plotPCA v3.5.4; peak annotation HOMER v4.10 (annotatePeaks.pl); track generation bamCoverage, bigwigCompare and averaging bigwigAverage (deepTools v3.2.1). RNA-seq: FASTQ aligned to mouse genome mm9 using bowtie2 version 2.3.4.1; counts via featureCounts (Rsubread); normalization/DE/PCA via DESeq2 v1.40.2; heatmaps via heatmap3 v1.42.1. GO enrichment analysis using clusterProfiler v4.8.3 (BP, MF, CC; Benjamini–Hochberg correction; adjusted p ≤ 0.01, q ≤ 0.05), with enrichment visualization using DOSE package (version 3.26.2). External hiPSC RNA-seq (GSE106297): re-analysed using normalized count matrices (FPKM) with alignment to hg38 for gene annotation.

For manuscripts utilizing custom algorithms or software that are central to the research but not yet described in published literature, software must be made available to editors and reviewers. We strongly encourage code deposition in a community repository (e.g. GitHub). See the Nature Portfolio guidelines for submitting code & software for further information.

Data

Policy information about availability of data

All manuscripts must include a data availability statement. This statement should provide the following information, where applicable:

- Accession codes, unique identifiers, or web links for publicly available datasets
- A description of any restrictions on data availability
- For clinical datasets or third party data, please ensure that the statement adheres to our policy

Raw and annotated Topo IIb ChIP-seq data are deposited at EMBL-EBI: E-MTAB-16326. Published MEF2 ChIP-seq used for overlap analysis: E-MTAB-6213. RNA-seq data are deposited under E-MTAB-16361. Re-analyzed hiPSC cardiomyocyte RNA-seq data were obtained from GEO GSE106297.

Research involving human participants, their data, or biological material

Policy information about studies with human participants or human data. See also policy information about sex, gender (identity/presentation), and sexual orientation and race, ethnicity and racism.

Reporting on sex and gender	*Use the terms sex (biological attribute) and gender (shaped by social and cultural circumstances) carefully in order to avoid confusing both terms. Indicate if findings apply to only one sex or gender; describe whether sex and gender were considered in study design; whether sex and/or gender was determined based on self-reporting or assigned and methods used. Provide in the source data disaggregated sex and gender data, where this information has been collected, and if consent has been obtained for sharing of individual-level data; provide overall numbers in this Reporting Summary. Please state if this information has not been collected. Report sex- and gender-based analyses where performed, justify reasons for lack of sex- and gender-based analysis.*
Reporting on race, ethnicity, or other socially relevant groupings	*Please specify the socially constructed or socially relevant categorization variable(s) used in your manuscript and explain why they were used. Please note that such variables should not be used as proxies for other socially constructed/relevant variables (for example, race or ethnicity should not be used as a proxy for socioeconomic status). Provide clear definitions of the relevant terms used, how they were provided (by the participants/respondents, the researchers, or third parties), and the method(s) used to classify people into the different categories (e.g. self-report, census or administrative data, social media data, etc.) Please provide details about how you controlled for confounding variables in your analyses.*
Population characteristics	*Describe the covariate-relevant population characteristics of the human research participants (e.g. age, genotypic information, past and current diagnosis and treatment categories). If you filled out the behavioural & social sciences study design questions and have nothing to add here, write "See above."*
Recruitment	*Describe how participants were recruited. Outline any potential self-selection bias or other biases that may be present and how these are likely to impact results.*
Ethics oversight	*Identify the organization(s) that approved the study protocol.*

Note that full information on the approval of the study protocol must also be provided in the manuscript.

Field-specific reporting

Please select the one below that is the best fit for your research. If you are not sure, read the appropriate sections before making your selection.

☒ Life sciences ☐ Behavioural & social sciences ☐ Ecological, evolutionary & environmental sciences

For a reference copy of the document with all sections, see nature.com/documents/nr-reporting-summary-flat.pdf

Life sciences study design

All studies must disclose on these points even when the disclosure is negative.

Sample size	No sample size calculation was performed. Sample sizes were chosen empirically based on established doxorubicin cardiotoxicity protocols and expected variability of echocardiographic endpoints, while minimizing animal use. The sample sizes were sufficient to detect statistically significant differences across multiple independent readouts (echocardiography, fibrosis quantification, qPCR and sequencing).
Data exclusions	RNA-seq (HDAC4-cKO experiment): two outliers were excluded upon PCA quality check (stated in Results/Supplementary Figure 13). Other pre-established exclusion criteria were not described. Animal models: Final n for each analysis corresponds to the number of animals measured at that timepoint and is reported in figure/legend.
Replication	In vitro luciferase assays (NRVMs / COS cells/ hiPSC): multiple independent experiments were performed (2-3 independent experiments), with multiple measurements per group (e.g. 9-18 measurements/group depending on assay) as reported in figure legends (MEF2 reporter; HDAC-14-3-3 binding assays). Immunofluorescence (HDAC4 localization): quantification was performed blinded with >10 fields of view recorded and group sizes for image-based quantification are provided in the figure legends. Immunofluorescence (γH2AX staining): quantification was performed with blinded analysis of >10 randomly selected fields of view. Caspase assay: n=10 mice /group. TUNEL assay: n=3 mice/group with 5 fields of view selected each. ChIP-seq: Topo IIb ChIP-seq used two biological replicates per treatment/condition. Bulk

RNA-seq: biological replication was performed with 3 animals/group for WT Doxo±SAHA and 5 animals/group for HDAC4-cKO and littermate controls. External human dataset validation: re-analysis of published hiPSC-cardiomyocyte RNA-seq with 2 sets of duplicates per condition (n=4). In vivo studies-Long-term:findings were assessed in independent mouse models (WT Doxo±SAHA (n=12/group); WT Doxo±TMP195 (n=12/group); HDAC4-cKO ± SAHA (n=5-8/group)) with individual endpoint group sizes stated in figure. In vivo studies-Short-term: WT Doxo ±SAHA (n=5/group), independently repeated twice. Histology quantification reproducibility: fibrosis quantification used a fixed threshold across images, one section per mouse and 5 randomly selected ROIs per section supporting within-sample measurement consistency.

Randomization

Animals were randomly assigned to treatment groups. Randomization was performed while balancing sex and age as evenly as possible across groups. For histology, five ROIs per section were selected randomly for quantification.

Blinding

Animals/samples were coded by numbers and investigators were blinded to treatment/genotype during data collection and analysis (including echocardiography and histology). Group codes were revealed after completion of analysis. HDAC4 localization immunofluorescence quantification, TUNEL assay quantification and γH2AX quantification was performed blinded.

Reporting for specific materials, systems and methods

We require information from authors about some types of materials, experimental systems and methods used in many studies. Here, indicate whether each material, system or method listed is relevant to your study. If you are not sure if a list item applies to your research, read the appropriate section before selecting a response.

Materials & experimental systems

n/a	Involved in the study
☐	☒ Antibodies
☐	☒ Eukaryotic cell lines
☒	☐ Palaeontology and archaeology
☐	☒ Animals and other organisms
☒	☐ Clinical data
☒	☐ Dual use research of concern
☒	☐ Plants

Methods

n/a	Involved in the study
☐	☒ ChIP-seq
☒	☐ Flow cytometry
☒	☐ MRI-based neuroimaging

Antibodies

Antibodies used

Primary antibodies:
ANTI-FLAG® M2 Affinity Gel, Sigma-Aldrich, A2220, clone M2, mouse monoclonal IgG1, agarose conjugate, IP
anti-FLAG, Sigma-Aldrich, F3165, clone M2, mouse monoclonal, dilution 1:1000 for WB, dilution 1:200 for IF
anti-myc (Myc-Tag 71D10, Cell Signaling Technology, 2278S, rabbit mAb)
anti-14-3-3 (EPR6380, Abcam, ab125032, rabbit mAB), dilution 1:1000
anti-HDAC4 (Abcam, ab12172, rabbit pAB, lot number: 1064148-9), dilution 1:1000 for WB
anti-GAPDH (14C10, Cell Signaling Technology, 2118S, rabbit mAb), dilution 1:1000 for WB
anti-Topoisomerase IIb (Topo2b, A-12, Santa Cruz, sc-365071, mouse mAB), 3.5 µg antibody per 3.0 µg chromatin
anti-α-actinin (Clone EA-53, Sigma-Aldrich / MilliporeSigma, A7811, mouse mAB), dilution 1:200
Pan-acetyl lysine agarose beads: Immune Chem ICP0388, Lot: #092512, 40 µl per 1 mg protein
anti-Phospho-Histone H2A.X (Ser139) (Cell Signaling Technology, 2577S, rabbit pAb), dilution 1:1000 for WB; dilution 1:200 for IF
anti-LC3B (Cell Signaling Technology, 2775S, rabbit pAb), dilution 1:1000 for WB
anti-H3 (Abcam, ab70550, rabbit pAb), dilution 1:1000 for WB

Secondary antibodies:
Alexa Fluor 594 goat anti-mouse (abcam, ab150116, pAB), dilution 1:400 for IF
Alexa Fluor 488 goat anti-rabbit (ThermoFisher, A-11008, pAB), dilution 1:400 for IF
anti-mouse IgG HRP-linked (Cell Signaling Technology, #7076, horse pAb), dilution 1:2500 for WB
anti-rabbit IgG-HRP-linked, (Santa Cruz Biotechnology, sc-2357, mouse pAB), dilution 1:2500 for WB

Validation

Primary antibodies:
ANTI-FLAG® M2 Affinity Gel: all species expressing FLAG-tagged proteins, validated by the manufacturer for immunoprecipitation and purification of FLAG-tagged fusion proteins. The monoclonal M2 antibody specifically binds the FLAG epitope and is covalently conjugated to agarose (https://www.sigmaaldrich.com).
anti-FLAG: species reactivity with all - Validated for IP, WB, ICC/IF. The monoclonal M2 antibody recognizes the FLAG epitope at N-terminal, Met-N-terminal, C-terminal and internal positions of FLAG fusion proteins (validated by manufactor, https://www.sigmaaldrich.com)
anti-myc: species reactivity expected with all - Validated for WB, IP, IF, Flow (validated by manufactor, https://www.cellsignal.com)
anti-14-3-3: species reactivity with human, mouse, rat - Validated for WB (human). Abcam validates it for western blotting and provides representative blots showing a band near the expected ~28 kDa size, https://www.abcam.com
anti-HDAC4: species reactivity with human, mouse, rat - Validated for WB (validated by manufactor and in HDAC4-cKO mouse model used in our experiment in Supplementary Figure 12)
anti-GAPDH: species reactivity with human, mouse, rat, monkey, bovine, pig - Validated/used for WB, IHC, IF and flow cytometry (validated by manufactor, https://www.cellsignal.com)
anti-Topoisomerase IIb: species reactivity with mouse, rat, human - Validated for WB, IP, IF, IHC, ELISA (validated by manufactor,

April 2023

https://www.scbt.com)
anti-α-actinin: species reactivity with mouse, human, frog, pig, feline & more- Validated for WB, IF/ICC, IHC, ELISA (validated by manufactor, https://www.sigmaaldrich.com)
anti-Phospho-Histone H2A.X (Ser139): species reactivity with human, mouse, rat, monkey - validated for WB, IF and flow cytometry (validated by manufactor, https://www.cellsignal.com)
anti-LC3B: species reactivity with human, mouse, rat- Validated for WB (validated by manufactor, https://www.cellsignal.com)
anti-H3: species reactivity with mouse and human; predicted to react with rat and several other species. Validated by the manufacturer for WB, IP, ICC/IF and IHC. The manufacturer demonstrates detection of Histone H3 at the expected molecular weight in western blotting and specific nuclear staining in ICC/IF, https://www.abcam.com.

Eukaryotic cell lines

Policy information about cell lines and Sex and Gender in Research

Cell line source(s)

COS-7 cells (in-house stock, ATCC, CRL-1651)
Primary cardiomyocytes: NRVMs isolated from 1-2 day old Sprague Dawley rats (primary culture)
Human induced pluripotent stem cell (hiPSC) line SCVI15, derived from male donor, was generated from peripheral blood mononuclear cells through Sendai virus-mediated episomal reprogramming by the Stanford Cardiovascular Institute Biobank

Authentication

Cell line was not formally authenticated.

Mycoplasma contamination

The cell lines were not tested for mycoplasma contamination.

Commonly misidentified lines
(See ICLAC register)

no misidentified lines used

Animals and other research organisms

Policy information about studies involving animals; ARRIVE guidelines recommended for reporting animal research, and Sex and Gender in Research

Laboratory animals

Mouse (Mus musculus). Wild-type: C57BL/6J, 10-week-old mice purchased from Janvier Labs. Cardiomyocyte-specific HDAC4-cKO mice on a C57BL/6N background were generated by crossing HDAC4-floxed mice with α-MHC-MerCreMer mice as previously described (ref. 21) and bred at the Heidelberg University animal facility (IBF). Experiments were performed in 10-13-week-old mice; recombination was induced by tamoxifen. Mice were housed under specific pathogen-free (SPF) conditions at 22 ± 2 °C and 50–60% relative humidity under a 12 h light/12 h dark cycle, with food and water available ad libitum.

Wild animals

Study did not involve wild animals.

Reporting on sex

Long-term experiments included both male and female mice. Wild-type cohorts initially comprised 12 mice per group (6 females and 6 males). HDAC4-cKO experiments included both sexes, with exact numbers of females and males stated in the corresponding figure legends. Sex was recorded but analyses were not stratified by sex because the study was not powered for sex-specific comparisons. Short-term experiments were performed in male mice only.

Field-collected samples

Study did not involve samples collected from the field.

Ethics oversight

All animal experiments were reviewed and approved by the Animal Experiment review board of Baden-Württemberg, Germany.

Note that full information on the approval of the study protocol must also be provided in the manuscript.

Plants

Seed stocks

Report on the source of all seed stocks or other plant material used. If applicable, state the seed stock centre and catalogue number. If plant specimens were collected from the field, describe the collection location, date and sampling procedures.

Novel plant genotypes

Describe the methods by which all novel plant genotypes were produced. This includes those generated by transgenic approaches, gene editing, chemical/radiation-based mutagenesis and hybridization. For transgenic lines, describe the transformation method, the number of independent lines analyzed and the generation upon which experiments were performed. For gene-edited lines, describe the editor used, the endogenous sequence targeted for editing, the targeting guide RNA sequence (if applicable) and how the editor was applied.

Authentication

Describe any authentication procedures for each seed stock used or novel genotype generated. Describe any experiments used to assess the effect of a mutation and, where applicable, how potential secondary effects (e.g. second site T-DNA insertions, mosiacism, off-target gene editing) were examined.

ChIP-seq

Data deposition

☒ Confirm that both raw and final processed data have been deposited in a public database such as GEO.

☐ Confirm that you have deposited or provided access to graph files (e.g. BED files) for the called peaks.

Data access links<br>*May remain private before publication.*	https://www.ebi.ac.uk/biostudies/ArrayExpress/studies/E-MTAB-16326?key=22bfe984-52bf-4f24-8187-14320db2ab71 (E-MTAB-16326)
Files in database submission	raw and annotated Topo IIb ChIP-seq (E-MTAB-16326, private -> see reviewer link above); MEF2 ChIP-seq used for overlap (E-MTAB-6213, public dataset)
Genome browser session<br>(e.g. UCSC)	*Provide a link to an anonymized genome browser session for "Initial submission" and "Revised version" documents only, to enable peer review. Write "no longer applicable" for "Final submission" documents.*

Methodology

Replicates	Topo IIb ChIP-seq was performed with two biological replicates per condition (Ctrl and doxorubicin-treated NRVMs).
Sequencing depth	100 bp single-end; four samples on one lane
Antibodies	anti-Topoisomerase IIb (Topo2b, A-12, Santa Cruz, sc-365071, mouse mAB)
Peak calling parameters	Peak calling: MACS2 v2.1.1, p=0.0001, input-normalized. Differential enrichment: DESeq2 v1.24.0, cutoff FDR < 0.1.
Data quality	Annotated peak numbers and FRiP scores (Supplementary Table 1); PCA performed via multiBamSummary
Software	bowtie2 v2.3.4.1; MACS2 v2.1.1; DESeq2 v1.24.0; deepTools v3.2.1; IGV v2.13.1; HOMER v4.10; multiBamSummary v3.5.4; bamCoverage; bigwigAverage; bigwigCompare
