# Supplementary material for: HDAC inhibition via suberoylanilide hydroxamic acid ameliorates doxorubicin-induced cardiotoxicity
Source: Nat Commun. 2026 Sep 4;17:9505. doi: 10.1038/s41467-026-77428-w (PMC13545280; doi:10.1038/s41467-026-77428-w)
Supplement: Supplementary file 4 [file 41467_2026_77428_MOESM9_ESM.pdf]

nature portfolio

Peer Review File

HDAC inhibition via suberoylanilide hydroxamic acid (SAHA) ameliorates Doxorubicin-induced cardiotoxicity

Corresponding Author: Professor Lorenz Lehmann

This file contains all reviewer reports in order by version, followed by all author rebuttals in order by version.

Version 0:

Reviewer comments:

Reviewer #1

(Remarks to the Author)
In this manuscript, Eksi and colleagues demonstrate a role for HDAC4-mediated regulation of MEF2 and its downstream transcriptional targets in doxorubicin (Dox) cardiotoxicity. These data extend previous reports that topoisomerase 2b (Top2b) is an mediator of Dox cardiotoxicity and show that Dox enhances Top2b accumulation at specific cardiomyocyte gene promoters. This is an interesting and important observation that advances the field in connecting Top2b to downstream mechanisms of cardiac injury. The use of suberoylanilide hydroxamic acid (SAHA), an approved pan-HDAC inhibitor that is already used for the treatment of cancer, increases the likelihood of translation and suggests that cardioprotection could be conferred by HDAc inhibitors in a way that also maximizes antitumor activity. Overall the investigators have performed a series of innovative and well-designed experiments, and the findings will be impactful to the field. The manuscript could be further strengthened as follows:

Major comments:
1. Methods/Results: The investigators explore in detail the upstream regulation of key genes regulated by the HDAC4/MEF2 pathway (including Myh7), but it is not clear how these transcriptional changes result in the classic downstream features of Dox cardiotoxicity. It would be useful to show if markers of the following are changed in SAHA treatment: DNA damage, oxidative stress, intracellular iron overload, and autophagy. These additional data would more convincingly link HDAC-regulated transcription to the end phenotypes of Dox cardiotoxicity. Although fibrosis is specifically implicated, it does not fully explain all the phenotypes described, which include change in LV function and cardiac atrophy.

2. Discussion: In the in vivo SAHA and HDAC4-cKO studies, Myh7 is noted to be upregulated by Dox, but it is not shown experimentally that this upregulation results directly from modulation of HDAC/MEF2. Given that there are other transcriptional regulators of Myh7, this should be noted as a limitation in the Discussion.

Minor comments:
1. In addition to listing the citation in the Methods, it would be helpful to include the citation to the publicly available RNAseq dataset used in lines 369-370 within the Results section.

2. Were doxorubicin and SAHA administered via IP or IV injection?

Reviewer #2

(Remarks to the Author)
This study proposes a Topoisomerase IIβ–HDAC4–MEF2 axis linking doxorubicin-induced DNA damage to maladaptive transcriptional remodeling in cardiomyocytes, and suggests that the pan-HDAC inhibitor SAHA may offer cardioprotection in this setting. The integration of genomic profiling, molecular assays, and in vivo mouse models is a strength, and the cardiomyocyte-specific HDAC4 knockout provides supportive genetic evidence for pathway involvement. The identification of 14-3-3 acetylation as a regulator of class II HDAC localization is also intriguing. The idea of repurposing SAHA as a cardioprotective adjunct during anthracycline therapy is appealing; however, all in vivo experiments use SAHA prophylactically, which limits clinical appeal. Demonstrating benefit in a treatment (rather than prevention) paradigm would substantially strengthen translational relevance. Overall, much of the current evidence remains associative, the in vivo effects are modest, and key mechanistic steps are not directly tested. Substantially deeper mechanistic validation and

stronger human relevance will be needed to support the proposed pathway and its translational implications.

Major Comments

• In Fig. 3F–I, LVEF is the most compelling functional readout, but overall cardioprotective effects of SAHA appear modest. In Fig. 3G–I, interpretation of heart rate, LVESD, and LVEDD is challenging: reduced heart rate does not necessarily indicate cardiotoxicity, and ventricular dilation would typically be expected but is not consistently observed. Consider moving panels 3G–I to the supplement.

• Similarly, antifibrotic effects appear subtle in Fig. 3L, and Fig. 3M shows substantial variability in the control+dox+SAHA group.

• All in vivo studies administer SAHA concurrently with doxorubicin. It is unclear whether SAHA can reverse established doxorubicin-induced injury. Testing delayed SAHA administration after cardiotoxicity onset would substantially improve translational relevance, as universal prophylactic use may be difficult to implement clinically.

• While TopoIIβ ChIP-seq shows increased enrichment at MEF2-bound promoters following doxorubicin, functional dependence of MEF2 activation on TopoIIβ is not directly tested. The data currently support association rather than causality. This could be strengthened by TopoIIβ depletion or inhibition followed by assessment of MEF2 activity and endogenous target gene expression, as well as temporal analyses demonstrating that TopoIIβ binding precedes transcriptional activation. In addition, locus-specific validation using CUT&RUN or CUT&Tag (e.g., for TopoIIβ, MEF2, and γH2AX) at representative promoters would help establish whether TopoIIβ recruitment coincides with local DNA damage and MEF2 engagement at the same genomic sites.

• SAHA is a broad-spectrum HDAC inhibitor with multiple histone and non-histone targets. Although HDAC4 dependence is demonstrated genetically in vivo, parallel or off-target mechanisms remain possible. This concern is underscored by HDAC6 knockdown also suppressing MEF2 activity and by the broad effects of SAHA on 14-3-3 acetylation. Additional specificity controls would help, such as comparison with another pan-HDAC inhibitor (e.g., trichostatin A), inclusion of HDAC6-selective inhibition (e.g., tubastatin A), or testing whether SAHA directly alters TopoIIβ chromatin binding.

• The mechanistic framework around 14-3-3 acetylation remains incomplete. While lysine 50 acetylation is implicated in regulating class II HDAC localization, upstream acetyltransferase(s) are not identified. Partial pathway resolution—for example, testing p300/CBP involvement pharmacologically or genetically, or assessing whether doxorubicin alone induces partial 14-3-3 acetylation—would strengthen this section. Rescue experiments using acetylation-deficient versus acetyl-mimetic 14-3-3 mutants would also help establish whether this modification is necessary and sufficient for HDAC4 nuclear retention and MEF2 repression.

• Human relevance remains limited. The re-analysis of published hiPSC-derived cardiomyocyte RNA-seq lacks sufficient contextual detail, and key mechanistic features (MEF2 activation, TopoIIβ promoter binding, HDAC4 localization, 14-3-3 acetylation, SAHA rescue) are not directly examined in human cardiomyocytes.

• Translational confidence would be improved by targeted validation in human cardiomyocytes or integration of existing human datasets (e.g., bulk or single-cell cardiac RNA-seq from doxorubicin-exposed samples) to assess whether the transcriptional programs identified in mice are similarly engaged in human disease.

Minor Comments

– Provide brief contextual information on hiPSC-derived cardiomyocytes (e.g., healthy background, general maturation state).
– Methods line 131: TMP195 is introduced without description; please clarify its target and purpose.
– Clarify rationale for use of rat cardiomyocytes in some experiments and mouse models in others.
– Figure 1C: improve color contrast.
– Line 372: define FPKM at first use.
– Line 380: clarify whether experiments were performed in mouse or rat systems.
– Indicate whether sex-dependent differences were assessed.
– Clarify whether SAHA-only treatment groups were consistently included across transcriptional analyses.

Reviewer #3

(Remarks to the Author)
I co-reviewed this manuscript with one of the reviewers who provided the listed reports. This is part of the Nature Communications initiative to facilitate training in peer review and to provide appropriate recognition for Early Career Researchers who co-review manuscripts.

Reviewer #4

(Remarks to the Author)
The manuscript by Eksi and colleagues reveals that anthracycline-induced cardiotoxicity is driven in part by Topoisomerase

IIβ (Topo IIβ) accumulation at cardiomyocyte gene promoters, where doxorubicin enhances MEF2-dependent transcription. This pathological response is attenuated by the pan-HDAC inhibitor SAHA. Mechanistically, SAHA promotes acetylation of 14-3-3, disrupts its interaction with HDAC4/5, facilitates their nuclear accumulation, represses MEF2-driven transcription, and ultimately mitigates doxorubicin-induced cardiotoxicity in vivo. Overall, the study highlights an epigenetic mechanism underlying anthracycline cardiotoxicity and suggests that HDAC inhibition has promising translational value as a cardioprotective strategy for patients receiving anthracycline therapy.

Major

1. Introduction section: the third paragraph of the Introduction is hard to follow for readers that are not experts in the field of HDAC signaling, consider rephrasing.

2. Material and Methods section: be more precise on describing the sex of mouse models used. For obvious reasons, anthracycline use for breast cancer is more prevalent in female patients so that a consideration of gender is important in this setting. Further, it was noticed that wild-type animals used were c57BL6J, while the HDAC4 KO mice are on a c57BL6N background. Did the authors notice differences in cardiac function between the genetic strains?

3. Results: how does a concentration of 1 µM - 2.5 µM doxorubicin correlate with dosages used in patients? In Fig1H-J there seems no evidence of a dose dependent increase, was this expected?

4. The selective knockdown of HDAC1-11 in Figure 2 was an elegant approach to decipher which HDAC isoform is involved in doxorubicin/SAHA responsiveness. Panel E misses size bars and the immunofuorence images seem out of focus, however, while the blots in panels K and L could use a bit more background (less contrast).

5. The differences in panels A and B (Figure 3) in cardiac hypertrophy response are confusing. Is this accounted for by a loss of body weight in response to doxorubicin? Regarding the changes in the rest of the parameters in the study (<LVEF, < LVEDD, < LVPW), what type of remodeling are we observing? Is this a classical HFrEF response? Did the authors record indices of diastolic function? Panel L and M in Figure 3 and 4, respectively, seem to be out of focus.

6. Anthracyclin-induced “cardiomyopathy” is classically accompannied by high levels of apoptosis, possibly this could be aan additional readout parameter for the studies.

7. The failure in HDAC4 KO to respond to SAHA is very strong evidence for the involvement of this isoform in the signaling underlying anthracyclin-induced cardiac toxicity, making Figure 5 an essential part of the strength of this study. One criticism pertains to the resolution of panel F.

8. The Discussion section should be double-checked for spelling errors.

Version 1:

Reviewer comments:

Reviewer #1

(Remarks to the Author)
The authors have done an excellent job with this revision. I have only one minor comment:

The new data presented in Supplementary Figure 8 are very helpful. However, in panel E, it appears as though there is more TUNEL staining in Doxo + SAHA mice compared to Doxo alone. This is likely because of differences in cardiomyocyte density between the various panels (including the NaCl panel as well). It would be helpful to show images that are more representative of the summary data presented in panel F.

Reviewer #2

(Remarks to the Author)
I thank the authors for their careful revision. The manuscript has been substantially strengthened through the addition of new mechanistic experiments, additional in vivo data, improved presentation of the functional studies, and a more balanced discussion of the study's limitations. The new TopoIIβ loss-of-function experiments and the additional DNA damage and apoptosis data strengthen the proposed mechanism and improve the overall rigor of the study.

I also appreciate that the authors addressed concerns regarding the presentation of the echocardiographic and fibrosis data by moving less informative panels to the supplement and tempering the interpretation of the in vivo findings.

While not every requested experiment was performed (particularly delayed SAHA treatment), the authors provide a reasonable rationale for focusing on concurrent administration, which aligns with their proposed clinical application. I no longer consider this essential for publication.

Overall, I believe the manuscript has been substantially improved, and I have only a few minor comments.

Minor comments
• Throughout the Discussion, I encourage the authors to continue distinguishing between findings that directly demonstrate causality and those that remain inferential.
• The rationale for focusing on concurrent SAHA administration rather than delayed treatment is reasonable. I suggest emphasizing this point more explicitly in the Discussion, as it helps frame the intended translational application and addresses an important potential criticism.

Reviewer #3

(Remarks to the Author)
I co-reviewed this manuscript with one of the reviewers who provided the listed reports. This is part of the Nature Communications initiative to facilitate training in peer review and to provide appropriate recognition for Early Career Researchers who co-review manuscripts.

Reviewer #4

(Remarks to the Author)
no further comments

Open Access This Peer Review File is licensed under a Creative Commons Attribution 4.0 International License, which permits use, sharing, adaptation, distribution and reproduction in any medium or format, as long as you give appropriate credit to the original author(s) and the source, provide a link to the Creative Commons license, and indicate if changes were made.
In cases where reviewers are anonymous, credit should be given to 'Anonymous Referee' and the source.
The images or other third party material in this Peer Review File are included in the article's Creative Commons license, unless indicated otherwise in a credit line to the material. If material is not included in the article's Creative Commons license and your intended use is not permitted by statutory regulation or exceeds the permitted use, you will need to obtain permission directly from the copyright holder.
To view a copy of this license, visit https://creativecommons.org/licenses/by/4.0/

Reviewer 1

Reviewer #1 (Remarks to the Author):

In this manuscript, Eksi and colleagues demonstrate a role for HDAC4-mediated regulation of MEF2 and its downstream transcriptional targets in doxorubicin (Dox) cardiotoxicity. These data extend previous reports that topoisomerase 2b (Top2b) is an mediator of Dox cardiotoxicity and show that Dox enhances Top2b accumulation at specific cardiomyocyte gene promoters. This is an interesting and important observation that advances the field in connecting Top2b to downstream mechanisms of cardiac injury. The use of suberoylanilide hydroxamic acid (SAHA), an approved pan-HDAC inhibitor that is already used for the treatment of cancer, increases the likelihood of translation and suggests that cardioprotection could be conferred by HDAc inhibitors in a way that also maximizes antitumor activity. Overall the investigators have performed a series of innovative and well-designed experiments, and the findings will be impactful to the field. The manuscript could be further strengthened as follows:

We thank the reviewer for the positive comments. We are happy to revise the work in order to further strengthen the manuscript.

Major comments:

1. Methods/Results: The investigators explore in detail the upstream regulation of key genes regulated by the HDAC4/MEF2 pathway (including Myh7), but it is not clear how these transcriptional changes result in the classic downstream features of Dox cardiotoxicity. It would be useful to show if markers of the following are changed in SAHA treatment: DNA damage, oxidative stress, intracellular iron overload, and autophagy. These additional data would more convincingly link HDAC-regulated transcription to the end phenotypes of Dox cardiotoxicity. Although fibrosis is specifically implicated, it does not fully explain all the phenotypes described, which include change in LV function and cardiac atrophy.

We thank the reviewer for this comment. Indeed, we were focused on long-term effects and have now added additional readouts for doxorubicin toxicity from a new short-term experiment. To account for double strand breaks, we analyzed γH2ax in a new experiment of short-term treated mice (Supplementary Figure 7A). The results showed increased γH2ax after Doxo treatment which was attenuated by co-treatment with SAHA (Supplementary Figure 7B-C). As an additional readout for apoptosis, we added Caspase Assays showing increased Caspase 3/7 activity after Doxo treatment which was attenuated with SAHA co-treatment (Supplementary Figure 7D)

We further quantified TUNEL positive cells and found and increase after doxorubicin treatment which was attenuated in SAHA co-treatment. (Supplementary Figure 7E-F).

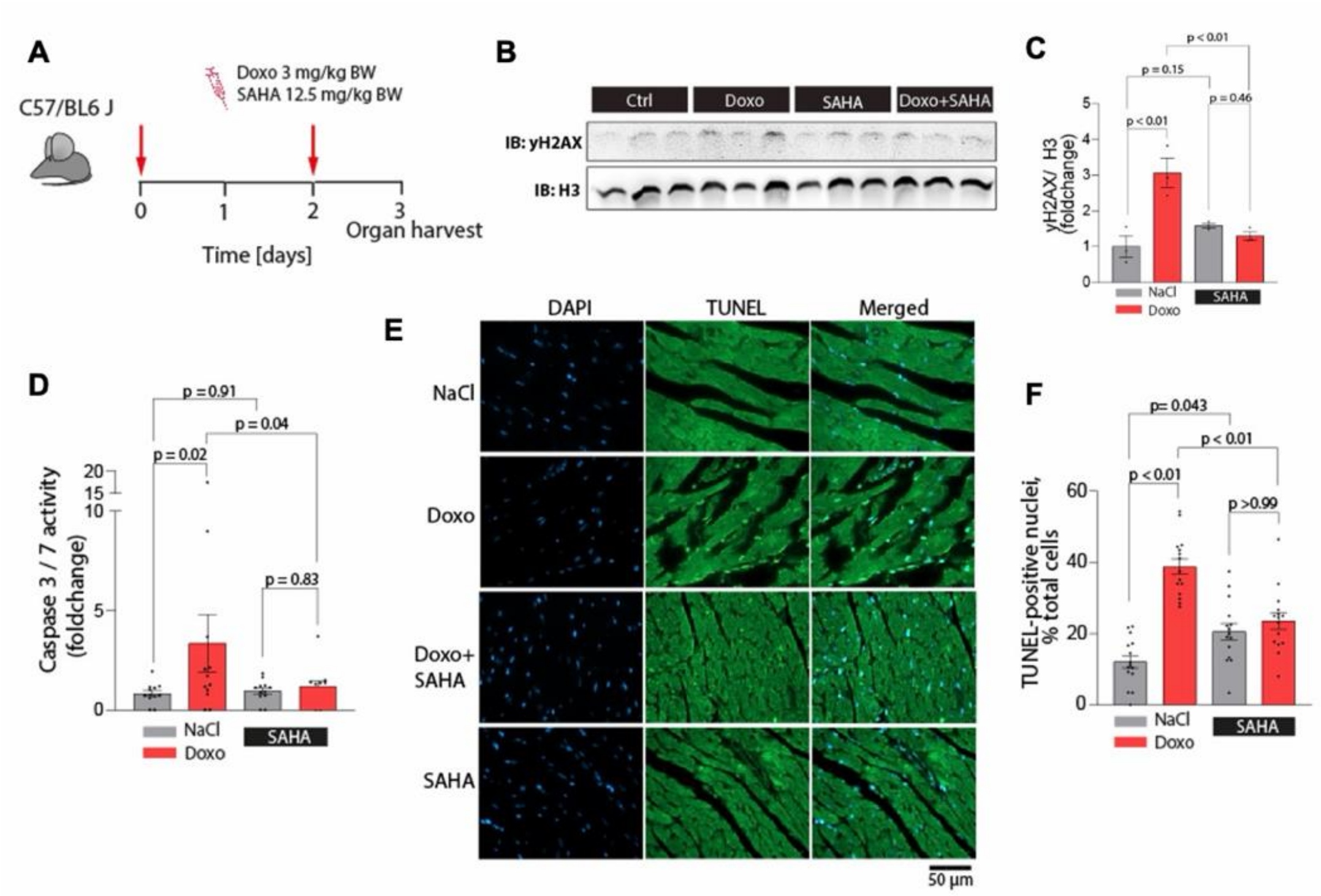

Supplementary Figure 8: SAHA attenuates doxorubicin-induced DNA damage and apoptosis *in vivo*. (**A**) Experimental design of the *in vivo* short-term treatment protocol in C57BL/6J mice receiving doxorubicin (3 mg/kg BW) SAHA (12.5 mg/kg BW). (**B**) Representative immunoblot analysis of γH2AX expression in cardiac tissue from NaCl, Doxo ±SAHA-treated mice. Histone H3 served as loading control. (**C**) Quantification of γH2AX protein levels normalized to H3. (**D**) Caspase-3/7 activity in cardiac tissue lysates from NaCl, Doxo ±SAHA-treated mice (n= 10 mice per treatment group). (**E**) Representative images of TUNEL staining in cardiac sections obtained from NaCl, Doxo ±SAHA-treated mice. Nuclei were counterstained with DAPI. Scale bar: 50 µm. (**F**) Quantification of TUNEL staining represented as the percentage (%) of TUNEL positive nuclei of counted total nuclei in n= 3 mice per group. For each mouse 10 images were taken. For statistical test ANOVA with Bonferroni correction was used.

To further assess alternative pathways for cardiotoxicity, we measured the autophagy marker LC3B in hiPSCs treated with doxorubicin, SAHA or combination of both. We found higher LC3B expression after Doxo treatment and similar levels in the presence of SAHA (Supplementary Figure 2), which points towards less relevance for the observed phenotype in the animal experiments.

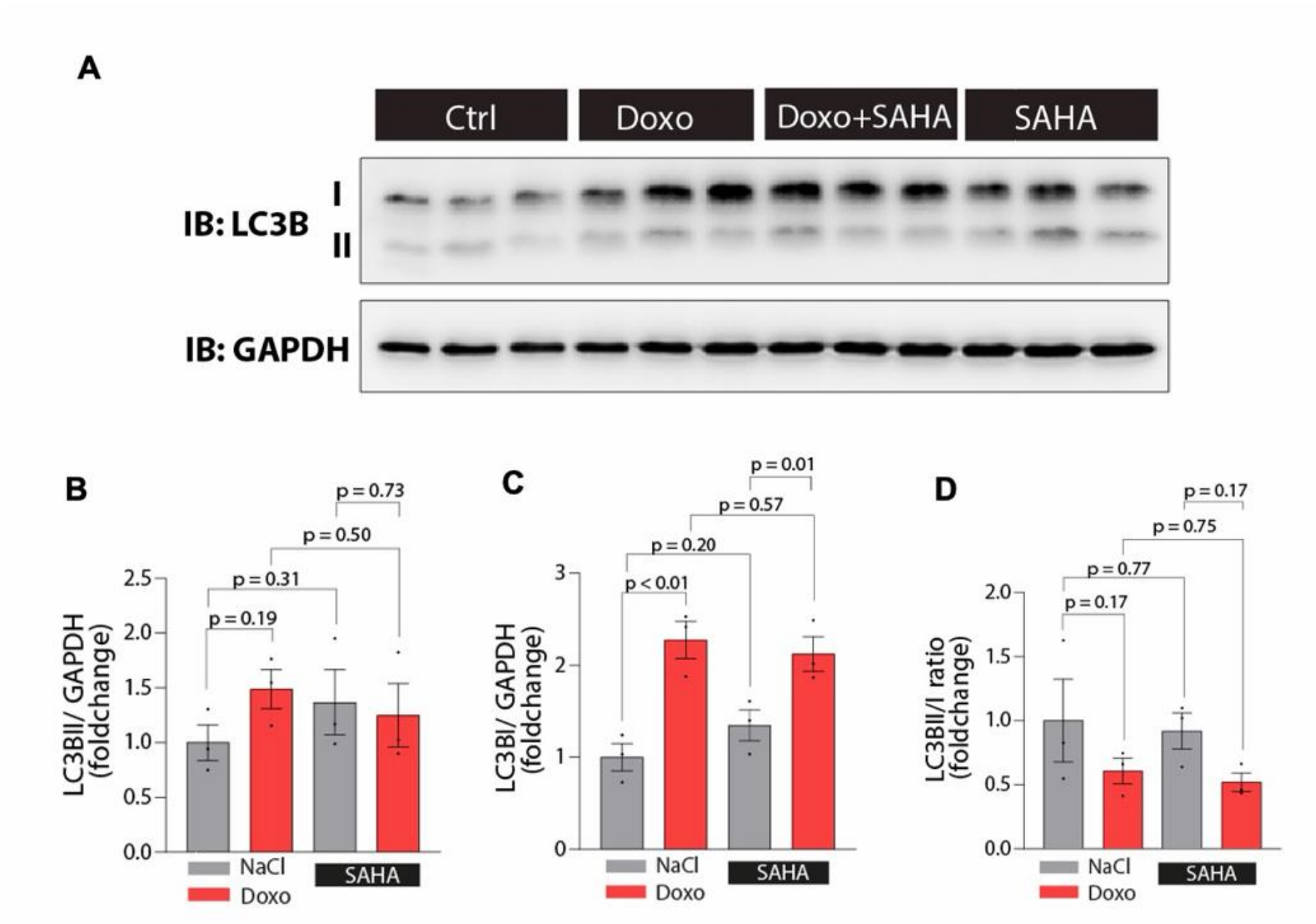

Supplementary Figure 3: (**A**) LC3B I and II levels in human hiPSC-derived CMs treated with Doxo±SAHA. GAPDH was used as a control. (**B**) Quantification of the WB for total LC3B I and II normalized to GAPDH.

To the results paragraph we have added the following: *"[…] In addition, we examined the autophagy marker LC3B by immunoblotting following treatment with doxorubicin and/or SAHA. Doxorubicin treatment significantly increased LC3BI protein levels compared to the control (p < 0.01). Co-treatment with SAHA did not significantly alter the doxorubicin-induced increase in LC3BI expression (p = 0.57) (**Suppl. Figure 2**).[…]"*

And

*"[…] Further, doxorubicin-induced MEF2 activity was attenuated after Topo IIb knockdown in NRVMs with or without SAHA (**Figure 2E**). […] Accordingly, in a short-term in vivo model, in which doxorubicin-induced γH2AX formation was attenuated by co-therapy with SAHA at the protein level. In addition, Caspase 3/7 activity was significantly increased after doxorubicin treatment and reduced to control levels by SAHA co-treatment in vivo. Consistently, TUNEL stainings for the detection of apoptosis in mice treated short-term with doxorubicin and/or SAHA showed increased apoptosis after doxorubicin treatment, which was attenuated by SAHA co-therapy (**Suppl. Figure 7**). […]*

To the discussion paragraph we have added: *"[…] In this model, nuclear retention of HDAC4 and early inhibition of MEF2 activity may blunt doxorubicin-induced DNA*

damage signaling and apoptosis, as observed in our in vivo experiments, potentially by limiting ROS-mediated and/or direct DNA damage responses. However, the specific contributions of HDAC4 and MEF2 to this Topo IIβ-independent protective mechanism require further investigation. […]"

2. Discussion: In the in vivo SAHA and HDAC4-cKO studies, Myh7 is noted to be upregulated by Dox, but it is not shown experimentally that this upregulation results directly from modulation of HDAC/MEF2. Given that there are other transcriptional regulators of Myh7, this should be noted as a limitation in the Discussion.

We completely agree with the reviewer. Previously it was shown that *Myh7* is regulated by MEF2 and HDAC4, as it is a known target.[1] However, it cannot be ruled out that other transcription factors or direct DNA damage response at the promoter may also play a role in its regulation.

We added a new paragraph to address this limitation: *"[…] We detected Topo IIb binding at the Myh7 promoter in vitro, and the transcriptional regulation of Myh7 observed in our in vivo models is consistent with dysregulated MEF2 activity. Nevertheless, additional transcriptional regulation by other doxorubicin-induced events cannot be excluded. […]"*

Minor comments:

1. In addition to listing the citation in the Methods, it would be helpful to include the citation to the publicly available RNAseq dataset used in lines 369-370 within the Results section.

We inserted the citation of the RNA-seq dataset in the results part as well: *"[…] To verify transcriptional dysregulation of the identified genes in human samples, we re-analyzed the publicly available RNA-seq dataset of hiPSC-derived cardiomyocytes exposed for 16 h to vehicle, 1 µM or 2.5 µM doxorubicin (2 sets of duplicate samples per condition, GEO ID GSE106297 [2]) (Figure 1G). […]"*

2. Were doxorubicin and SAHA administered via IP or IV injection?

We apologize for not being clear regarding the administration of the drugs. Both, doxorubicin and SAHA were administered via i.p. injections. We added in the methods part: *"[…]* C57BL/6J mice at the age of 10 weeks were injected 8 times within 2 weeks with doxorubicin in a concentration of 3 mg/kg BW ± 12.5 mg/kg BW SAHA per injection. All drugs were administered via intraperitoneally (i.p.) injections. […]"

Reviewer #2 (Remarks to the Author)

This study proposes a Topoisomerase IIβ–HDAC4–MEF2 axis linking doxorubicin-induced DNA damage to maladaptive transcriptional remodeling in cardiomyocytes, and suggests that the pan-HDAC inhibitor SAHA may offer cardioprotection in this setting. The integration of genomic profiling, molecular

assays, and in vivo mouse models is a strength, and the cardiomyocyte-specific HDAC4 knockout provides supportive genetic evidence for pathway involvement. The identification of 14-3-3 acetylation as a regulator of class II HDAC localization is also intriguing. The idea of repurposing SAHA as a cardioprotective adjunct during anthracycline therapy is appealing; however, all in vivo experiments use SAHA prophylactically, which limits clinical appeal. Demonstrating benefit in a treatment (rather than prevention) paradigm would substantially strengthen translational relevance. Overall, much of the current evidence remains associative, the in vivo effects are modest, and key mechanistic steps are not directly tested. Substantially deeper mechanistic validation and stronger human relevance will be needed to support the proposed pathway and its translational implications.

We thank the reviewer for the feedback. We are happy to revise the manuscript accordingly. Regarding the timing of the SAHA application, we intentionally chose a co-treatment strategy as it would be used in cancer therapy and fits the most to the translational approach. HDAC inhibitors (HDACi) itself are important antiproliferative drugs used in oncology for a wide spectrum of malignancies. Given this potential opportunity, the oncologists would not need to apply HDACi after termination of an anthracycline-based therapy. We propose an anthracycline plus HDACi administration as a co-treatment strategy as it was previously tested in oncological studies [3, 4] to achieve effective antiproliferative therapy with less cardiotoxicity.

Major Comments

1. In Fig. 3F–I, LVEF is the most compelling functional readout, but overall cardioprotective effects of SAHA appear modest. In Fig. 3G–I, interpretation of heart rate, LVESD, and LVEDD is challenging: reduced heart rate does not necessarily indicate cardiotoxicity, and ventricular dilation would typically be expected but is not consistently observed. Consider moving panels 3G–I to the supplement.

We thank the reviewer for this comment. We have now moved the subfigures to the supplementary material (Supplementary figure 11) accordingly. Similarly, Fig. 4G-I moved to Supplementary figure 13.

2. Similarly, antifibrotic effects appear subtle in Fig. 3L, and Fig. 3M shows substantial variability in the control +dox+SAHA group.

We agree with the reviewer that the effects are modest and may have variability. Even though using inbred mouse lines, the cardiotoxicity of doxorubicin may be more or less intense. The variability may be explained by individual differences in pharmacokinetics in the pharmacological model. In response #1 to Reviewer 1 we added further readouts of doxorubicin cardiotoxicity (including measures for autophagy and DNA damage). We apologize for the poor quality of the histologies and have adjusted them (Figure 3H (earlier 3L)):

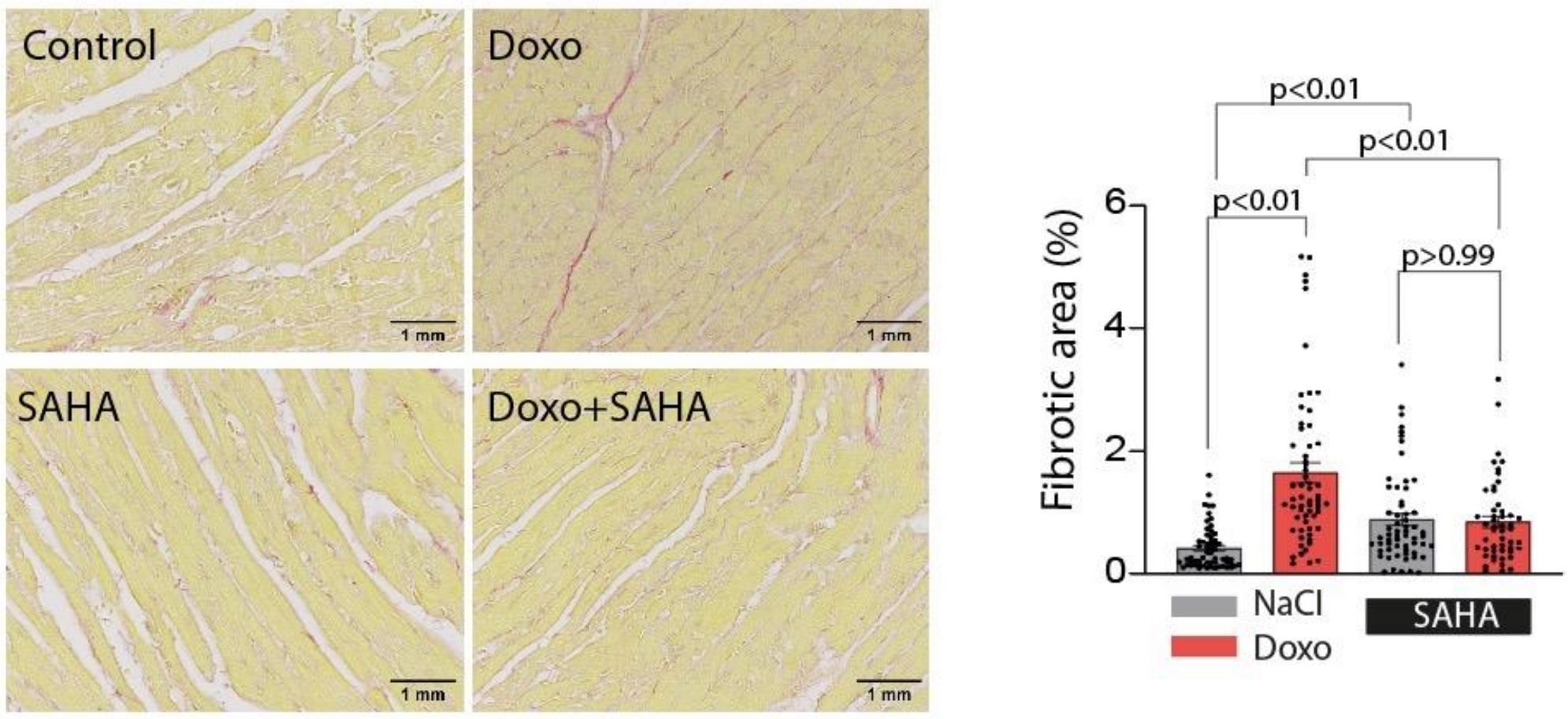

To the discussion paragraph we have added the following: *"[…] cardiac remodeling including fibrosis is only mild in doxorubicin treated animals which speaks for a cell-death independent mechanism of cardiac dysfunction […]"*

3. All in vivo studies administer SAHA concurrently with doxorubicin. It is unclear whether SAHA can reverse established doxorubicin-induced injury. Testing delayed SAHA administration after cardiotoxicity onset would substantially improve translational relevance, as universal prophylactic use may be difficult to implement clinically.

We thank the reviewer for mentioning this issue. We would like to clarify our approach in more detail. Depending on the treated tumor, HDACi therapy may require effective co-treatment therapies [5]. Anthracycline dosing is limited by toxicity, largely cardiac toxicity. In the co-treatment scenario, HDACi and higher anthracycline dosages may be an appealing approach in sarcoma patients. A more aggressive antiproliferative treatment can be tolerable in terms of therapy-limiting toxicity.

We added to the manuscript:

"*[…] The pan-HDACi SAHA is already in use for T-cell lymphoma treatment and has been evaluated in other entities, such as sarcoma. [5] The co-therapy with anthracyclines could allow for improving cancer therapy, [3, 4] and higher dosages of anthracyclines could be tolerated due to potentially less cardiotoxicity. […]*"

4. While TopoIIβ ChIP-seq shows increased enrichment at MEF2-bound promoters following doxorubicin, functional dependence of MEF2 activation on

TopoIIβ is not directly tested. The data currently support association rather than causality. This could be strengthened by TopoIIβ depletion or inhibition followed by assessment of MEF2 activity and endogenous target gene expression, as well as temporal analyses demonstrating that TopoIIβ binding precedes transcriptional activation. In addition, locus-specific validation using CUT&RUN or CUT&Tag (e.g., for TopoIIβ, MEF2, and γH2AX) at representative promoters would help establish whether TopoIIβ recruitment coincides with local DNA damage and MEF2 engagement at the same genomic sites.

We thank the reviewer for this comment. To address the question, we added MEF2 reporter experiments in cardiomyocytes with knockdown of TopoIIb. We observed an attenuated response in MEF2 activity after doxorubicin application when Topo IIb is knocked down. These findings support a role for Topo IIb upstream of doxorubicin-induced MEF2 activation (new Figure 2E). During the project, we have extensively investigated whether doxorubicin-poised Topo IIb is able to cleave the target promoters *ex vivo* (e.g., the *Myh7* promoter), which was described as a possible mode of action in other cells. However, we did not find evidence for such and have not included the data.

To the results paragraph we have added new Figure 2E and suppl. Figure 6:

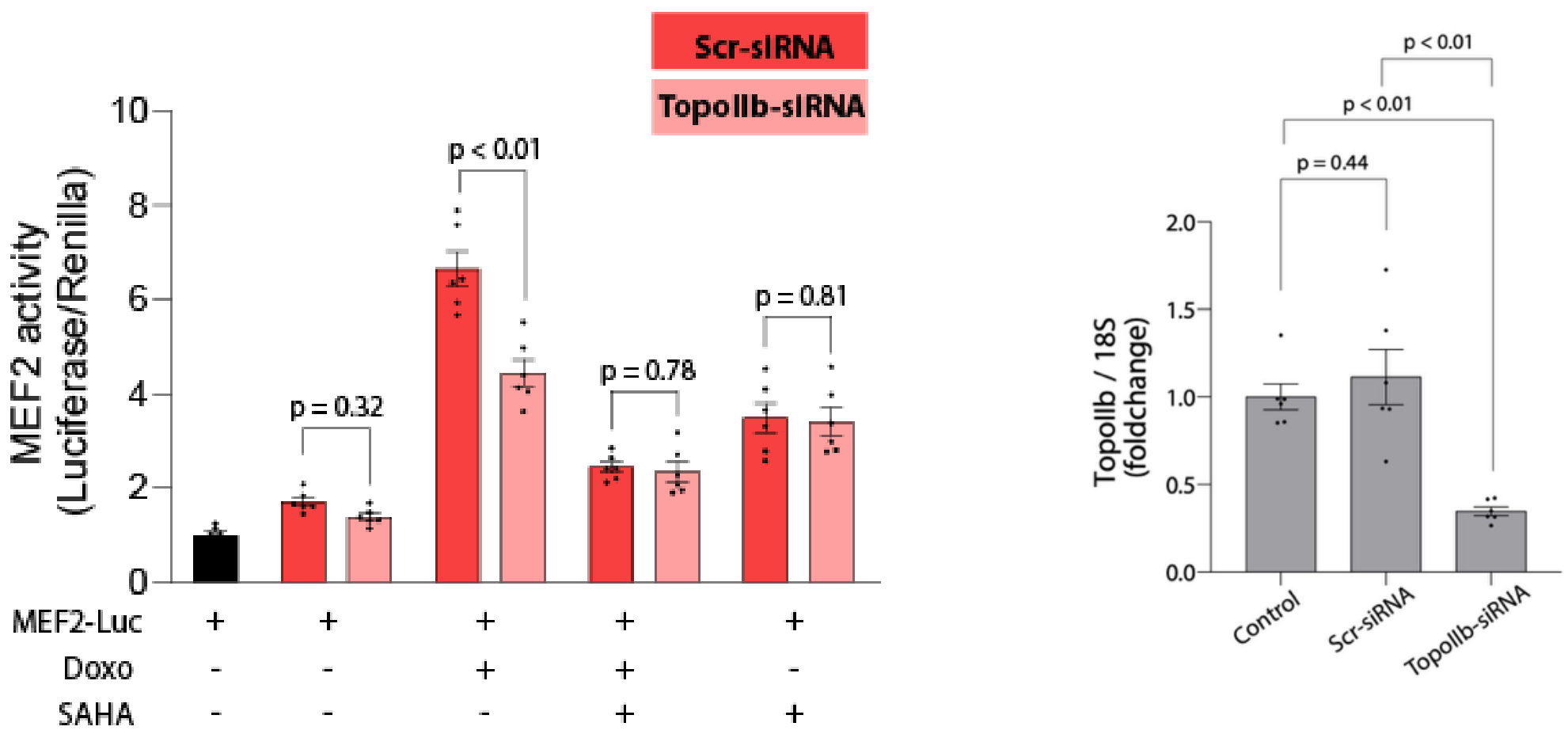

Figure 2E: Topo IIb knockdown attenuates Doxo-induced MEF2 activity in NRVMs. MEF2 activity measured by Luciferase Assay after Doxo ± SAHA treatment (n=6 replicates per treatment group; ANOVA, Bonferroni correction). [knockdown-confirmation on the right side is added as a new suppl. figure 6] **Supplementary Figure 6:** Topoisomerase IIb (Topo IIb) expression levels following siRNA-mediated knockdown with scrambled (Scr-siRNA) or Topoisomerase IIb (Topo IIb)-specific siRNA (TopoIIb-siRNA) in neonatal rat ventricular myocytes (NRVMs) were normalized to 18S ribosomal RNA expression. Data are presented as mean ± SEM from n = 6 biologically independent experiments per treatment group. Statistical analysis was performed using one-way ANOVA followed by Bonferroni's multiple comparisons test; exact p values are indicated above the comparisons.

Regarding locus specificity, we point to Figure 1 where we have already shown that Topo IIb binding sites specifically overlap with MEF2 binding sites at the same genomic loci. In the case of γH2AX, we would not expect a locus-specific enrichment since it is known for γH2AX to spread bidirectionally from DNA double-strand breaks across megabase-scale chromatin domains, rather than being confined locally to the site of damage. [6, 7] However, to investigate whether Doxo-induced γH2AX formation depends on presence of Topo IIb, we performed Topo IIb-knockdown by siRNA in NRVMs. The results show that Doxo-induced γH2AX generation is attenuated after Topo IIb knockdown, supporting a role for Topo IIβ in doxorubicin-induced DNA damage signaling in cardiomyocytes. We have included these data as a new figure in the supplement (Supplementary Figure 5).

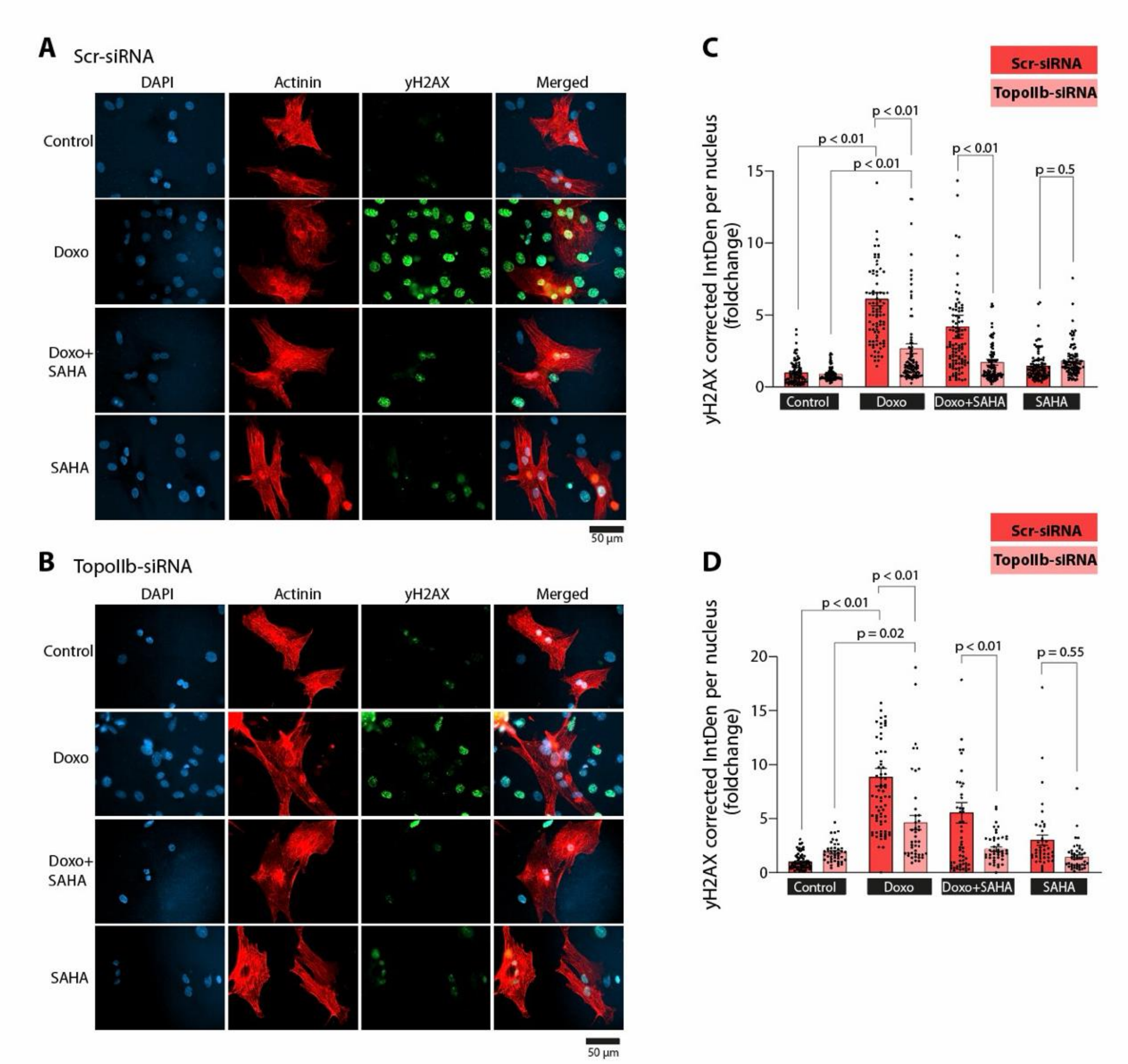

Supplementary Figure 5: Representative immunofluorescence images of phosphorylated histone H2AX (γH2AX) staining in neonatal rat ventricular myocytes (NRVMs) (**A**) transfected with scrambled (Scr-siRNA) or (**B**) Topoisomerase IIb (TopoIIb)-specific siRNA (TopoIIb-siRNA) and treated with doxorubicin (Doxo), suberoylanilide hydroxamic acid (SAHA) or the combination. Cardiomyocytes were identified by α-actinin staining and nuclei were counterstained with DAPI. Scale bar, 50 µm. Quantification of γH2AX fluorescence intensity per nucleus on (**C**) total nuclei and () in cardiomyocytes only. Each data point represents a single nucleus. A total of 10 randomly selected microscopic fields per experimental condition

were analysed. Statistical analysis was performed using one-way ANOVA followed by Bonferroni's multiple comparisons test; exact p values are indicated above the comparisons.

To the results paragraph we have added: *"[…] Additionally, immunostaining in NRVMs revealed that Topo IIb knockdown attenuated doxorubicin-induced formation of the double-strand break marker γH2AX. Co-treatment with SAHA reduced γH2AX formation in scr-siRNA-transfected cells and this effect was significantly enhanced after Topo IIb knockdown (Suppl. Figure 7).[…]"*

To the discussion paragraph we have added: *"[…] Notably, SAHA co-treatment attenuated doxorubicin-induced γH2AX activation even in the absence of Topo IIβ. This suggests that the protective effects of SAHA are not solely mediated by inhibition of Topo IIβ-dependent DNA damage, but may also involve modulation of the HDAC4-MEF2 axis. In this model, nuclear retention of HDAC4 and early inhibition of MEF2 activity may blunt doxorubicin-induced DNA damage signaling and apoptosis, as observed in our in vivo experiments, potentially by limiting ROS-mediated and/or direct DNA damage responses. However, the specific contributions of HDAC4 and MEF2 to this Topo IIβ-independent protective mechanism require further investigation. […]"*

5. SAHA is a broad-spectrum HDAC inhibitor with multiple histone and non-histone targets. Although HDAC4 dependence is demonstrated genetically in vivo, parallel or off-target mechanisms remain possible. This concern is underscored by HDAC6 knockdown also suppressing MEF2 activity and by the broad effects of SAHA on 14-3-3 acetylation. Additional specificity controls would help, such as comparison with another pan-HDAC inhibitor (e.g., trichostatin A), inclusion of HDAC6-selective inhibition (e.g., tubastatin A), or testing whether SAHA directly alters TopoIIβ chromatin binding.

We thank the reviewer for raising this important point. To address whether the observed effect is specific to the proposed mechanism or reflects a broader consequence of HDAC inhibition, we repeated MEF2 reporter assays using trichostatin A (TSA) in parallel with SAHA. TSA phenocopied the inhibitory effect of SAHA on MEF2 activity, supporting the interpretation that this response reflects a class effect of pan-HDAC inhibition rather than a SAHA-specific off-target effect. These data have now been included as new Supplementary Figure 3.

In addition, we tested pharmacological HDAC6 inhibition using tubastatin A. Consistent with our siRNA-mediated HDAC6 knockdown experiments in NRVMs, it also reduced MEF2 activity. Together, these pharmacological and genetic data support a role for HDAC6 in regulating the 14-3-3-HDAC4-MEF2 axis. However, we focused our mechanistic and translational analyses primarily on SAHA because it is clinically approved and therefore more directly relevant for potential therapeutic translation.

Finally, we agree that SAHA may affect multiple histone and non-histone targets. We have therefore revised the Discussion to explicitly state that parallel or additional HDAC-independent mechanisms cannot be excluded, despite the genetic evidence supporting HDAC4 dependence *in vivo*. We added a new Supplementary Figure 5:

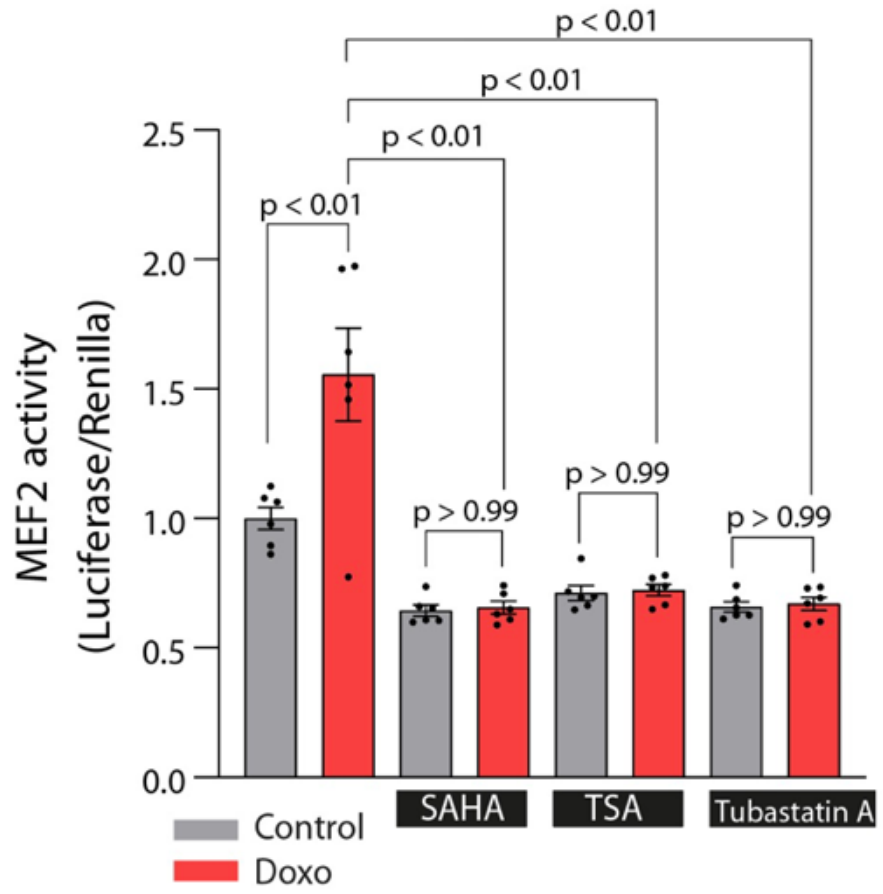

Supplementary Figure 3: Doxo-induced MEF2 activity is inhibited by pan HDACi SAHA, TSA or Tubastatin A in NRVMs (n=6 per treatment group; ANOVA, Bonferroni correction.)

To the results paragraph we added: *"[…] Furthermore, we confirmed this effect using the pan-HDAC inhibitor trichostatin A (TSA). Similar to SAHA, TSA and the HDAC6-specific inhibitor effectively inhibited the doxorubicin-induced increase in MEF2-dependent transcriptional activity and restored MEF2 activity to levels comparable to those of untreated controls (Suppl. Figure 3). […]"*

To the discussion paragraph we added: *"[…] The overlapping effects of SAHA and TSA may be explained by their shared inhibition of class I and II HDACs. Notably, the HDAC6-selective inhibitor tubastatin A also suppressed MEF2 activity, suggesting that HDAC6 may contribute to 14-3-3 deacetylation in this setting. This interpretation is further supported by siRNA-mediated HDAC6 knockdown in NRVMs, which similarly reduced MEF2 activity. […]"*

6. The mechanistic framework around 14-3-3 acetylation remains incomplete. While lysine 50 acetylation is implicated in regulating class II HDAC localization, upstream acetyltransferase(s) are not identified. Partial pathway resolution—for example, testing p300/CBP involvement pharmacologically or genetically, or assessing whether doxorubicin alone induces partial 14-3-3 acetylation—would strengthen this section. Rescue experiments using acetylation-deficient versus acetyl-mimetic 14-3-3 mutants would also help establish whether this modification is necessary and sufficient for HDAC4 nuclear retention and MEF2 repression.

We appreciate this helpful comment. Based on our data, we interpret 14-3-3 hyperacetylation primarily as a consequence of impaired HDAC-dependent deacetylation upon SAHA treatment rather than as a doxorubicin-induced activation of upstream acetyltransferases. To address this point experimentally, we assessed whether doxorubicin alone alters the acetylation status of 14-3-3. We did not detect

increased 14-3-3 acetylation after doxorubicin treatment alone, supporting the interpretation that 14-3-3 hyperacetylation is specifically linked to HDAC inhibitor treatment in our experimental setting. We have added these data to the revised manuscript.

We agree that identifying the upstream acetyltransferase(s) responsible for 14-3-3 lysine acetylation would further refine the pathway. However, this question is beyond the scope of the current revision, which focuses on the functional consequences of HDAC inhibition and HDAC4-MEF2 regulation in doxorubicin-induced cardiotoxicity. We have revised the discussion to acknowledge that the acetyltransferase(s) involved in this modification remain to be identified.

Regarding acetylation-deficient or acetyl-mimetic 14-3-3 mutants, we agree that such experiments could provide additional mechanistic insight. However, we do not consider them a straightforward approach in this context, because the subcellular localization of class IIa HDACs is strongly governed by phosphorylation-dependent 14-3-3 binding and nuclear export. Thus, mutation of individual acetylation sites may be difficult to interpret, as changes in phosphorylation-dependent binding, kinase activity, or nuclear export could override or mask acetylation-dependent effects. We have therefore refrained from adding these experiments to the current study, while acknowledging this as an important area for future work. In the results paragraph, we have included a new Figure 2K.

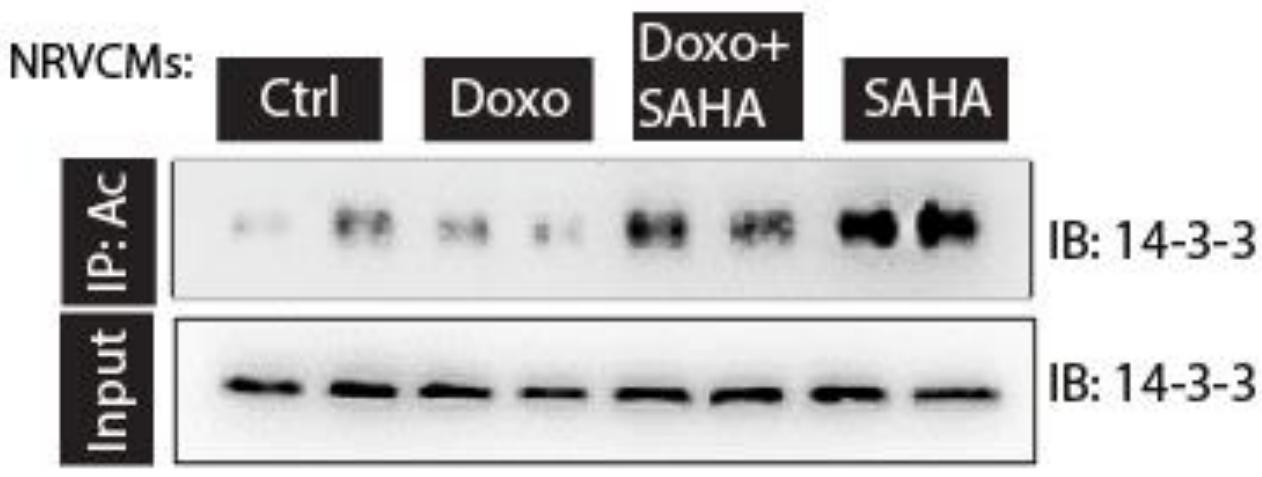

Figure 2K: Immunoprecipitation of endogenous pan-acetylation sites ± Doxo (300 nM) and/or SAHA (3 µM) pretreatment (3 µM) in NRVMs as indicated. Immunoblotting in pulldown and input for 14-3-3 is shown. IP: Immunoprecipitated antibody; IB: Immunoblotted antibody.

To the results paragraph we added: *"[…] In contrast, doxorubicin treatment alone did not induce detectable acetylation of endogenous 14-3-3. Co-treatment with doxorubicin and SAHA resulted in levels of 14-3-3 acetylation comparable to those observed with SAHA alone (**Figure 2K**).[…]"*

To the discussion paragraph we added: *"[…] The lack of 14-3-3 hyper-acetylation after doxorubicin treatment alone argues against doxorubicin-induced activation of upstream acetyltransferases as the primary driver of this modification. […]"*

7. Human relevance remains limited. The re-analysis of published hiPSC-derived cardiomyocyte RNA-seq lacks sufficient contextual detail, and key mechanistic features (MEF2 activation, TopoIIβ promoter binding, HDAC4 localization, 14-3-3 acetylation, SAHA rescue) are not directly examined in human cardiomyocytes.

We thank the reviewer for this important comment. We agree that validation in hiPSC-derived cardiomyocytes strengthens the translational relevance of our findings. In the revised manuscript, we therefore added new experiments in hiPSC-derived cardiomyocytes showing that doxorubicin increases MEF2 reporter activity and that this response is attenuated by SAHA co-treatment. These data have been included as new Figure 1K and Supplementary Figure 3.

We would also like to emphasize that the central mechanistic conclusions of our study are supported by multiple complementary model systems. NRVMs served as a well-established primary cardiomyocyte model for mechanistic perturbation experiments, while key findings were extensively validated *in vivo* using genetic mouse models. In addition, with the newly added hiPSC-derived cardiomyocytes data, these experiments support the relevance of the HDAC4-MEF2 axis and its modulation by SAHA across primary, *in vivo*, and human cardiomyocyte models.

Into the results paragraph, we added a new Figure 1K and Supplementary Figure 3:

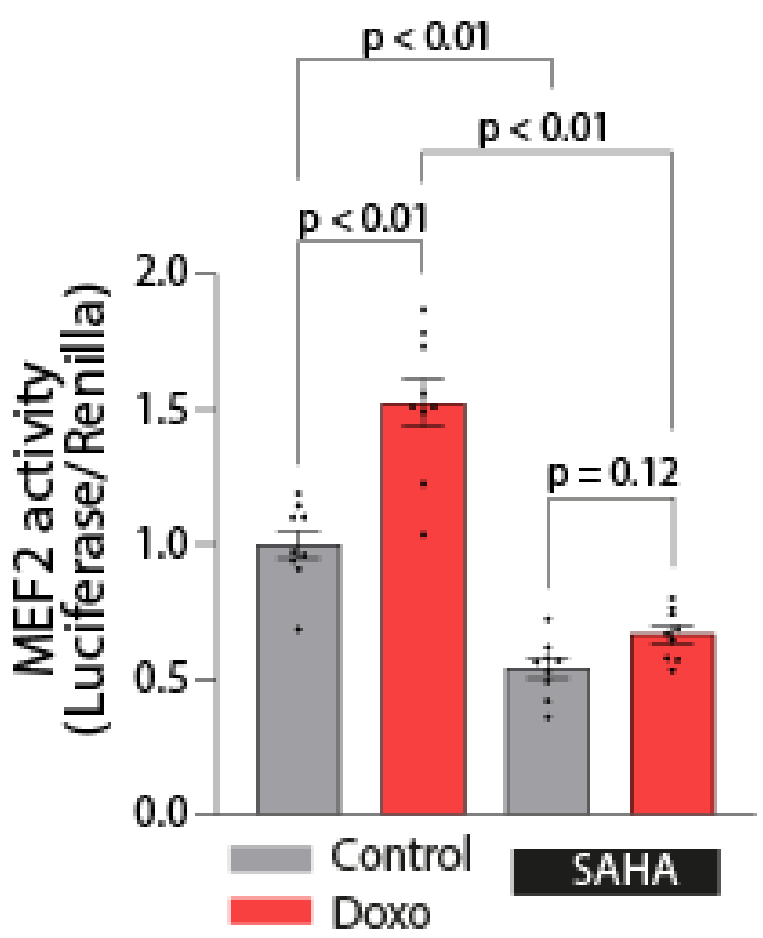

Figure 1 K: Doxo-induced MEF2 activity is reversed by co-treatment with SAHA in human IPSC-derived CMs (n= 9 replicates per treatment; ANOVA with Bonferroni correction).

To the results paragraph we added: *"[…] To determine whether these transcriptional changes are associated with enhanced MEF2 activity, we performed a MEF2 luciferase reporter assay in hiPSCs treated with doxorubicin in the presence or absence of the HDAC inhibitor SAHA (Figure 1K).[…]"*

8. Translational confidence would be improved by targeted validation in human cardiomyocytes or integration of existing human datasets (e.g., bulk or single-cell cardiac RNA-seq from doxorubicin-exposed samples) to assess whether the transcriptional programs identified in mice are similarly engaged in human disease.

We thank the reviewer for raising this point. We extensively looked for such datasets and cross-species validation. Unfortunately, there are no available datasets of good quality published to compare with, apart from the dataset GEO ID GSE106297 we already used. Overall, there are no datasets of sn-RNA/sc-RNA-seqs which could compare the cardiomyocyte-specific response to Doxo.

Minor Comments

Provide brief contextual information on hiPSC-derived cardiomyocytes (e.g., healthy background, general maturation state).

We thank the reviewer for this comment. In the renalyzed datasets, hiPSCs were differentiated into cardiomyocytes using the protocol from Lian et al. [8]: Wnt activation (CHIR99021) followed by Wnt inhibition (IWP2) in serum-free RPMI/B27 medium, yielding beating clusters after 7–14 days. This corresponds to a fetal-like cardiomyocyte state, as is typical for hiPSC-derived cardiomyocytes generated by monolayer-based Wnt modulation protocols.

We added to the results part:

"[…] hiPSCs were differentiated into cardiomyocytes using the protocol from Lian et al. [8]: Wnt activation (CHIR99021) followed by Wnt inhibition (IWP2) in serum-free RPMI/B27 medium, yielding beating clusters after 7–14 days, which corresponds to a fetal-like cardiomyocyte state. […]"

New experiments in the revised version of the manuscript which include hiPCS-derived cardiomyocytes are now fully described in the methods section and results are added into the results paragraph.

Methods line 131: TMP195 is introduced without description; please clarify its target and purpose.

We thank the reviewer for this comment. We added to the Methods:

"[…] The selective class IIa histone deacetylase inhibitor TMP195 was administered in 10 weeks-old WT animals (C57BL/6J) in a dose of 15mg/kg BW per injection (i.p.) with total injections of 7 (Doxo, 3 mg/kg BW/per injection) within 2 weeks. […]"

Clarify rationale for use of rat cardiomyocytes in some experiments and mouse models in others.

We appreciate this comment. NRVMs are an established and reproducible model for *in vitro* cardiomyocyte experiments and very much like neonatal mouse

cardiomyocytes according to molecular signaling but higher in preparation efficiency and lower in costs. For *in vivo* studies, we used mouse models to allow the use of genetically modified animals, which are based on mouse strains in most cases. Moreover, results from mice are more comparable to those from published studies.

Figure 1C: improve color contrast.

We adjusted the color contrast accordingly.

Line 372: define FPKM at first use.

We added to the methods part:

"[…] Data from hiPSC-cells were obtained from Gene Expression Omnibus (GEO ID GSE106297)[2] and re-analyzed with normalized count matrices (Fragments Per Kilobase of transcript per Million mapped reads, FPKM). […]"

Line 380: clarify whether experiments were performed in mouse or rat systems.

We adjusted the following paragraph: *"[…] Interestingly, in luciferase reporter assays in NRVMs, we found a concentration-dependent inhibition of MEF2 by SAHA treatment (**Figure 2A**). […]"*

Indicate whether sex-dependent differences were assessed.

We performed the animal experiments in mixed cohorts of female and male animals. Numbers of male and female animals were equal in all groups and experiments. Due to lower animal numbers per sex, we did not explicitly perform sex-specific analyses. We added the number of females und males used in each experiment in the figure legends.

We added to the manuscript:

"[…] All experiments were done in mixed cohorts of female and male animals. […]"

Clarify whether SAHA-only treatment groups were consistently included across transcriptional analyses.

The SAHA only group was included in RNA-seq of WT animals. Due to limited number of genetically modified animals in the HDAC4-KO study, we did not perform these transcriptional analyses with the SAHA-only group.

Reviewer #4 (Remarks to the Author):

The manuscript by Eksi and colleagues reveals that anthracycline-induced cardiotoxicity is driven in part by Topoisomerase IIβ (Topo IIβ) accumulation at cardiomyocyte gene promoters, where doxorubicin enhances MEF2-dependent transcription. This pathological response is attenuated by the pan-HDAC inhibitor SAHA. Mechanistically, SAHA promotes acetylation of 14-3-3, disrupts its interaction with HDAC4/5, facilitates their nuclear accumulation, represses MEF2-driven transcription, and ultimately mitigates doxorubicin-induced cardiotoxicity in vivo. Overall, the study highlights an epigenetic mechanism underlying anthracycline cardiotoxicity and suggests that HDAC inhibition has promising translational value as a cardioprotective strategy for patients receiving anthracycline therapy.

Major

1. Introduction section: the third paragraph of the Introduction is hard to follow for readers that are not experts in the field of HDAC signaling, consider rephrasing.

We appreciate the reviewer's comment and have now revised the paragraph accordingly: *"[…] HDACs are divided into several subclasses with distinct enzymatic properties and functions in the heart. Class I and II HDACs, have both been implicated in the regulation of cardiac hypertrophy and pathological cardiac remodeling. Class I HDACs, including HDAC1, HDAC2, HDAC3, and HDAC8, have a high deacetylase activity and therefore considered direct enzymatic targets of HDACi. Inhibition of class I HDACs has been linked to altered expression of antihypertrophic genes. In contrast, class II HDACs have more diverse regulatory function. Class IIa HDACs, including HDAC4, HDAC5, HDAC7, and HDAC9) have limited deacetylase activity and act primarily as signaling-responsive regulators by shuttling from the nucleus into the cytosol. They counteract pathological cardiac remodeling by direct inhibition of transcription factors via complexes with other chromatin-modifying proteins. For instance, genetic deletion of the class IIa HDAC 'HDAC5' leads to a higher sensitivity to prohypertrophic stimuli in a genetic model of cardiac hypertrophy. The class IIb HDAC 'HDAC6' has been implicated in mediating cardiac stress response upon Angiotensin II administration. Because of their reduced deacetylase activity, class II HDACs are unlikely to represent direct enzymatic targets of HDACis. They are regulated primarily through non-enzymatic mechanisms. Stimulus-dependent nucleocytosolic shuttling depends on their binding to the chaperone protein 14-3-3. When retained in the nucleus, HDAC4 and 5 inhibit the transcription factor Myocyte Enhancer Factor-2 (MEF2), essential for cardiomyocyte hypertrophy and pathological cardiac remodeling. The potential role of class II HDACs in mediating the antihypertrophic effects of HDACis remains incompletely understood. […]"*

2. Material and Methods section: be more precise on describing the sex of mouse models used. For obvious reasons, anthracycline use for breast cancer is more prevalent in female patients so that a consideration of gender is important in this setting. Further, it was noticed that wild-type animals used were c57BL6J, while the HDAC4 KO mice are on a c57BL6N background. Did the authors notice differences in cardiac function between the genetic strains?

We thank the reviewer for this comment. For the *in vivo* experiments, we used equal numbers of male and female animals in all groups. We did not perform sex-specific subgroup analyses due to reduced power. The reviewer correctly points on the fact that most frequently breast cancer patients receive doxorubicin-based therapies in clinical practice. However, most breast-cancer protocols do not reach a critical dosage of >250mg/m2. Specifically, cancer patients diagnosed with lymphoma, or sarcoma receive toxic dosages in a relevant number and sex distribution in these entities is balanced between male and female patients. Thus, we believe that a study design of the mixed cohorts may better reflect a clinical setting. To be clearer on that, we have now added the numbers of female and male animals used in each experiment to the figure legends.

Regarding different strains, we did see a slightly more pronounced phenotype in the C57BL/6N animals as compared to C57BL/6J. The higher sensitivity is known for C57BL/6N animals but seems not to be detrimental in our ‘low-dose long-term’ doxorubicin model. Importantly, genotype-dependent conclusions were drawn from comparisons within the same strain background and with the appropriate controls. Thus, potential strain-related differences do not confound the interpretation of the HDAC4 genetic experiments. At baseline, we did not observe relevant differences regarding cardiac function. Below, we plotted the baseline LVEF of WT animals comparing both strains.

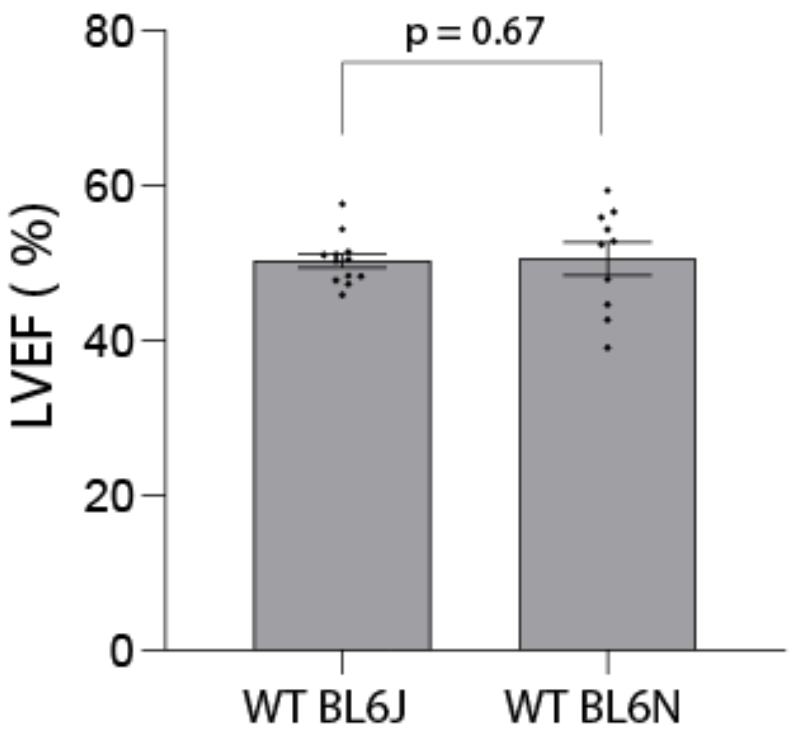

Figure: Cardiac function at baseline does not differ between strain C57BL6J (6 female/ 6 male) and C57BL6N. WT C57BL6J n=12; 6 female/ 6 male and C57BL6N n=10; 3 female/ 7 male; Mann–Whitney U test was performed for statistical analysis.

3. Results: how does a concentration of 1 µM - 2.5 µM doxorubicin correlate with dosages used in patients? In Fig1H-J there seems no evidence of a dose dependent increase, was this expected?

We thank the reviewer for bringing up this point. In patients, treated with 75mg/m2, peak plasma concentrations reach around 4.7µM (Cmax of 2570ng/ml) [9],

therefore, the concentrations used in vitro, 1-2.5µM, are within a clinically relevant peak plasma range. While *Myh7* did not show a strictly concentration-dependent regulation, *Myl2* appeared to respond in a concentration dependent manner. This may reflect a non-linear transcriptional regulation and/or timing of sample collection. To the discussion paragraph we have added the following:

"[…] Although the in vitro concentrations of 1–2.5 µM doxorubicin are within the range of clinically observed peak plasma concentrations, the lack of a strictly dose-dependent increase in Myh7 expression may reflect non-linear transcriptional regulation and/or the timing of sample collection. […]"

To address this point *in vivo*, we measured doxorubicin concentrations in the experiment that additionally used the pharmacological HDAC4 inhibitor, where we observed an increased mortality. During the first 1-3 hours after administration, doxorubicin concentrations were lower than those reported in patients treated with a 75 mg/m² protocol. However, after 6h, doxorubicin concentrations in the plasma were comparable between human and mice. [9] Co-treatment with TMP, which was associated with increased mortality, resulted in markedly higher concentrations of both, doxorubicin and its toxic downstream metabolite doxorubicinol, providing a plausible explanation for the increased toxicity observed. We now provide these data in a supplemental figure 5.

The protocol used in our manuscript was previously adjusted and extensively used in the lab of Rick Kitsis [10] and is accepted as a standard protocol, reflecting mild but consistent cardiotoxic effects while keeping antiproliferative effects.

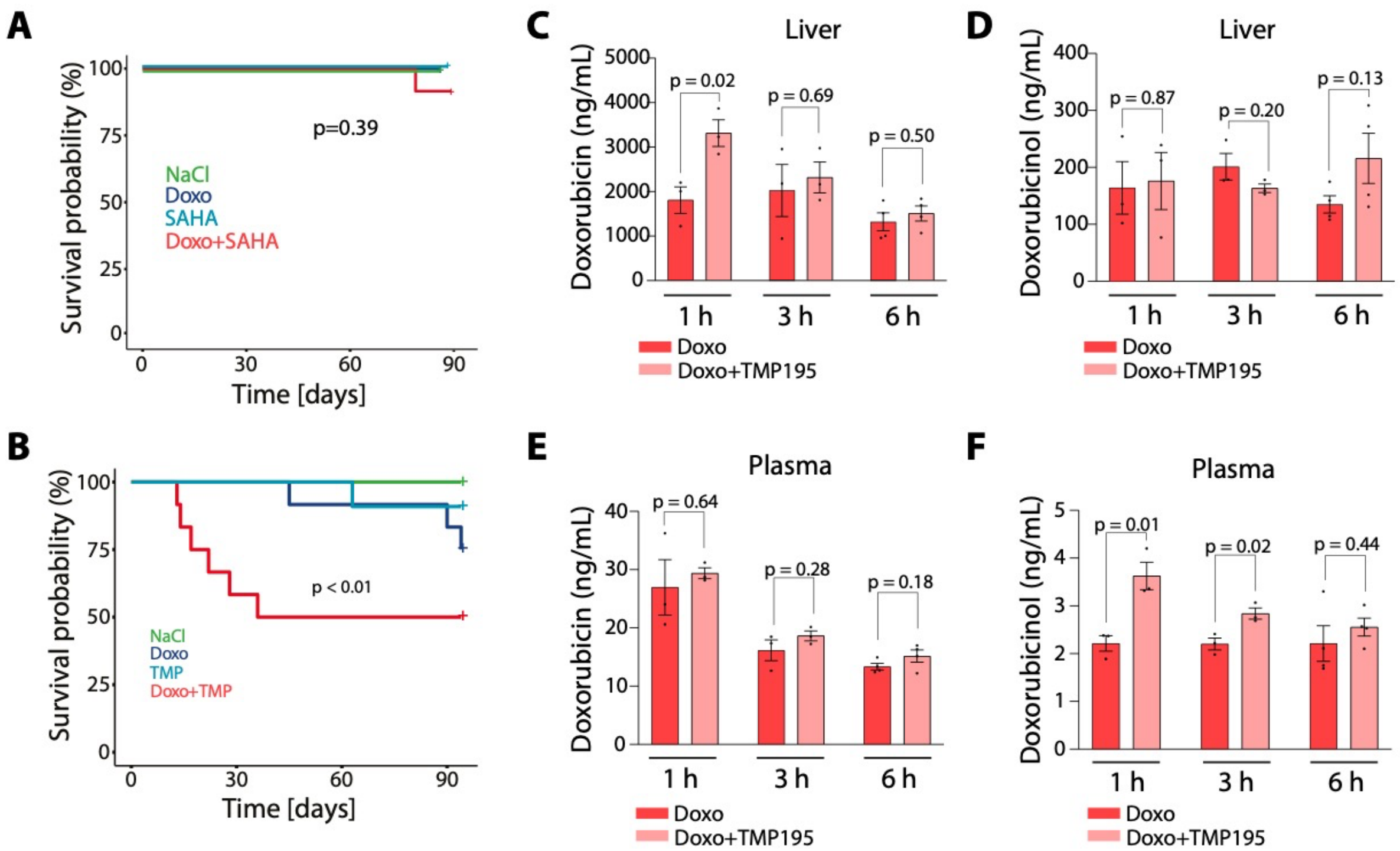

Supplementary Figure 9: Mortality analysis and effect of TMP195 on doxorubicin concentrations in liver and plasma. (A) Kaplan Meier curve to show survival probability (%) over time of indicated groups. P-value calculated via log-rank test. (B) Kaplan Meier survival

analysis shows a significant decrease in survival probability in Doxo/TMP195 co-treatment (log-rank test). Doxorubicin and doxorubicinol concentrations were measured in liver tissue (C, D) and plasma (E, F) at 1, 3, and 6 h following treatment with doxorubicin (Doxo) alone or in combination with TMP195 (Doxo+TMP195). Data are presented as mean ± SEM, with individual biological replicates shown as dots. Statistical significance between groups at each time point was determined using an unpaired two-tailed Student's t-test; exact p-values are indicated above the comparisons.

To the results paragraph we added the following: *"[…] Data from the short-term model indicate that TMP195 interferes with the hepatic handling of doxorubicin, leading to increased exposure to doxorubicin and its toxic metabolite doxorubicinol. This pharmacokinetic interaction most likely explains the increased toxicity and mortality observed in Doxo+TMP195 treated animals (Suppl. Figure 9). […]"*

4. The selective knockdown of HDAC1-11 in Figure 2 was an elegant approach to decipher which HDAC isoform is involved in doxorubicin/SAHA responsiveness. Panel E misses size bars and the immunofuorence images seem out of focus, however, while the blots in panels K and L could use a bit more background (less contrast).

We thank the reviewer for this comment and adjusted the images accordingly.

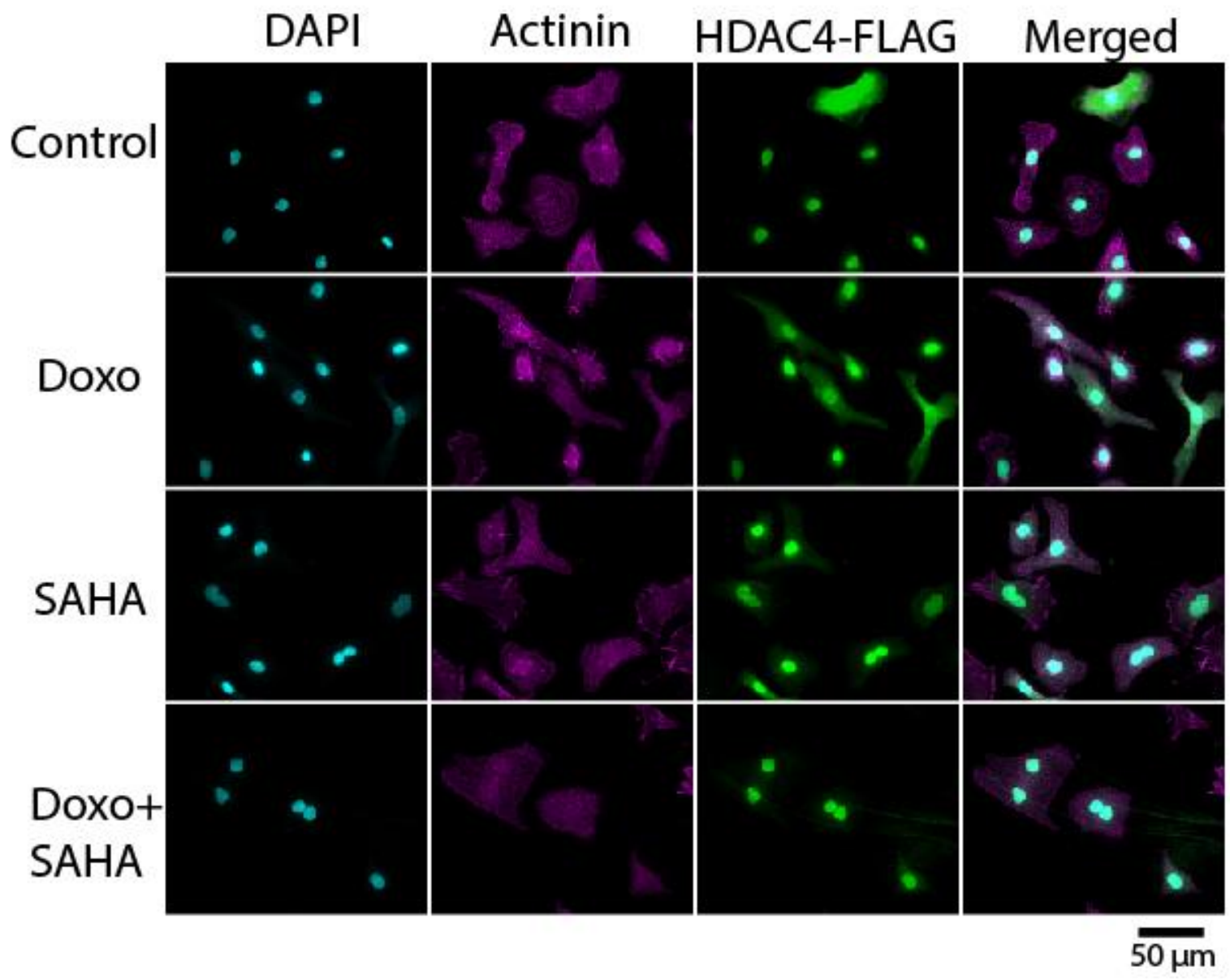

5. The differences in panels A and B (Figure 3) in cardiac hypertrophy response are confusing. Is this accounted for by a loss of body weight in response to doxorubicin? Regarding the changes in the rest of the parameters in the study (<LVEF, < LVEDD, < LVPW), what type of remodeling are we observing? Is this a classical HFrEF response? Did the authors record indices of diastolic function? Panel L and M in Figure 3 and 4, respectively, seem to be out of focus.

We thank the reviewer for this comment. Indeed, loss in body weight accounts for the difference in the ratios. This change during doxorubicin treatment is a typical

consequence from anthracycline therapy. To avoid this confusion, we have now taken out the HW/BW ratio and show BW alone. The observed cardiac dysfunction which is defined as a decreased systolic function is associated with increased markers of cardiac damage, such as double strand breaks and apoptosis (see also answer to the next question). Unfortunately, we have not collected data on diastolic function and cannot comment on that. The doxorubicin-associated cardiomyopathy is frequently associated with a mild cardiac atrophy and possibly explains the <LVEF, < LVEDD, < LVPW - phenotype. We adjusted the image settings of Figure 3 and 4.

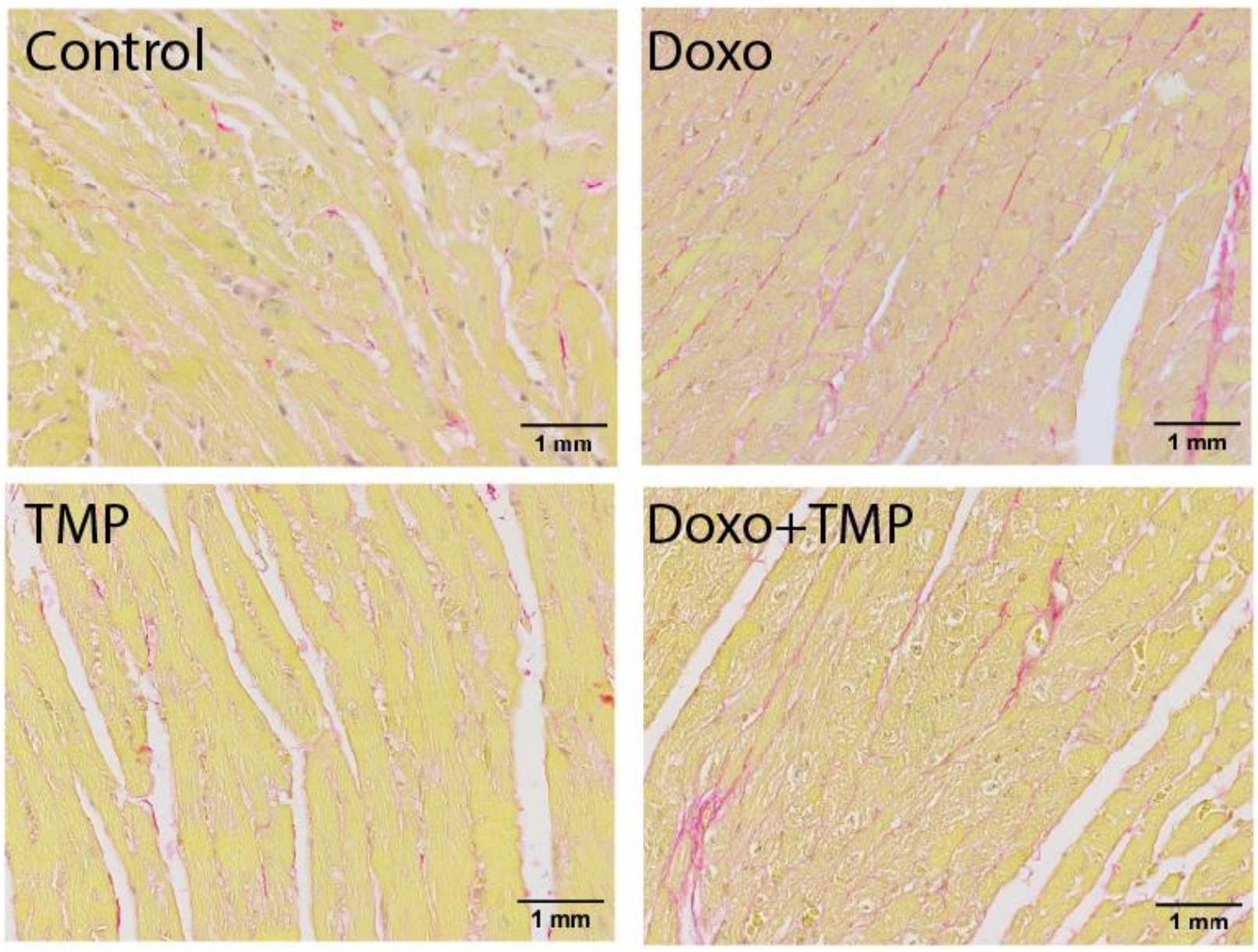

New Figure 3 H (previously 3M)

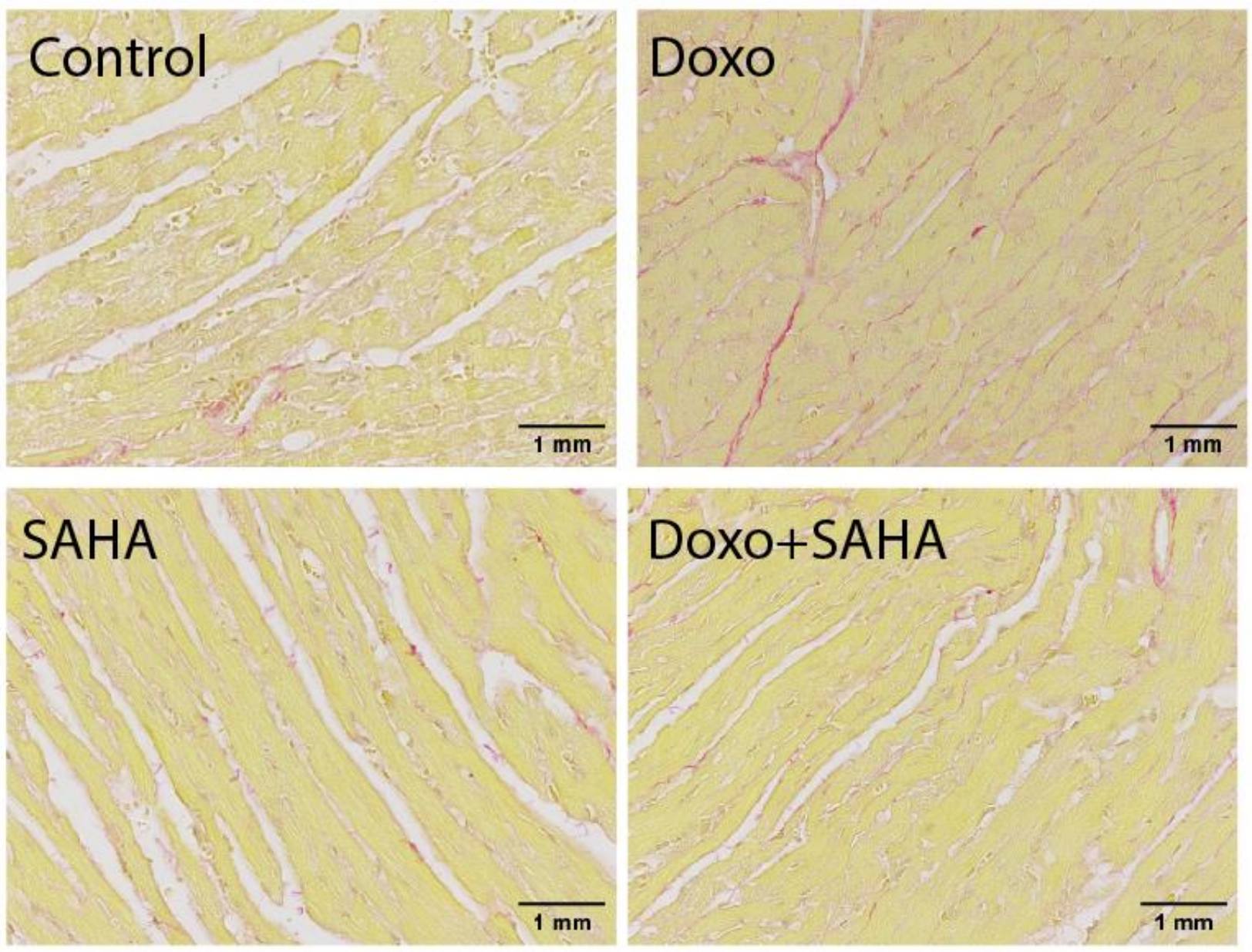

New Figure 4 H (previously 4M)

6. Anthracyclin-induced “cardiomyopathy” is classically accompanied by high levels of apoptosis, possibly this could be an additional readout parameter for the studies.

We thank the reviewer for the comment. In our initial manuscript we were focusing on long-term effects of doxorubicin therapy. Therefore, we would not expect increased apoptosis, weeks after initial stress. To address this point and to investigate a potential effect on apoptosis by SAHA, we have included additional data from a short-term experiment with only two injections of doxorubicin. As readouts, we investigated TUNEL positive cells and Caspase 3/7 activity readouts for apoptotic cell death as well as yH2AX as a marker of DNA damage. In all three readouts, we see an increase of the pathological marker 24h after the last doxorubicin treatment and a marked reduction in animals co-treated with SAHA. We have included these data as a new supplemental figure 7.

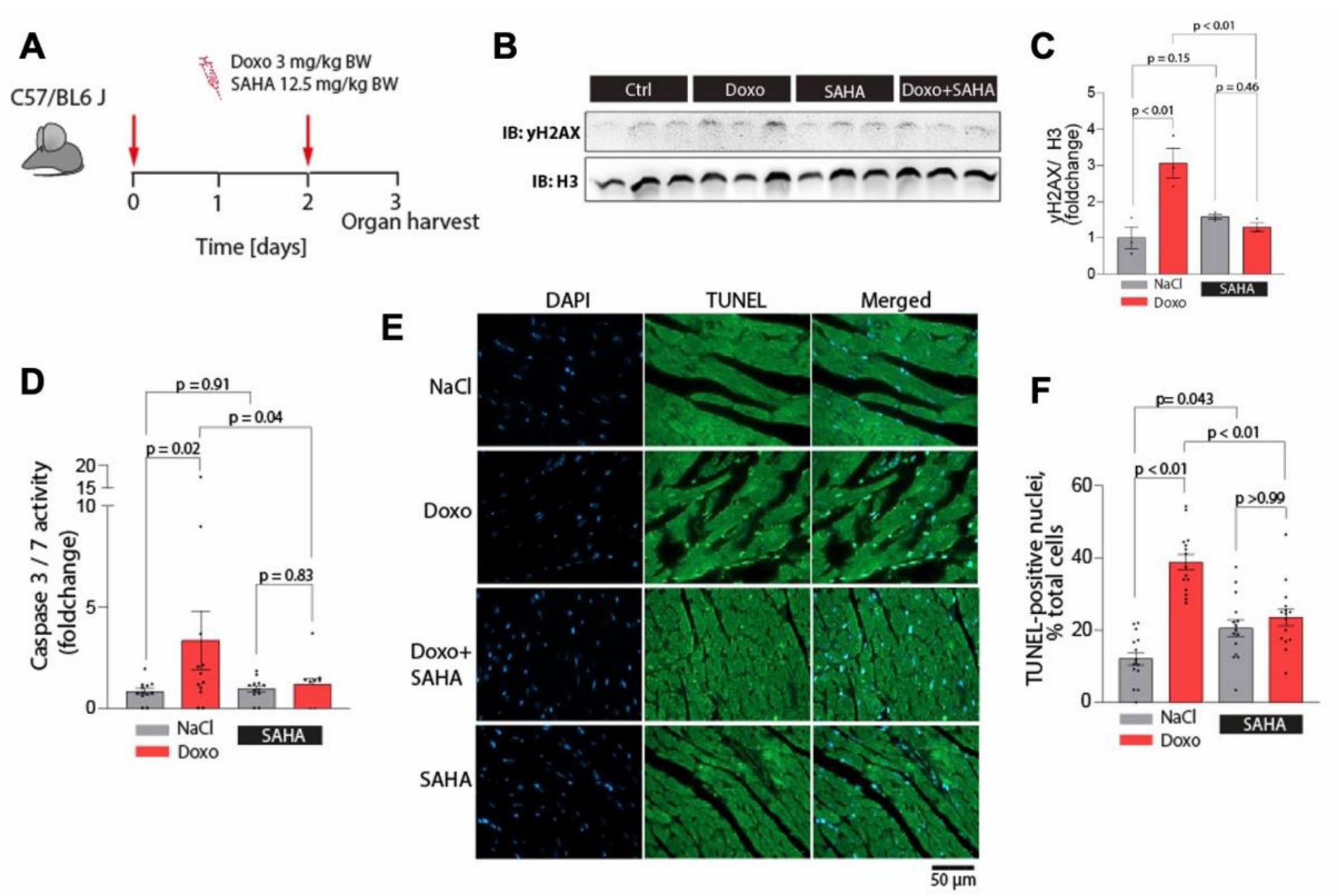

Supplementary Figure 7: SAHA attenuates doxorubicin-induced DNA damage and apoptosis *in vivo*. (**A**) Experimental design of the *in vivo* short-term treatment protocol in C57BL/6J mice receiving doxorubicin (3 mg/kg BW) SAHA (12.5 mg/kg BW). (**B**) Representative immunoblot analysis of γH2AX expression in cardiac tissue from NaCl, Doxo ±SAHA-treated mice. Histone H3 served as loading control. (**C**) Quantification of γH2AX protein levels normalized to H3. (**D**) Caspase-3/7 activity in cardiac tissue lysates from NaCl, Doxo ±SAHA-treated mice (n= 10 mice per treatment group). (**E**) Representative images of TUNEL staining in cardiac sections obtained from NaCl, Doxo ±SAHA-treated mice. Nuclei were counterstained with DAPI. Scale bar: 50 μm. (**F**) Quantification of TUNEL staining represented as the percentage (%) of TUNEL positive nuclei of counted total nuclei in n= 3 mice per group. For each mouse 10 images were taken. For statistical test ANOVA with Bonferroni correction was used.

To the results paragraph we added the following: *“[…] Co-treatment with SAHA reduced γH2AX formation […] Similar effects were confirmed in a short-term in vivo model, in which doxorubicin-induced γH2AX formation was attenuated by co-treatment with SAHA at the protein level. In addition, Caspase 3/7 activity was significantly increased after doxorubicin treatment and reduced to control levels by SAHA co-therapy in vivo. Consistently, TUNEL stainings for the detection of apoptosis in mice treated short-term with doxorubicin and/or SAHA showed increased apoptosis after doxorubicin treatment, which was attenuated by SAHA co-therapy (**Suppl. Figure 7**)[…]”*

To the discussion paragraph we added the following: *“[…] Notably, doxorubicin-induced apoptosis was also blunted by SAHA co-therapy, which speaks for a protective effect against ROS and/or direct DNA damage response when HDAC4 is localized in the nucleus. […]”*

7. The failure in HDAC4 KO to respond to SAHA is very strong evidence for the involvement of this isoform in the signaling underlying anthracyclin-induced cardiac toxicity, making Figure 5 an essential part of the strength of this study. One criticism pertains to the resolution of panel F.

We thank the reviewer for this comment and apologize for the bad quality of the images. We have now included improved resolution of the images in Figure 5 F.

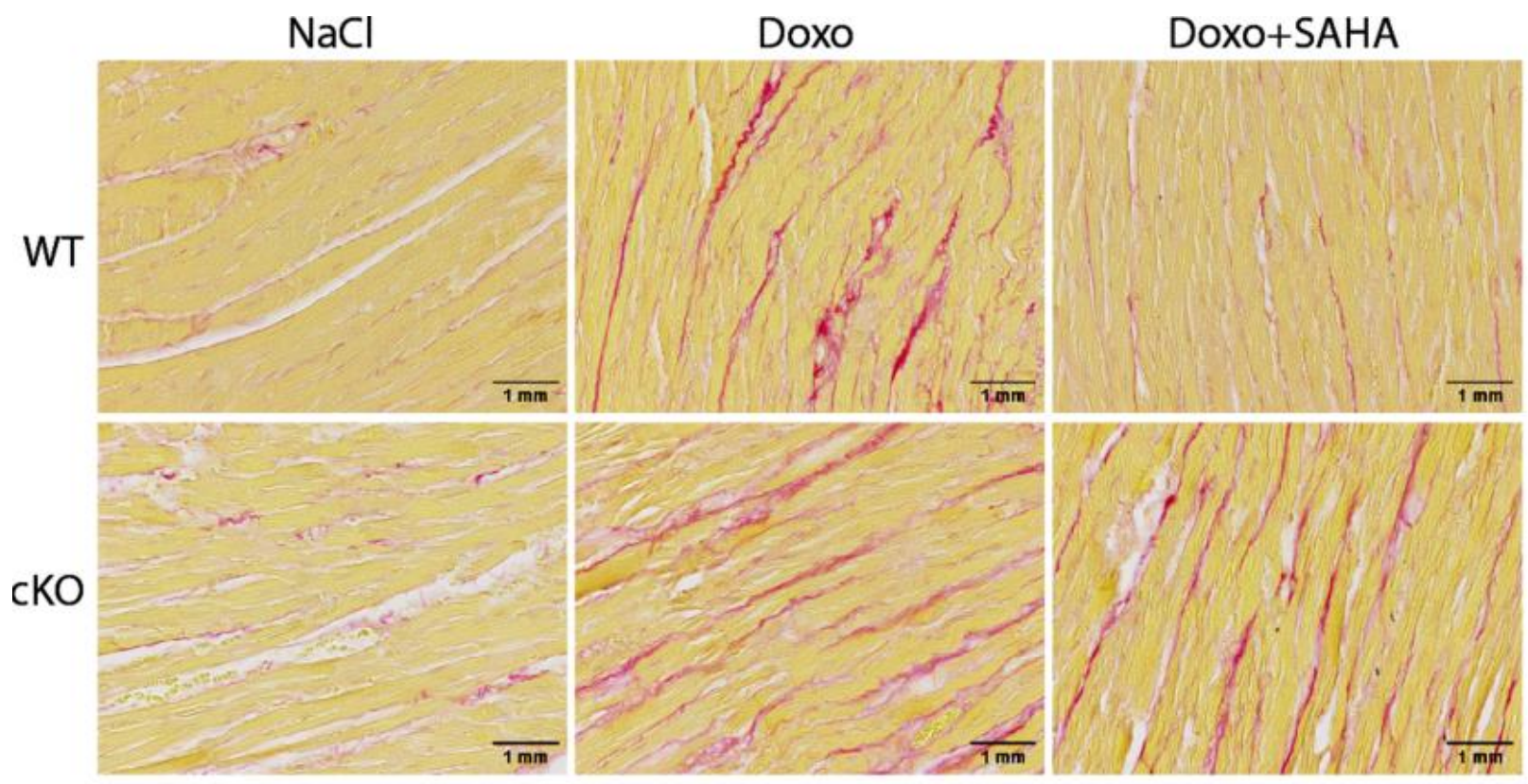

8. The Discussion section should be double-checked for spelling errors.

We went through the discussion section and removed typos and spelling errors.

References

1. Tobin, S. W., *et al.* Heart Failure and MEF2 Transcriptome Dynamics in Response to beta-Blockers. *Sci Rep* **7**, 4476 https://doi.org/10.1038/s41598-017-04762-x (2017).
2. Maillet, A., *et al.* Modeling Doxorubicin-Induced Cardiotoxicity in Human Pluripotent Stem Cell Derived-Cardiomyocytes. *Sci Rep* **6**, 25333 https://doi.org/10.1038/srep25333 (2016).
3. Pang, Y., *et al.* The combination of the HDAC1 inhibitor SAHA and doxorubicin has synergic efficacy in triple negative breast cancer in vivo. *Pharmacol Res* **196**, 106926 https://doi.org/10.1016/j.phrs.2023.106926 (2023).
4. Lee, S. J., *et al.* Transactivation of bad by vorinostat-induced acetylated p53 enhances doxorubicin-induced cytotoxicity in cervical cancer cells. *Exp Mol Med* **46**, e76 https://doi.org/10.1038/emm.2013.149 (2014).
5. Schmitt, T., *et al.* Vorinostat in refractory soft tissue sarcomas - Results of a multi-centre phase II trial of the German Soft Tissue Sarcoma and Bone Tumour Working Group (AIO). *Eur J Cancer* **64**, 74-82 https://doi.org/10.1016/j.ejca.2016.05.018 (2016).
6. Collins, P. L., *et al.* DNA double-strand breaks induce H2Ax phosphorylation domains in a contact-dependent manner. *Nat Commun* **11**, 3158 https://doi.org/10.1038/s41467-020-16926-x (2020).
7. Iacovoni, J. S., *et al.* High-resolution profiling of gammaH2AX around DNA double strand breaks in the mammalian genome. *EMBO J* **29**, 1446-1457 https://doi.org/10.1038/emboj.2010.38 (2010).
8. Lian, X., *et al.* Robust cardiomyocyte differentiation from human pluripotent stem cells via temporal modulation of canonical Wnt signaling. *Proc Natl Acad Sci U S A* **109**, E1848-1857 https://doi.org/10.1073/pnas.1200250109 (2012).
9. Villalobos, V. M., *et al.* Pharmacokinetics of doxorubicin following concomitant intravenous administration of olaratumab (IMC-3G3) to patients with advanced soft tissue sarcoma. *Cancer Med* **9**, 882-893 https://doi.org/10.1002/cam4.2728 (2020).
10. Amgalan, D., *et al.* A small-molecule allosteric inhibitor of BAX protects against doxorubicin-induced cardiomyopathy. *Nat Cancer* **1**, 315-328 https://doi.org/10.1038/s43018-020-0039-1 (2020).

The authors have done an excellent job with this revision. I have only one minor comment:

The new data presented in Supplementary Figure 8 are very helpful. However, in panel E, it appears as though there is more TUNEL staining in Doxo + SAHA mice compared to Doxo alone. This is likely because of differences in cardiomyocyte density between the various panels (including the NaCl panel as well). It would be helpful to show images that are more representative of the summary data presented in panel F.

We thank the reviewer for this helpful suggestion and agree that the originally selected representative image for the Doxo+SAHA group may not reflect the quantification in Supplementary Figure 7F. We have therefore replaced the representative images. Further, we rearranged the images according to the quantification order. (Supplementary Figure 8 has become Supplementary Figure 7).

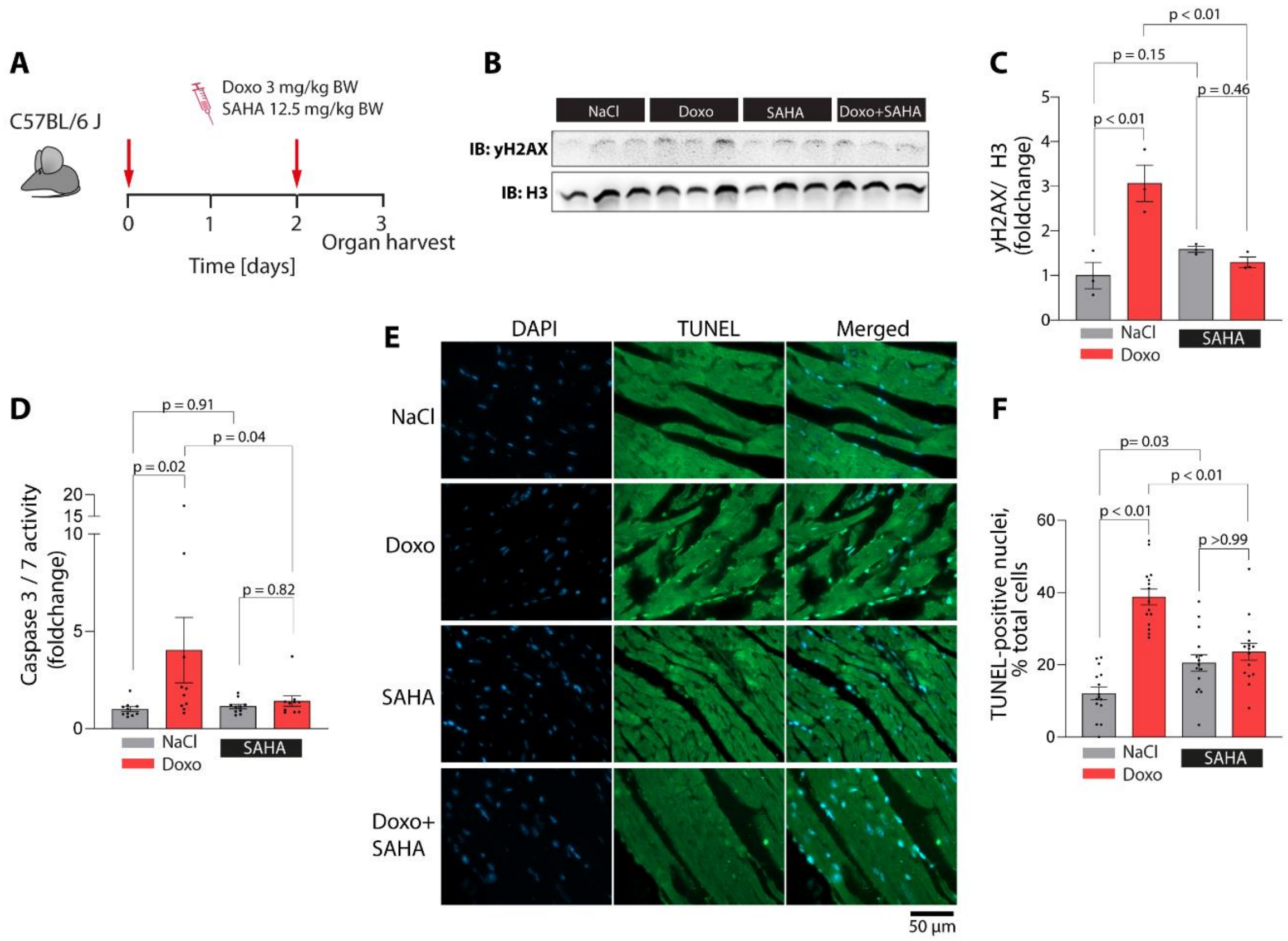

Supplementary Figure 7: (A) Experimental design of the in vivo short-term treatment protocol in C57BL/6J mice receiving doxorubicin (3 mg/kg BW) SAHA (12.5 mg/kg BW). **(B)** Representative immunoblot analysis of γH2AX expression in cardiac tissue from NaCl, Doxo ±SAHA-treated mice. Histone H3 served as loading control. **(C)** Quantification of γH2AX protein levels normalized to H3 (n=3 mice per treatment group. Data are presented as mean ± SEM. Statistical analysis was performed using one-way ANOVA followed by Bonferroni's multiple comparisons test. Exact p-values for the indicated comparisons were: NaCl vs. Doxo, p = 0.0005; NaCl vs. SAHA, p =

0.1499; Doxo vs. Doxo + SAHA, $p = 0.0014$; SAHA vs. Doxo + SAHA, $p = 0.4569$ **(D)** Caspase-3/7 activity in cardiac tissue lysates from NaCl, Doxo ±SAHA-treated mice (n= 10 mice per treatment group). Data are presented as mean ± SEM. Statistical analysis was performed using one-way ANOVA followed by Bonferroni's multiple comparisons test; exact p-values are indicated above the comparisons. **(E)** Representative images of TUNEL staining in cardiac sections obtained from NaCl, Doxo ±SAHA-treated mice. Nuclei were counterstained with DAPI. Scale bar: 50 µm. **(F)** Quantification of TUNEL staining represented as the percentage (%) of TUNEL-positive nuclei of counted total nuclei in n= 3 mice per group. For each mouse, 5 images were taken. Data are presented as mean ± SEM. Statistical analysis was performed using one-way ANOVA followed by Bonferroni's multiple comparisons test. Exact p-values for the indicated comparisons were: NaCl vs. Doxo, $p < 0.0001$; NaCl vs. SAHA, $p = 0.0286$; Doxo vs. Doxo + SAHA, $p < 0.0001$; SAHA vs. Doxo + SAHA, $p > 0.9999$. Source data are provided as a Source Data file.

Reviewer #2 (Remarks to the Author)

I thank the authors for their careful revision. The manuscript has been substantially strengthened through the addition of new mechanistic experiments, additional in vivo data, improved presentation of the functional studies, and a more balanced discussion of the study's limitations. The new TopoIIβ loss-of-function experiments and the additional DNA damage and apoptosis data strengthen the proposed mechanism and improve the overall rigor of the study.

I also appreciate that the authors addressed concerns regarding the presentation of the echocardiographic and fibrosis data by moving less informative panels to the supplement and tempering the interpretation of the in vivo findings.

While not every requested experiment was performed (particularly delayed SAHA treatment), the authors provide a reasonable rationale for focusing on concurrent administration, which aligns with their proposed clinical application. I no longer consider this essential for publication.

Overall, I believe the manuscript has been substantially improved, and I have only a few minor comments.

Minor comments

1. Throughout the Discussion, I encourage the authors to continue distinguishing between findings that directly demonstrate causality and those that remain inferential.

2. The rationale for focusing on concurrent SAHA administration rather than delayed treatment is reasonable. I suggest emphasizing this point more explicitly in the Discussion, as it helps frame the intended translational application and addresses an important potential criticism.

1. We thank the reviewer for this helpful suggestion. We have carefully revised the Discussion accordingly.

2. We thank the reviewer for this valuable suggestion. We have expanded the Discussion to more explicitly explain the rationale for administering SAHA concurrently with doxorubicin. Specifically, we now emphasize that our study was designed to model the clinical application of SAHA as a cardioprotective co-therapy administered during anthracycline treatment to prevent the initiation of cardiotoxic remodeling, rather than to reverse established cardiac injury. We further highlight that this design strengthens the translational relevance of our findings while acknowledging that the effects of delayed therapeutic intervention remain to be investigated.

We have added the following phrase in the Discussion section: *“[…] Importantly, SAHA was administered concurrently with doxorubicin to model its intended use as a cardioprotective co-therapy during anthracycline treatment. Therefore, our study was designed to prevent the initiation of maladaptive remodeling rather than reverse established cardiotoxicity. […]”*
