# Supplementary material for: HDAC inhibition via suberoylanilide hydroxamic acid ameliorates doxorubicin-induced cardiotoxicity
Source: Nat Commun. 2026 Sep 4;17:9505. doi: 10.1038/s41467-026-77428-w (PMC13545280; doi:10.1038/s41467-026-77428-w)

Figure 2K

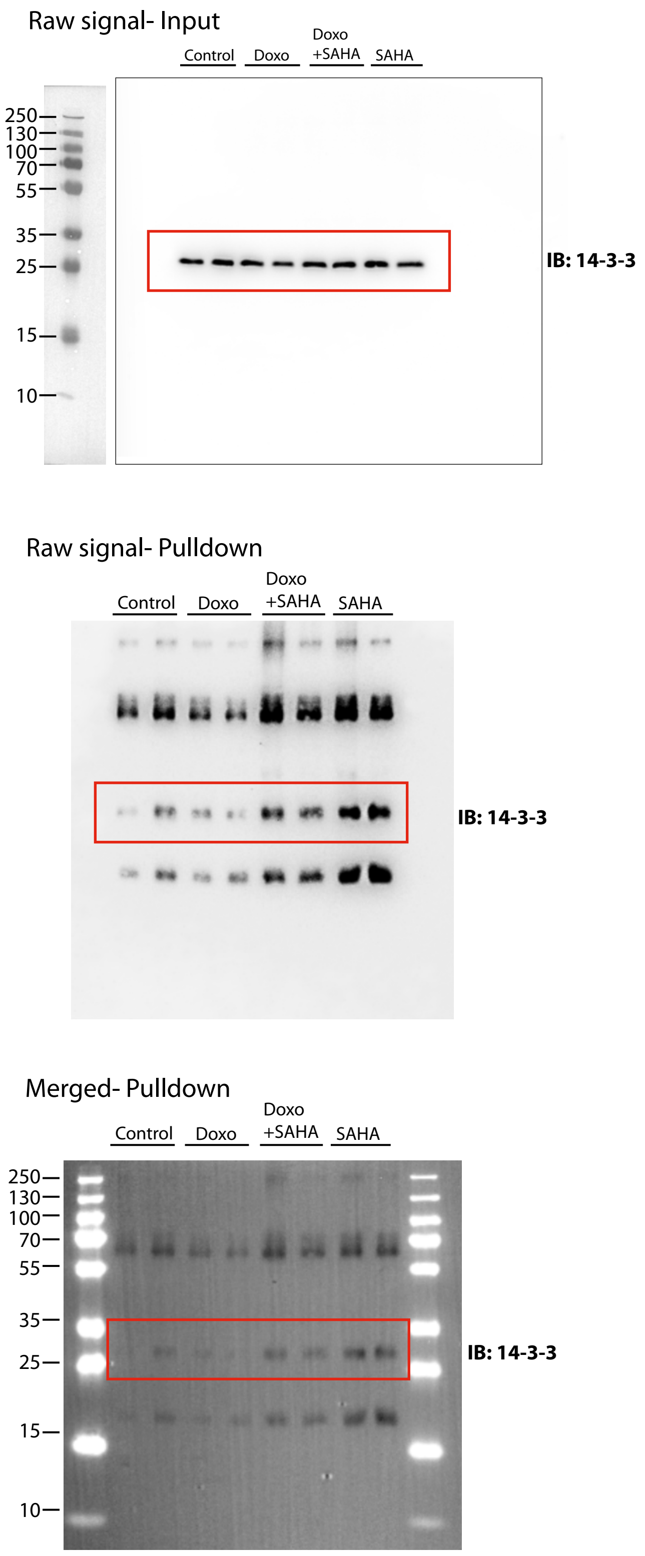

Figure 2L

1 – HDAC4 + WT
2 – HDAC4 + K50Q
3 – HDAC4 + K78Q
4 – HDAC4 + K50/78Q
5 – HDAC4 + K50/78/123Q

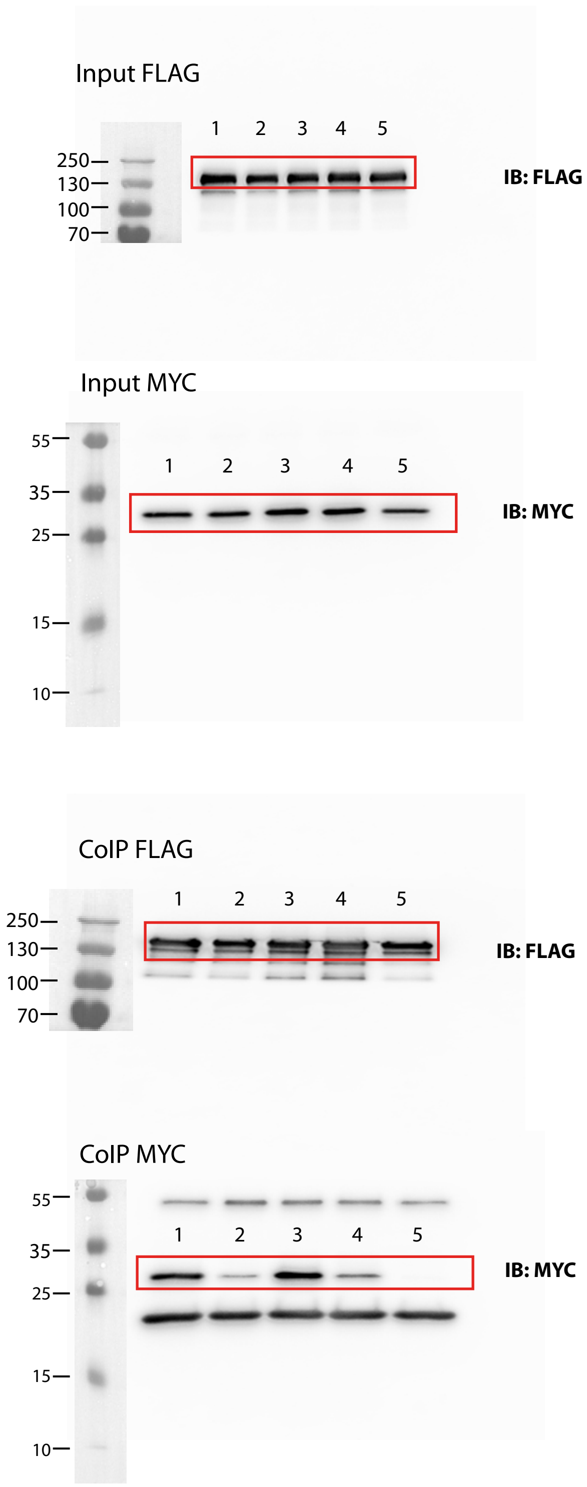

Figure 2M

1 – HDAC5 + WT
2 – HDAC5 + K50Q
3 – HDAC5 + K78Q
4 – HDAC5 + K50/78Q
5 – HDAC5 + K50/78/123Q

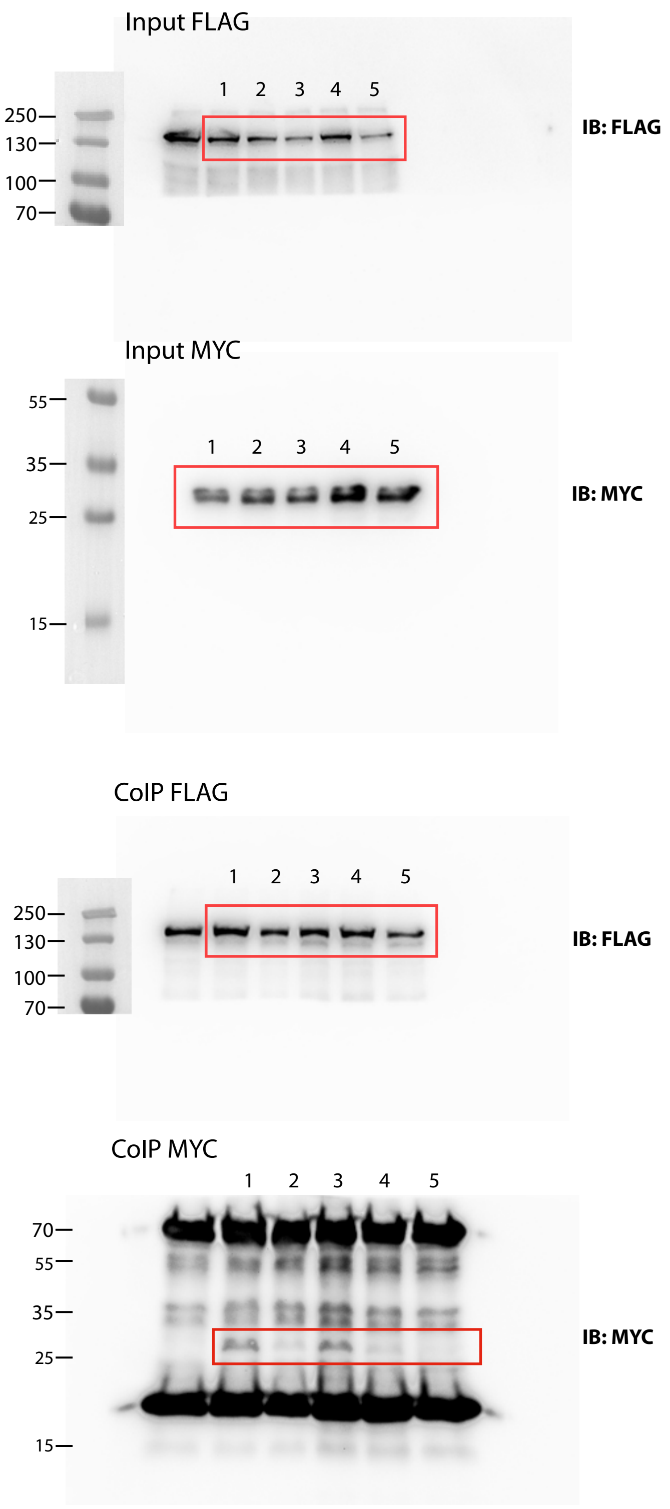

Supplementary Figure 2A

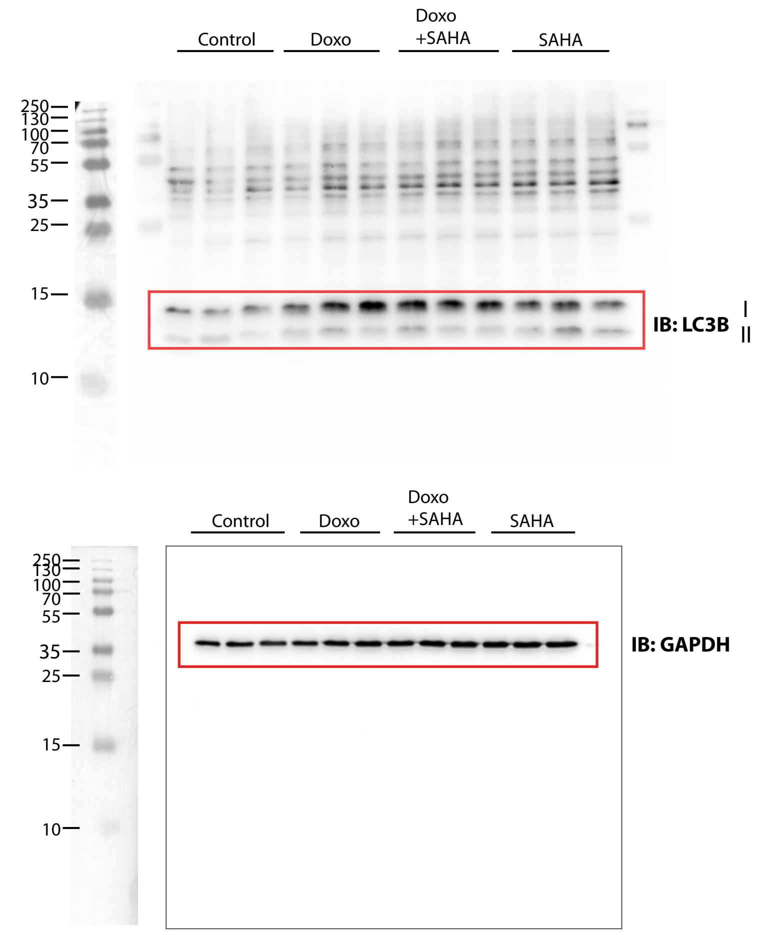

Supplementary Figure 7B

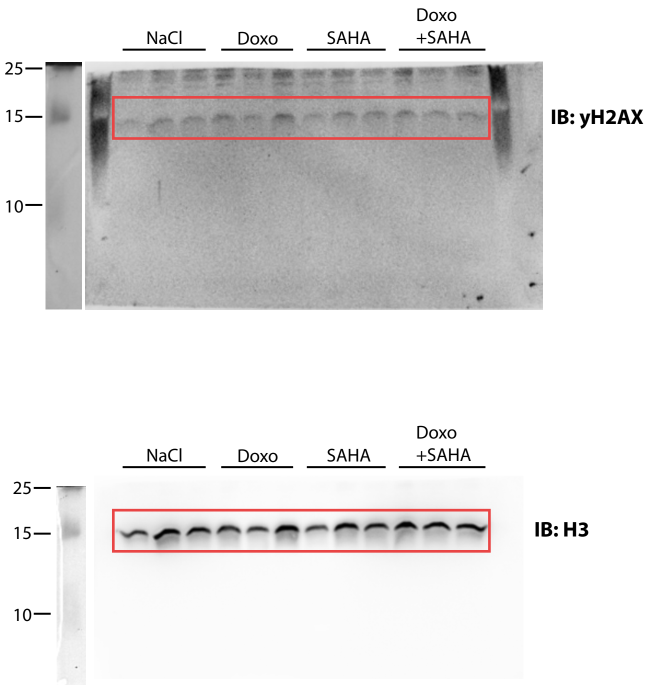

Supplementary Figure 12

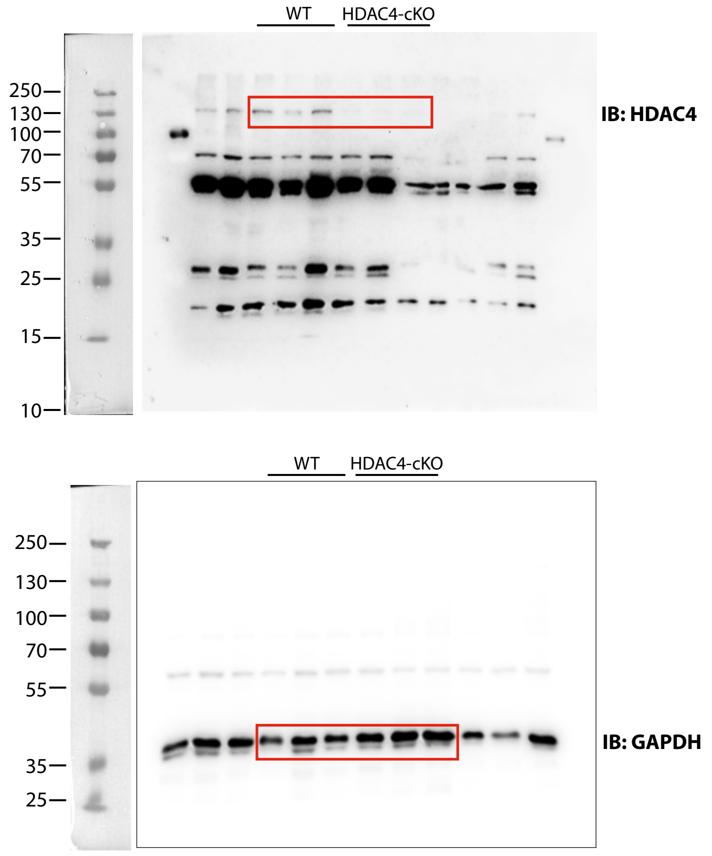

Supplement: Supplementary file 5 [file 41467_2026_77428_MOESM10_ESM.zip › Supplementary Figure.pdf]
